# Development and investigation of a site selective palladium-catalyzed 1,4-difunctionalization of isoprene using pyridine–oxazoline ligands†
†Electronic supplementary information (ESI) available: Detailed experimental procedures, reaction optimization data, and spectroscopic data of all new compounds. See DOI: 10.1039/c4sc03074e



**DOI:** 10.1039/c4sc03074e

**Published:** 2014-11-28

**Authors:** Matthew S. McCammant, Matthew S. Sigman

**Affiliations:** a Department of Chemistry , University of Utah , 315 South 1400 East , Salt Lake City , USA . Email: sigman@chem.utah.edu ; Fax: +1-801-681-8433 ; Tel: +1-801-585-0774

## Abstract

Palladium-catalyzed 1,4-difunctionalizations of isoprene that produce skipped polyenes are reported. Complex isomeric product mixtures are possible as a result of the difficult-to-control migratory insertion of isoprene into a Pd–alkenyl bond, but good site selectivity has been achieved using easily accessible pyrox ligands. Mechanistic studies suggest that the control of insertion is the result of the unique electronic asymmetry and steric properties of the ligand.

## Abstract 2

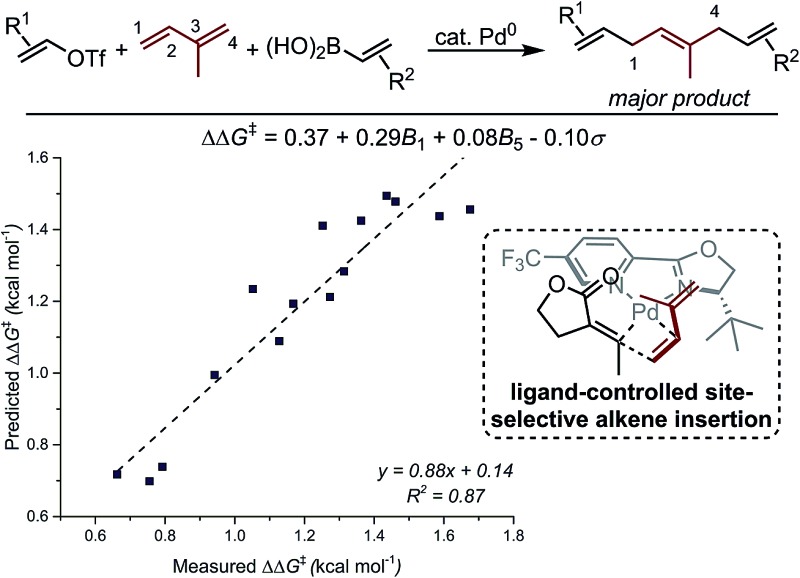
Palladium-catalyzed 1,4-difunctionalizations of isoprene that produce skipped polyenes are reported.

## Introduction

**Fig. 1:**
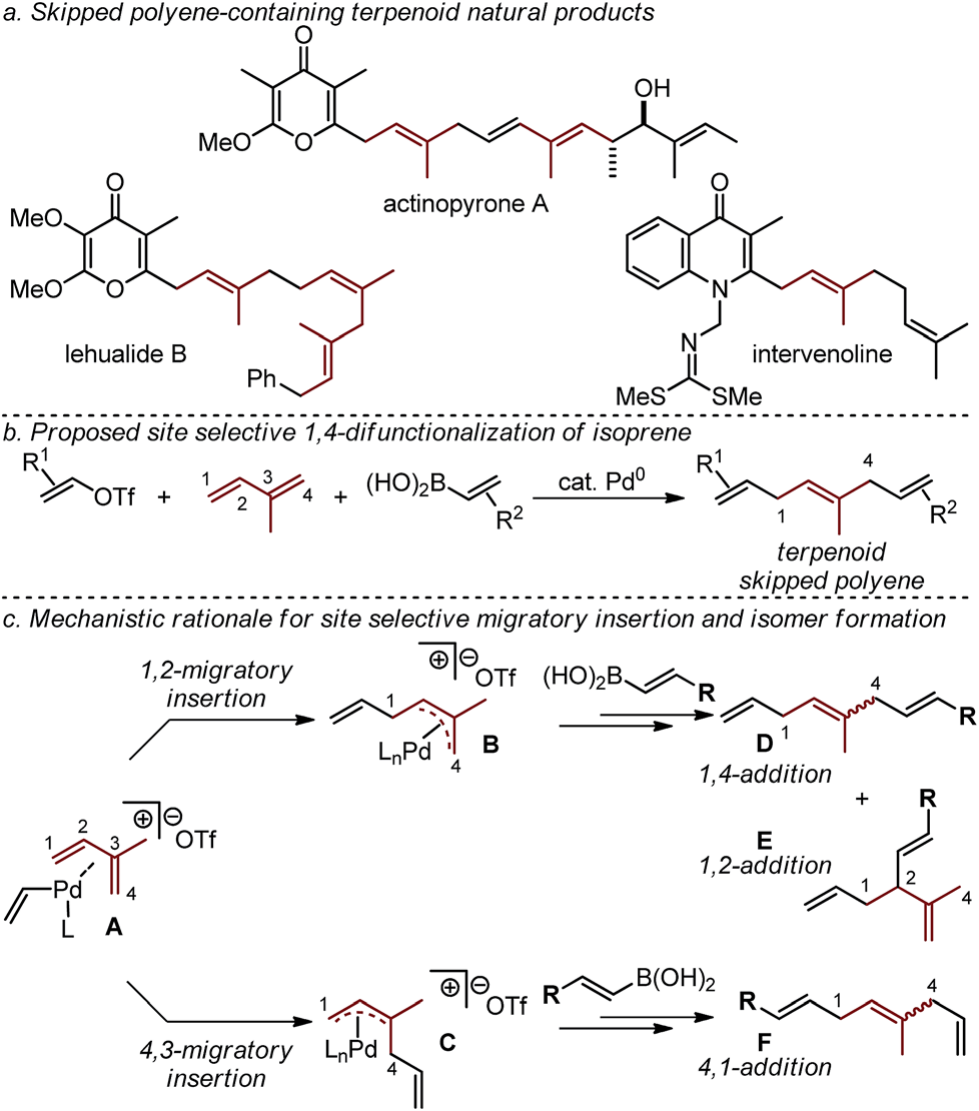
Proposed 1,4-difunctionalization of isoprene and rationale accounting for regioisomers.

Terpenoid natural products exhibit a broad range of important physiological effects. In such molecules, isoprene units are often paired with diverse stereodefined di- and tri-substituted alkenes to give rise to a larger class of molecular frameworks known as skipped polyenes (Fig. 1a).^1–10^ Unfortunately, the rapid assembly of stereochemically-defined skipped polyenes remains a significant challenge in modern synthetic chemistry.

We have recently reported a Pd-catalyzed 1,4-difunctionalization reaction of 1,3-butadiene with alkenyl triflates, and aryl or alkenylboronic acids that enabled the rapid assembly of diverse skipped polyene frameworks.^11^ In an effort to advance this strategy to directly access skipped polyene-containing terpenoid fragments in a single step, we sought to utilize isoprene as the 1,3-diene substrate (Fig. 1b). The effective use of isoprene in such a three-component coupling reaction would require us to address the added challenge arising from the use of a diene substrate containing two similar, yet distinct alkenes. As others have reported,^12^ site selective 1,4-addition (as opposed to 4,1-addition, Fig. 1c) to isoprene is dependent on a difficult-to-control alkene migratory insertion into the cationic Pd-alkenyl intermediate **A**. The desired 1,4-difunctionalization product **D** is hypothesized to be accessed upon insertion of the less-substituted alkene of isoprene to give a cationic π-allyl palladium intermediate (**B**). This is followed by transmetallation with an alkenylboronic acid, and reductive elimination to yield **D** (or the 1,2-addition product **E**). Isomeric mixtures derived from difunctionalization reactions of simple 1,3-dienes give 1,2-products typically as the minor isomer, with some notable exceptions.^13^ Additionally, formal 4,3-addition products (not shown) are not frequently observed as a result of the opposite alkene insertion pathway (**A** → **C** → **F**), likely due to a high barrier for reductive elimination of a quaternary center from palladium. In total, five distinct constitutional and stereoisomers^11–14^ can be formed in this three-component coupling reaction through likely energetically similar pathways. The cationic nature of the palladium catalyst, resulting after oxidative addition of an alkenyl triflate, is proposed to account for the high selectivity of three-component coupling products rather than Heck or Suzuki products (not shown).^11,14^ Herein we report a Pd-catalyzed 1,4-difunctionalization, which utilizes pyrox ligands to afford a site preference for isoprene migratory insertion.

## Results and discussion

Starting with the reaction conditions we reported for the Pd-catalyzed 1,4-difunctionalization of 1,3-butadiene,^11^ only minor adjustments were required to increase yield and selectivity for the formation of the (*E*)-**4a**.^15^ Most significantly, an increase in the amount of isoprene (7.0 equivalents) was required to achieve a good yield of alkene difunctionalization products when coupling to cyclohexenyl triflate (**1a**) and styrenylboronic acid (**3a**) with isoprene. Under the indicated optimal conditions, good yield and selectivity for the formation of (*E*)-**4a** was observed (Table 1). Of note, all of the isomeric products can be separated by HPLC and a 61% yield of the desired isomer is achieved.

**Table 1:** Initial scope of the Pd-catalyzed 1,4-difunctionalization of isoprene^*a*^

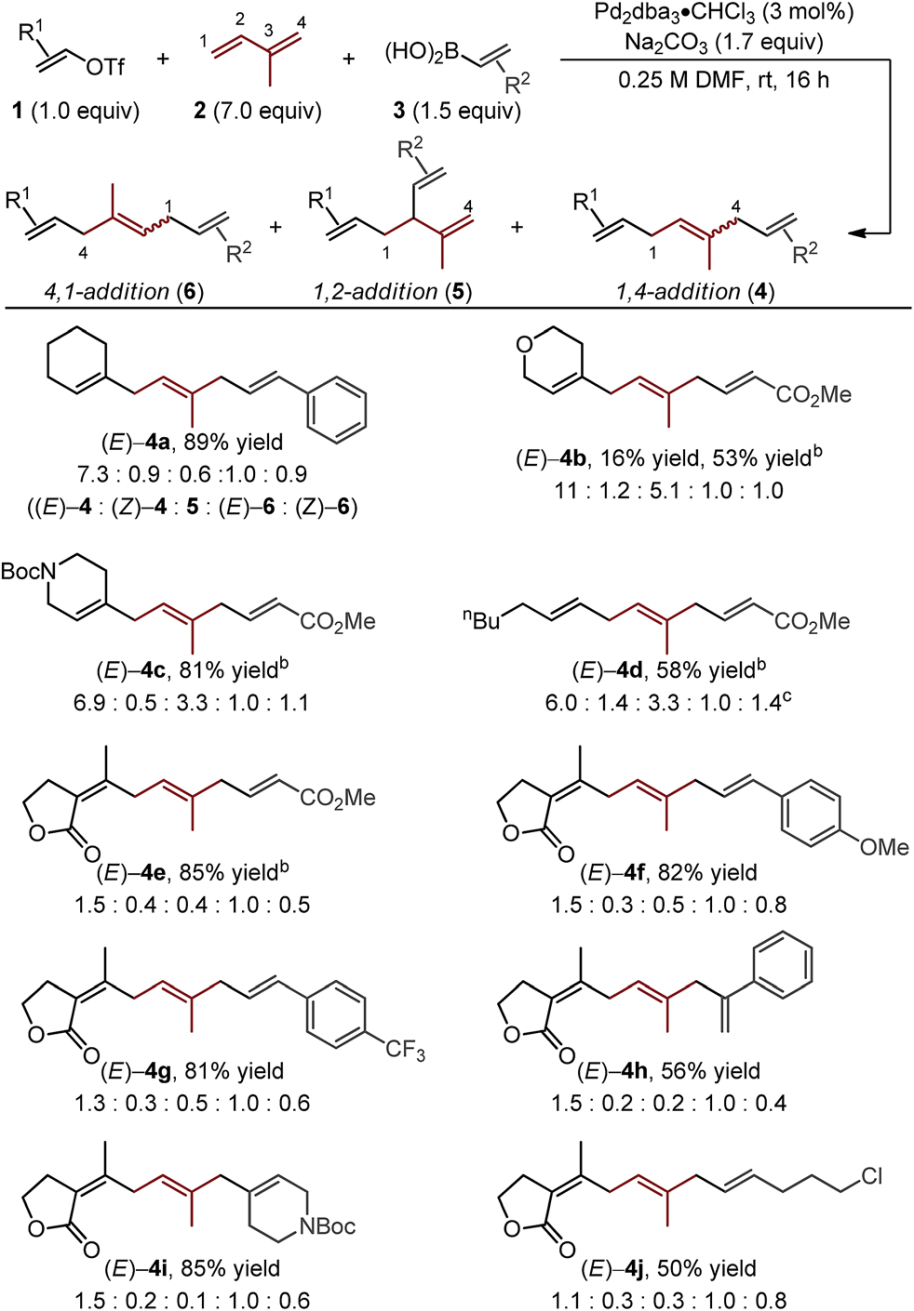

^*a*^Yields are reported as a combination of isomers of reactions performed on a 0.5 mmol scale. Structures of isomers were confirmed by separation using HPLC and NMR analysis. Isomeric ratios were determined by either ^1^H NMR or HPLC.

^*b*^Reaction performed with 3.0 equivalents of **1** and 1.0 equivalents of **3**.

^*c*^(*Z*)-**4d** and (*Z*)-**6d** were inseparable by HPLC and ^1^H NMR signals overlapped. Thus, values are reported as a mixture.

The assessment of alkenyl triflates yielded products incorporating a tetrahydropyranyl (**4b**), a N-protected piperidinyl (**4c**) heterocycles and a simple aliphatic (**4d**), which revealed similar selectivity for the formation of (*E*)-**4** relative to (*E*)-**6** (>6 : 1). Under the standard optimized conditions, lower yields were observed of skipped polyene products when using a conjugated ester derived boronic acid to yield **4b**. We hypothesize that this could be due to either catalyst inhibition by the boronic acid or the product through competitive binding to the catalyst. By simply increasing the concentration of the alkenyl triflate, significantly improved yields were observed (**4b–e**).^16^

Next, we varied the alkenylboronic acid component, specifically coupling styrene-(**4f–h**), N-heterocycle-(**4i**), and alkyl halide-containing (**4j**) boronic acids to an electron-deficient alkenyl triflate (**1e**) and isoprene. Triflate **1e** was evaluated because the vinylogous lactone is an attractive synthetic handle for further elaboration. While the boronic acids minimally influenced the product yields and isomeric ratios, alkenyl triflate **1e** proved to impact selectivity greatly compared to other alkenyl triflates (**4f–j**). Specifically, the ratio of (*E*)-**4** : (*E*)-**6** was reduced to nearly 1 : 1 in these examples. This shortcoming is addressed below.

Other important observations regarding the Pd-catalyzed three-component difunctionalization of isoprene can be discerned from Table 1. For example, (*E*)-**4h** was formed in adequate yield from 1-phenylvinylboronic acid. This is somewhat surprising since 1,1-disubstituted terminal alkenes are known to undergo migratory insertion into Pd–alkenyl bonds like that of **A** (Fig. 1c),^12,13a,17^ but are tolerated under our reaction conditions. Additionally, higher selectivity for (*E*)-**4h** and (*E*)-**4i** over 1,2-addition products **5h** and **i** (8.8 : 1 and 11 : 1 respectively) is observed for nonlinear boronic acids coupling partners (compared to other alkenylboronic acids, all of which are linear). This may be due to an added steric influence on the Pd–π-allyl (**B** in Fig. 1c), thereby promoting 1,4-addition as compared to 1,2-addition.^18^

**Fig. 2:**
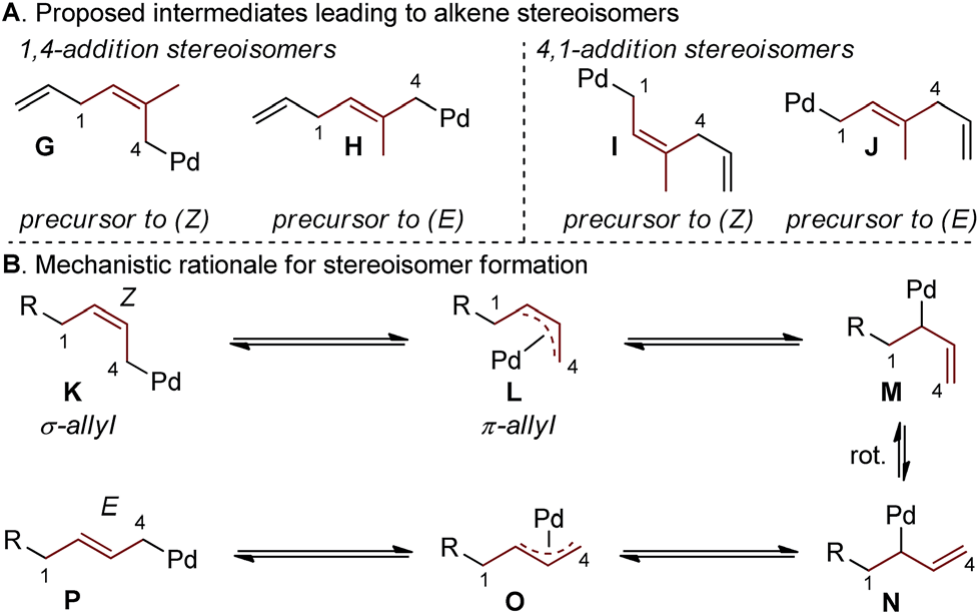
Rationale accounting for stereoisomer formation.

Interestingly, the relative abundance of the (*E*)-stereoisomers of **4** and **6** were only modestly dependent on the coupling partners used in these reactions: (*E*)-**4** consistently predominated over (*Z*)-**4**, while (*E*)-**6** and (*Z*)-**6** were consistently formed in nearly equal amounts. An explanation for this observation stems from the steric environment about the relevant σ-allyl-stabilized palladium intermediates (Fig. 2a, compare **G** to **H** and **I** to **J**), which can interconvert through a σ → π → σ process.^11–14,18^ A simplified example of this is shown in Fig. 2b, wherein σ-allyl palladium intermediate **K** (precursor to the (*Z*)-stereoisomer) can form σ-allyl palladium **P** (precursor to the (*E*)-stereoisomer) after a key bond rotation that occurs between intermediates **M** and **N** (Fig. 2). Similar processes occur for isomer formation in the 4,1-addition scenario through intermediates **I** and **J** to form (*Z*)-**6** and (*E*)-**6**, respectively. The comparable steric environment about σ-allyl intermediates that lead to (*E*)-**6** or (*Z*)-**6** accounts for the nearly equal amounts of the 4,1-stereoisomers.

The aforementioned undesired 1 : 1 ratio of (*E*)-**4** : (*E*)-**6** suggests indiscriminate alkene insertion into the Pd–alkenyl intermediate **A** (Fig. 1c). From an electronic prospective, triflates **1a–d** are comparatively electron-rich (unstabilized) as opposed to **1e**, which may play an important role in selective alkene insertion into the Pd–alkenyl intermediate **A** (*i.e.*, (*E*)-**4a** : (*E*)-**6a** 7.3 : 1 compared to (*E*)-**4e** : (*E*)-**6e** 1.5 : 1). Due to the potential utility of electron deficient (stabilized) alkenyl triflates, we sought to address the challenge of indiscriminate alkene insertion, and, in doing so, potentially regain control over the selectivity.

Electronically-stabilized alkenyl groups would be expected to render the cationic Pd-intermediate **A** (Fig. 1c) more sensitive to the differential nature of the two alkenes in isoprene, with insertion of the more electron-rich disubstituted alkene of isoprene resulting in increased formation of the undesired 4,1-addition products. In this scenario, a ligand might easily override the inherent electronic bias of substrate insertion. Fortunately, in the course of our initial reaction optimization to produce **4a**, we observed that several ligands were tolerated, although their use led to similar product distribution and generally lower yields as compared to the “ligandless” conditions ultimately employed in Table 1.^15^ Thus, we sought to evaluate the propensity of ligands to override the 1 : 1 selectivity of (*E*)-**4** : (*E*)-**6** products observed for **4e–j**.

To explore this possibility, we evaluated the ability of ligands to empower the selective formation of (*E*)-**4f** (Table 2).^19,20^ The use of monodentate ligands, 4-dimethylaminopyridine (DMAP) or a simple oxazoline (**L1** and **L2**), afforded little enhancement in the isomeric ratios as compared to the “ligandless” conditions. The bidentate quinolone–oxazoline (quinox) ligand (**L3**) led to a two-fold increase in selectivity of (*E*)-**4f** over (*E*)-**6f** (2.1 : 1), albeit in low yield. A related ligand, chiral pyridine–oxazoline (pyrox) **L4**, which has been used with success in recent Pd-catalyzed redox-relay Heck reactions in our lab,^21^ significantly enhanced selectivity between (*E*)-**4f** and (*E*)-**6f** (7.7 : 1). Evaluation of **L5** reveals the importance of one unhindered catalyst face, as a low yield and modest selectivity is observed when a geminal dimethyl-substituted ligand is used. Other similar N,N-type ligands were examined including the CF_3_-substituted quinox **L6** and the 6-CF_3_-substituted pyrox **L7**, both of which afford low selectivity. Lastly, an interesting result is observed when ligand **L8** is employed: selectivity between the three major regioisomers favor the formation of **5f** (1,2-addition product), albeit in reduced yield. Unfortunately, the selectivity between the 1,4-addition and 1,2-addition products (**4f** and **5f**) did not exceed 4 : 1 with any of the ligands that were evaluated.

**Table 2:** Ligand evaluation for the Pd-catalyzed difunctionalization of isoprene^*a*^

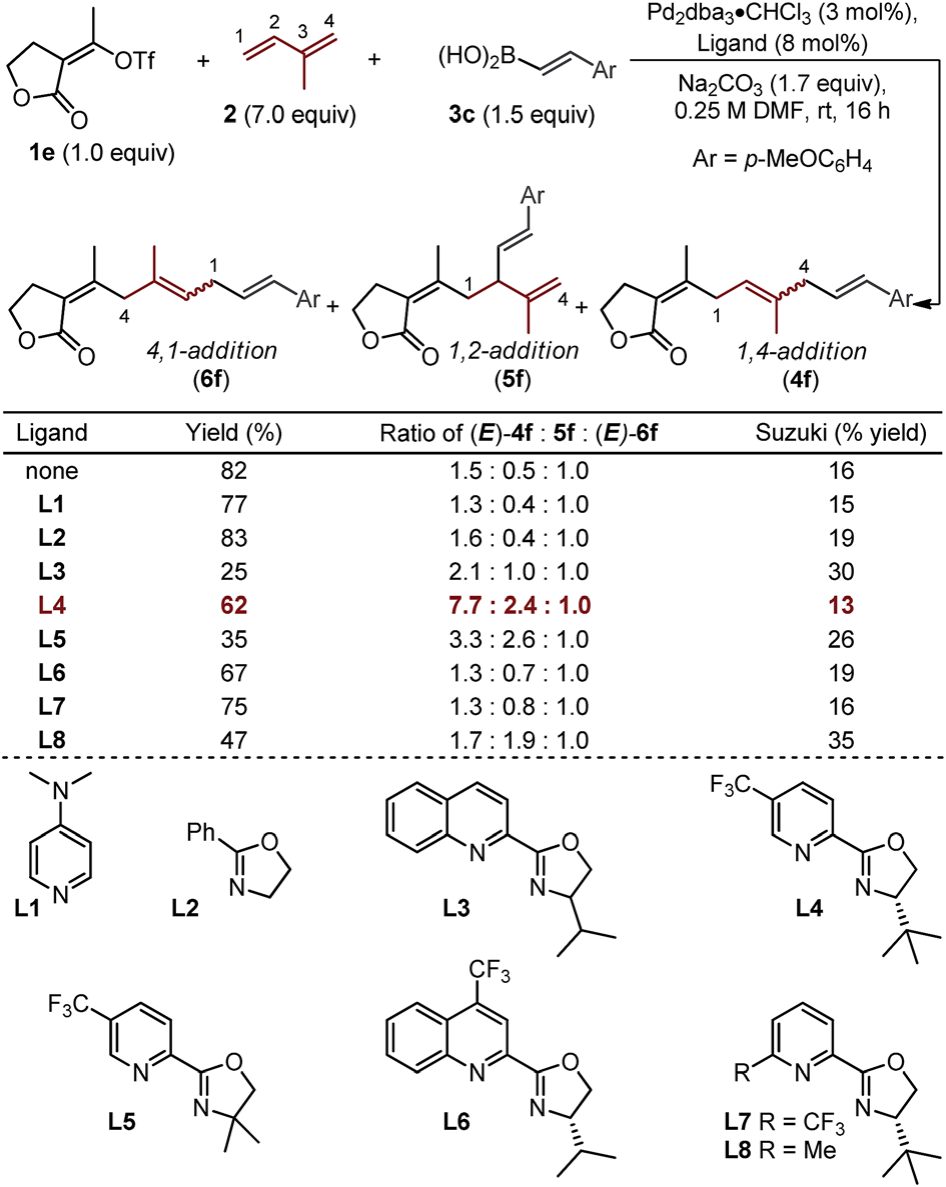

^*a*^Yields are reported as a combination of isomers for reactions performed on 0.2 mmol scale. Yields and isomeric ratios as determined by ^1^H NMR using an internal standard.

**Fig. 3:**
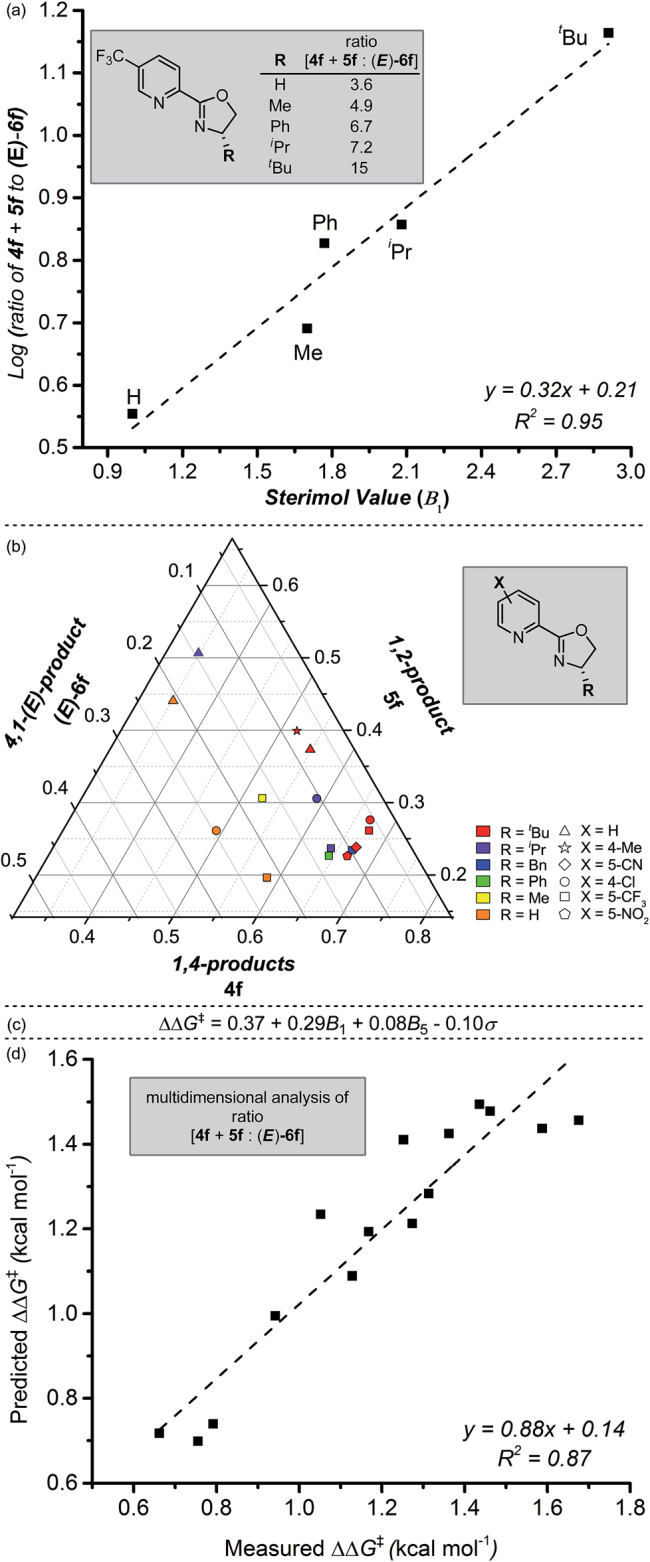
(a) Correlation between site selectivity of alkene insertion and Sterimol *B*_1_ values. (b) Ternary plot of normalized isomeric product distribution resulting from pyrox ligand screen. (c) Normalized mathematical relationship describing differential free energy of alkene insertion selectivity. (d) Predicted *versus* measured ΔΔ*G* ^‡^ plot derived from Sterimol *B*_1_ values, Sterimol *B*_5_ values, and Hammett *σ* values for site selectivity of alkene insertion.

We next sought to investigate the mechanistic basis for the putative site-selective migratory insertion that occurs in the presence of **L4**. Using the same reactants and conditions found in Table 2, a library of easily-accessible pyrox ligands was evaluated with varying oxazoline substitution. From these experiments, a trend in alkene insertion was observed based on the size of the R-substituent on the oxazoline portion of the ligand (Fig. 3a). Of the ligands evaluated, those featuring smaller R-groups afforded diminished selectivity for the formation of the 1,4- and 1,2-products compared to the (*E*)-4,1-product (3.6 : 1 for R = H, compared to 15 : 1 for R = *t*-Bu). Indeed, a correlation of the logarithm of product selectivity (corresponding to the presumed relative rate of insertion) *versus* Sterimol *B*_1_ values^22^ (minimum radius corresponding to the pyrox ligand R-substituent) is observed. This suggests that the oxazoline's steric environment is partially responsible for the observed site selection.

Satisfied with our observation of this clear ligand steric effect, we turned to our recently-developed methodology of combining design of experiments with multi-parameter ligand modulation.^23^ In this case, we evaluated both electronic effects on the pyridine ring and steric modifications on the oxazoline simultaneously. By using such an experiment, we hoped to ascertain which of these factors was most influential in the observed selectivity for 1,4- and 1,2-addition *versus* 4,1-addition. To analyse the results, ratios between **4f**, **5f**, and (*E*)-**6f** were normalized and plotted by way of a ternary plot as a means to identify general trends in the isomeric distribution (Fig. 3b). As illustrated, the highest selectivity for 1,4-addition (**4f**) is observed with ligands combining bulky R-substituents and electron-deficient pyridine rings. To delineate these observed steric and electronic effects on the reaction outcome, a precise mathematical model was needed; Sterimol and Hammett values were chosen as our respective descriptors. We then employed a standard stepwise linear regression algorithm to expedite statistical exploration of the relationship between these parameters and ΔΔ*G* ^‡^ (experimentally-derived, and equalling –*RT* ln(**4f** + **5f** : (*E*)-**6f**), where *R* is the ideal gas constant and *T* is temperature). The resultant normalized equation and a plot of measured *versus* predicted ΔΔ*G* ^‡^ values is depicted in Fig. 3c and d. The relatively high *R* ^2^-value as well as the slope nearly equal to one, validates the strength of the model.

To evaluate the influence of each specific parameter, the coefficients can be compared. As observed in Fig. 3a, the largest coefficient belongs to the Sterimol *B*_1_ parameter (minimum radius of the oxazoline R-substituent), again suggesting a strong influence of substituent size on site selection. The coefficient relating to Hammett *σ*-values gives us information that the electronics of the pyridyl ring are important to influencing selectivity as well. As articulated in previous studies^21,24^ from our lab, these results suggest that the electronic asymmetry of the pyrox ligands likely impart control of the catalyst coordination sphere, thus influencing insertion site selection, and therefore product outcome. Furthermore, virtual extrapolation of the model to predict a better catalyst was investigated, but no reasonable improvements could be identified following intuitive and accessible changes to the ligand structure.

**Fig. 4:**
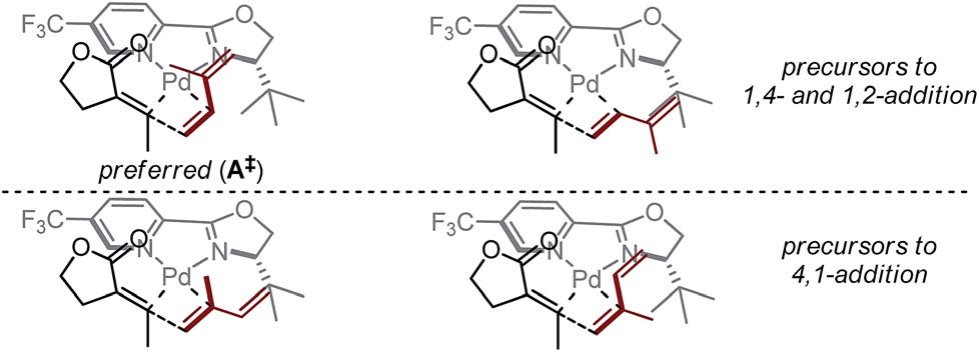
Proposed mechanistic model accounting for observed alkene insertion site selectivity.

Based on the above-presented studies, a mechanistic model is proposed for the observed ligand-control over alkene insertion into a Pd–alkenyl bond. Previously reported computations on Heck reactions using **L4** suggest the coordination environment of a cationic Pd–aryl intermediate following oxidative addition is rapidly isomerizing and, as such, the coordination environment would be dictated by the relative energies of the different alkene insertion transition states.^25^ In that report, the transition states for insertion were computed to be more favourable by nearly 1 kcal mol^–1^ for the complexes wherein the oxazoline and the arene are oriented *trans* to one another. For our purposes, this type of orientation could also explain the significant influence of the oxazoline substituent on the observed selectivity of isoprene insertion into a Pd–alkenyl intermediate. The steric effect generated by the *tert*-butyl group of the ligand and the highly electrophilic nature of palladium (aided by the electron-deficient pyridyl group of the ligand) likely promotes rapid alkene association/dissociation, such that each of the corresponding coordination complexes to the proposed transition states is in equilibrium (Fig. 4). Thus, we propose that the transition state of the selectivity-determining step is controlled by the influence of the *tert*-butyl group on isoprene. Assuming coordination of the alkene *trans* to the pyridine ring, the preferred insertion should occur through **A^‡^**, wherein the likely steric interactions of isoprene insertion are minimized, consistent with the correlative information obtained. This mechanistic model contrasts the original hypothesis (Fig. 1c), wherein isoprene binds in a cisoid-type coordination mode.

**Fig. 5:**
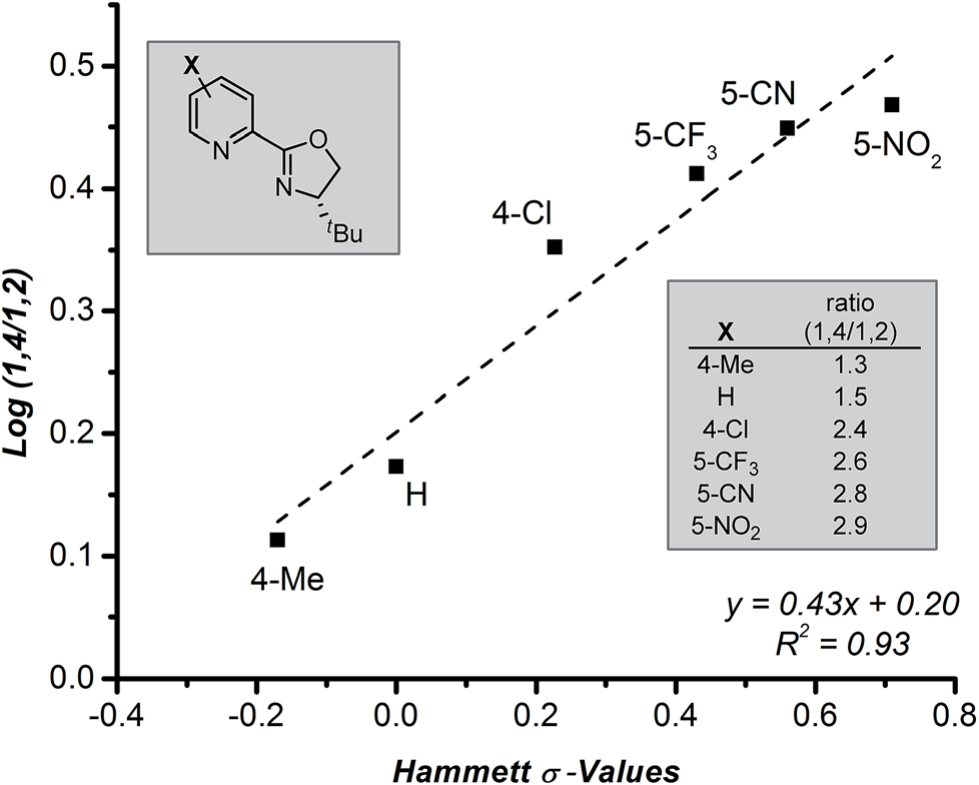
Correlation between 1,4- and 1,2-addition regioisomers and Hammett values.

Further evaluation of the raw data obtained in the ligand survey revealed an additional relationship between the formation of the 1,4- and 1,2-addition products. These products arise from a common intermediate and are only differentiated by the reaction of the π-allyl-stabilized palladium intermediate with a transmetallation partner prior to reductive elimination (Fig. 1c, **B** → **D** + **E**). The observed correlation shows a modest electronic effect on the ratio of products, which can be quantified using Hammett *σ*-values of the pyridyl substituent (Fig. 5). Specifically, greater amounts of the 1,4-addition product are formed as the catalyst becomes more electron deficient, although overall yield sharply decreases for pyrox ligands bearing either a 5-CN or a 5-NO_2_ group (not shown). While the origin of this effect is not clear and will require further investigation, the discovery of electronic control will likely impact future ligand design.

After evaluating a diverse group of pyrox ligands (Fig. 3b), **L4** remained the most selective and also provided modest yields of the desired skipped polyene products. A brief re-optimization of the reaction conditions resulted in lowering the stoichiometry of **L4** as well as increasing the reaction temperature.^26^ Select reactions presented in Table 1 were repeated under the new conditions to evaluate the utility of **L4** toward a more selective process (Table 3). We were pleased to see selectivity improve throughout, and especially for the “stabilized” alkenyl triflates. In conjunction with enhanced selectivity, yields were also similar when using **L4**. However, the formation of the 1,2-addition product still accounts for a considerable amount of the mass-balance, reducing the ability to access the desired product, (*E*)-**4**, in higher yields.

**Table 3:** Scope of ligand controlled 1,4-difunctionalization of isoprene^*a*^

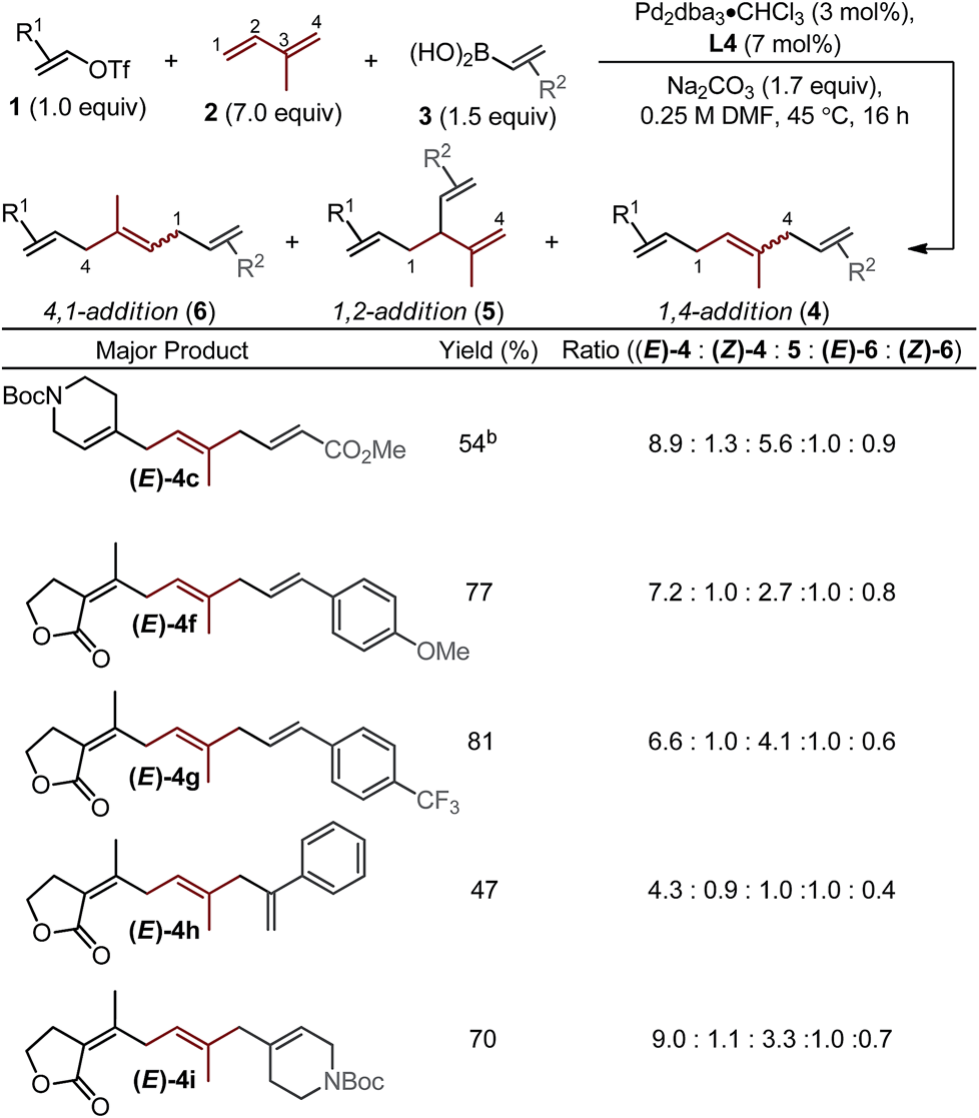

^*a*^Yields are reported as a combination of isomers of reactions performed on a 0.5 mmol scale. Isomeric ratios were determined by ^1^H NMR.

^*b*^Reaction performed with 3.0 equivalents of **1c** and 1.0 equivalent of **3b**.

## Conclusions

In the course of developing this Pd-catalyzed difunctionalization reaction of isoprene, a major influence of coupling partner electronics upon the selectivity of alkene migratory insertion was identified. Through a series of studies, we were able to identify **L4** as a ligand that could enhance control of site selective isoprene insertion into a palladium–alkenyl intermediate. A library of pyrox ligands were examined in order to provide information that ultimately led to a mechanistic rationalization of the role that **L4** plays in controlling alkene insertion. The re-examination of reactions in the presence of ligand afforded better selectivity for the desired 1,4-addition in generally reasonable yields. A limitation in this chemistry remains the confounding formation of 1,2-addition products as a result of a π-allyl-stabilized cationic Pd-intermediate, regardless of the ligand used. Understanding and controlling reaction outcomes of such Pd–π-allyl intermediates in alkene difunctionalization reactions is a focus of on-going studies in our lab.
