# Supplementary material for: Development and investigation of a site selective palladium-catalyzed 1,4-difunctionalization of isoprene using pyridine–oxazoline ligands
Source: Chem Sci. 2014 Nov 28;6(2):1355–61. doi: 10.1039/c4sc03074e (PMC4334162; doi:10.1039/c4sc03074e)
Supplement: Supplementary file 1 [file SC-006-C4SC03074E-s001.pdf]

Electronic Supplementary Material (ESI) for Chemical Science.
This journal is © The Royal Society of Chemistry 2014

Supporting Information

Development and Investigation of a Site Selective Palladium-Catalyzed 1,4-Difunctionalization of Isoprene using Pyridine-Oxazoline Ligands

Matthew S. McCammant and Matthew S. Sigman*

Department of Chemistry, University of Utah, 315 South 1400 East, Salt Lake City, Utah 84112, United States

Table of Contents

S1	I. General Considerations
S1-S5	II. Preparation of Starting Materials
S6-S15	III. Pd-Catalyzed Difunctionalization Reactions
S16	IV. References
S17-S54	V. NMR Spectra for New Compounds

I. General Considerations:

Anhydrous dimethylformamide (DMF) was purchased from Sigma Aldrich and stored over activated 3 Å molecular sieves (3 Å MS). $Pd_2(dba)_3$•$CHCl_3$ was synthesized according to known procedure.[1] Isoprene was purchased from Alfa Aesar and distilled prior to use. Unless otherwise noted all reagents and solvents were purchased from Sigma Aldrich, Frontier Scientific, Acros, or TCI and used without further purification. ^{1}H NMR spectra were obtained at 500 MHz or 400 MHz, chemical shifts are reported in ppm, and referenced to the $CHCl_3$ singlet at 7.26 ppm. The abbreviations s, d, t, q, quint, sext, dd, ddd, dt, and m stand for the resonance multiplicities singlet, doublet, triplet, quartet, quintet, sextet, doublet of doublets, doublet of doublets of doublets, doublet of triplet, and multiplet, respectively. ^{13}C NMR spectra were obtained at 126 MHz and referenced to the centerline of the $CDCl_3$ triplet at 77.23 ppm. Flash chromatography was performed using Silicycle SiliaFlash F60 silica gel (230-400 mesh). Gas Chromatography (GC) separations were performed with an HP6890 GC with a flame ionization detector equipped with a DB-5 column using a 50:1 split. Optical rotations were obtained (Na D line) using a Perkin Elmer Model 343 Polarimeter fitted with a micro cell with a 1 dm path length; concentrations are reported in g/100 mL. HPLC separations were performed with a HP series 1100 chromatograph (Agilent ChromSpher 5 Lipids (4.6 x 250 mm)). IR spectra were recorded using a Thermo Nicolet FT-IR. Melting points were obtained on an electrothermal melting point apparatus and are uncorrected. HRMS data were obtained on a Waters LCP Premier XE instrument by ESI/TOF.

II. Preparation of Starting Materials:

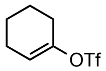

1a

cyclohex-1-en-1-yl trifluoromethanesulfonate 1a.
A previously reported procedure was used for the synthesis of **1a** from cyclohexanone.[2]

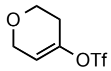

1b

3,6-dihydro-pyran-4-yl trifluoromethanesulfonate 1b.
A previously reported procedure was used for the synthesis of **1b** from tetrahydropyran-4-one.[2]

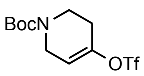

1c

***tert*-butyl 4-(((trifluoromethyl)sulfonyl)oxy)-3,6-dihydropyridine-1-carboxylate 1c**.
A previously reported procedure was used for the synthesis of **1c** from *tert*-butyl-4-oxopiperidine-1-carboxylate.[3]

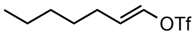

1d

(*E*)-hept-1-en-1-yl trifluoromethanesulfonate 1d.
A previously reported procedure was used for the synthesis of **1d** from *n*-butyllithium and acrolein.[4-6]

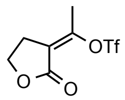

1e

(*Z*)-1-(2-oxodihydrofuran-3-ylidene)ethyl trifluoromethanesulfonate 1e.
A previously reported procedure was used for the synthesis of **1e** from 3-acetyldihydrofuran-2-one.[7]

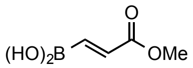

3b

(*E*)-(3-methoxy-3-oxoprop-1-en-1-yl)boronic acid 3b.
A previously reported procedure was used for the synthesis of **3b** from methyl propiolate.[8]

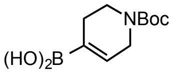

3f

(1-(*tert*-butoxycarbonyl)-1,2,3,6-tetrahydropyridin-4-yl)boronic acid 3f.
A previously reported procedure was used for the synthesis of **3f** from the pinacol boronic ester.[9]

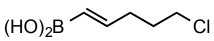

3g

(*E*)-(5-chloropent-1-en-1-yl)boronic acid 3g.
A previously reported procedure was used for the synthesis of **3g** from the pinacol boronic ester.[10]

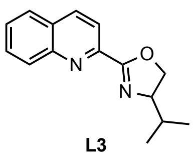

L3

4-isopropyl-2-(quinolin-2-yl)-4,5-dihydrooxazole L3.

A previously reported procedure was used for the synthesis of **L3** from 2-amino-3-methylbutan-1-ol and quinoline-2-carboxylic acid.[11]

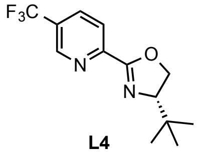

L4

(*S*)-4-(*tert*-butyl)-2-(5-(trifluoromethyl)pyridin-2-yl)-4,5-dihydrooxazole L4.
A previously reported procedure was used for the synthesis of **L4** from (*S*)-tert-leucinol and 5-(trifluoromethyl)pyridine-2-carboxylic acid.[11]

A. General Procedure for the Synthesis of Quinox and Pyrox Ligands – Anderson Coupling[11]

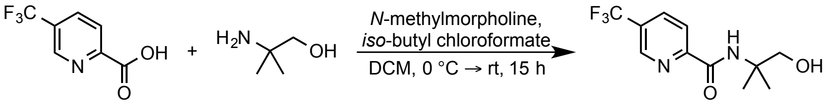

$ $ s1

To an oven dried 100 mL was added 191 mg (1.0 mmol, 1.0 equiv) of 5-(trifluoromethyl)picolinic acid. The flask was placed under an N_2 atmosphere. Dichloromethane (20 mL) was added by syringe, followed by 0.13 mL (1.2 mmol, 1.2 equiv) *N*-methylmorpholine. The reaction mixture was cooled to 0 °C, then 0.16 mL (1.2 mmol, 1.2 equiv) *iso*-butyl chloroformate was added. The mixture was stirred for 20 min, and then 107 mg (1.2 mmol, 1.2 equiv) 2-amino-2-methylpropan-1-ol was added in dichloromethane (15 mL). The mixture was allowed to warm to room temperature and stirred for 15 h. After completion the reaction mixture was transferred to a separatory funnel with dichloromethane (10 mL) and water (10 mL). The aqueous layer was extracted with dichloromethane (1 x 15 mL), and the combined organic layers were washed with water (1 x 20 mL), and brine (1 x 20 mL), then dried over sodium sulfate. The dried organic mixture was concentrated *in vacuo* and purified by silica gel flash chromatography.

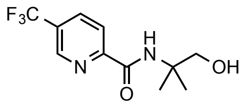

s1

***N*-(1-hydroxy-2-methylpropan-2-yl)-5-(trifluoromethyl)picolinamide s1**.
The general procedure was followed using 1.91 g of 5-(trifluoromethyl)picolinic acid (10 mmol) in dichloromethane (200 mL), 1.27 mL *N*-methylmorpholine (11.5 mmol), 1.57 mL *iso*-butyl chloroformate (12 mmol), and 1.15 mL 2-amino-2-methylpropan-1-ol (12 mmol) in dichloromethane (150 mL). Purification by silica gel flash chromatography (2:1 hexanes:ethyl acetate) afforded **s1** as a white solid (2.37 g, 90%).

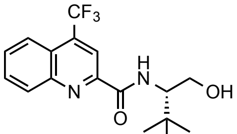

s2

(*S*)-*N*-(1-hydroxy-3,3-dimethylbutan-2-yl)-4-(trifluoromethyl)quinoline-2-carboxamide s2.
The general procedure was followed using 250 mg of 4-(trifluoromethyl)quinolone-2-carboxylic acid (1.04 mmol) in dichloromethane (20 mL), 0.13 mL *N*-methylmorpholine (1.2 mmol), 0.16 mL *iso*-butyl chloroformate (1.24 mmol), and 145 mg (S)-*tert*-leucinol (1.24 mmol) in

dichloromethane (15 mL). Purification by silica gel flash chromatography (2:1 hexanes:ethyl acetate) afforded **s2** as a white solid (290 mg, 82%).

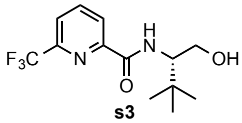

s3

(*S*)-*N*-(1-hydroxy-3,3-dimethylbutan-2-yl)-6-(trifluoromethyl)picolinamide s3.
The general procedure was followed using 956 mg of 6-(trifluoromethyl)picolinic acid (5 mmol) in dichloromethane (100 mL), 0.63 mL *N*-methylmorpholine (5.75 mmol), 0.78 mL *iso*-butyl chloroformate (6 mmol), and 703 mg (S)-*tert*-leucinol (6 mmol) in dichloromethane (75 mL). Purification by silica gel flash chromatography (2:1 hexanes:ethyl acetate) afforded **s3** as a white solid (1.34 g, 92%).

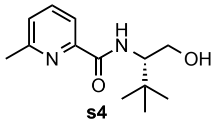

s4

(*S*)-*N*-(1-hydroxy-3,3-dimethylbutan-2-yl)-6-methylpicolinamide s4.
The general procedure was followed using 1.91 g of 6-methylpicolinic acid (13.9 mmol) in dichloromethane, 1.76 mL *N*-methylmorpholine (16 mmol), 2.18 mL *iso*-butyl chloroformate (16.7 mmol), and 1.96 g (S)-*tert*-leucinol (16.7 mmol) in dichloromethane. Purification by silica gel flash chromatography afforded **s4** as a white solid (3.0 g, 91%).

B. General Procedure for the Synthesis of Quinox and Pyrox Ligands – Oxazoline Formation

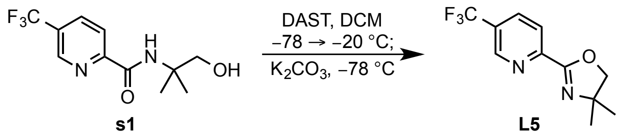

DAST = diethylaminosulfurtrifloride

To an oven dried 50 mL round bottom flask was added 223 mg (0.85 mmol, 1.0 equiv) of **s1** in dichlormethane (12 mL). The flask was placed under an N_2 atmosphere and cooled to –78 °C. To the mixture was added dropwise 0.16 mL (1.19 mmol, 1.4 equiv) of DAST. The mixture was stirred at –78 °C for 1 h before warming to –20 °C and stirring for an additional 1 h. After completion, the mixture was cooled to –78 °C and 236 mg (1.71 mmol, 2.0 equiv) K_2CO_3 was added in one portion. The mixture was warmed to room temperature, diluted with dichloromethane (5 mL) and washed with $NaHCO_3$ (10 mL) and brine (10 mL). The organic phase was dried over sodium sulfate and concentrated *in vacuo*. Crude products were purified by silica gel flash chromatography.

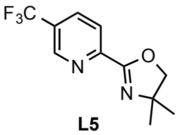

L5

4,4-dimethyl-2-(5-(trifluoromethyl)pyridin-2-yl)-4,5-dihydrooxazole L5.
The general procedure was followed using 1.2 g of **s1** (4.58 mmol) in dichloromethane (61 mL), 0.85 mL DAST (6.41 mmol), and 1.26 g K_2CO_3 (9.16 mmol). Purification by silica gel flash chromatography (5:1 hexanes:acetone) led to the isolation of **L5** as a white solid (834 mg, 75%), mp 90 °C, R_f = 0.36 (5:1 hexanes:acetone). ^{1}H NMR ($CDCl_3$, 500 MHz): δ 8.95 (s, 1H), 8.15 (d, *J* = 8.5 Hz, 1H), 8.02 (dd, *J* = 8.3, 2.3 Hz, 1H), 4.24 (s, 2H), 1.43 (s, 6H); ^{13}C NMR ($CDCl_3$, 126 MHz): δ 160.4, 150.3, 146.9 (q, *J* = 3.8), 134.2 (q, *J* = 3.7), 128.2 (q, *J* = 33.4), 123.7, 123.3 (q, *J*

= 273), 80.1, 68.6, 28.6; FTIR (thin film): 2974, 1640, 1573, 1324, 1089, 1013, 869, 788 cm^{-1}; HRMS *m/z* calculated for $C_{11}H_{11}F_3N_2ONa$ $[M+Na]^+$: 267.0721, found 267.0721.

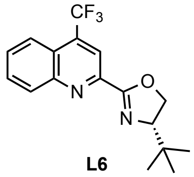

L6

(*S*)-4-(*tert*-butyl)-2-(4-(trifluoromethyl)quinolin-2-yl)-4,5-dihydrooxazole L6.
The general procedure was followed using 290 mg of **s2** (0.85 mmol) in dichloromethane (12 mL), 0.16 mL DAST (1.19 mmol), and 236 mg K_2CO_3 (1.71 mmol). Purification by silica gel flash chromatography (3:1 hexanes:ethyl acetate) led to the isolation of **L6** as a white solid (230 mg, 84%), mp 98 °C, $R_f = 0.42$ (3:1 hexanes:ethyl acetate). $[\alpha]^{20}_D = -94$ (c = 0.284, $CHCl_3$); ^1H NMR ($CDCl_3$, 500 MHz): δ 8.54 (s, 1H), 8.37 (d, *J* = 8.5 Hz, 1H), 8.18 (d, *J* = 8.5 Hz, 1H), 7.86 (t, *J* = 8.0 Hz, 1H), 7.75 (t, *J* = 8.0 Hz, 1H), 4.56 (dd, *J* = 9.0, 10.5 Hz, 1H), 4.43 (t, *J* = 8.5 Hz, 1H), 4.21 (dd, *J* = 8.5, 10.5 Hz, 1H), 1.02 (s, 9H); ^{13}C NMR ($CDCl_3$, 126 MHz): δ 162.2, 148.6, 146.9, 135.2 (q, *J* = 32.3), 131.5, 130.9, 129.8, 124.2 (q, *J* = 1.9), 123.7, 123.5 (q, *J* = 275), 118.5 (q, *J* = 5.4), 77.0, 70.2, 34.3, 26.2; FTIR (thin film): 2957, 1645, 1325, 1253, 1132, 967, 850, 765 cm^{-1}; HRMS *m/z* calculated for $C_{17}H_{17}F_3N_2ONa$ $[M+Na]^+$: 345.1191, found 345.1195.

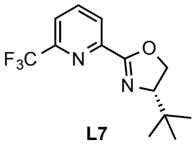

L7

(*S*)-4-(*tert*-butyl)-2-(6-(trifluoromethyl)pyridin-2-yl)-4,5-dihydrooxazole L7.
The general procedure was followed using 726 mg of **s3** (2.5 mmol) in dichloromethane (33 mL), 0.46 mL DAST (3.5 mmol), and 691 mg K_2CO_3 (5.0 mmol). Purification by silica gel flash chromatography (3:1 hexanes:ethyl acetate) led to the isolation of **L7** as a white solid (509 mg, 75%), mp 122 °C, $R_f = 0.29$ (3:1 hexanes:ethyl acetate). $[\alpha]^{20}_D = -89$ (c = 0.334, $CHCl_3$); ^1H NMR ($CDCl_3$, 500 MHz): δ 8.35 (d, *J* = 8.0 Hz, 1H), 7.96 (t, *J* = 8.0 Hz, 1H), 7.78 (dd, *J* = 8.0, 1.0 Hz, 1H), 4.50 (dd, *J* = 8.5, 10.3 Hz, 1H), 4.36 (t, *J* = 8.5 Hz, 1H), 4.13 (dd, *J* = 8.5, 10.5 Hz, 1H), 0.97 (s, 9H); ^{13}C NMR ($CDCl_3$, 126 MHz): δ 161.8, 148.3 (q, *J* = 41), 147.9, 138.2, 127.0, 122.3 (q, *J* = 2.5), 121.4 (q, *J* = 275), 76.6, 70.0, 34.2, 26.1; FTIR (thin film): 2966, 1647, 1363, 1185, 1165, 1077, 955, 836 cm^{-1}; HRMS *m/z* calculated for $C_{13}H_{15}F_3N_2O$ $[M+Na]^+$: 295.1034, found 295.1030.

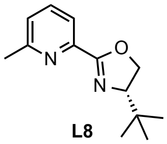

L8

(*S*)-4-(*tert*-butyl)-2-(6-methylpyridin-2-yl)-4,5-dihydrooxazole L8.
The general procedure was followed using 3.0 g of **s4** (12.7 mmol) in dichloromethane, 2.35 mL DAST (17.8 mmol), and 3.51 g K_2CO_3 (25.4 mmol). Purification by silica gel flash chromatography led to the isolation of **L8** as a white solid (2.1 g, 70%), mp 71 °C, $R_f = 0.44$ (2:1 hexanes:acetone). $[\alpha]^{20}_D = -83$ (c = 0.262, $CHCl_3$); ^1H NMR ($CDCl_3$, 500 MHz): δ 7.94 (d, *J* = 8.0 Hz, 1H), 7.64 (t, *J* = 7.5 Hz, 1H), 7.24 (d, *J* = 8.0 Hz, 1H), 4.45 (dd, *J* = 8.5, 10.5 Hz, 1H), 4.31 (t, *J* = 8.5 Hz, 1H), 4.10 (dd, *J* = 8.5, 10.5 Hz, 1H), 2.63 (s, 3H), 0.97 (s, 9H); ^{13}C NMR ($CDCl_3$, 126 MHz): δ 162.9, 158.8, 146.7, 136.9, 125.4, 121.5, 76.6, 69.6, 34.2, 26.2, 24.9; FTIR (thin film): 2951, 1643, 1461, 1360, 1118, 966, 810 cm^{-1}; HRMS *m/z* calculated for $C_{13}H_{18}N_2ONa$ $[M+Na]^+$: 241.1317, found 241.1318.

III. Pd-Catalyzed Three-Component Coupling Reactions

A. General Procedure for Reaction Optimization A

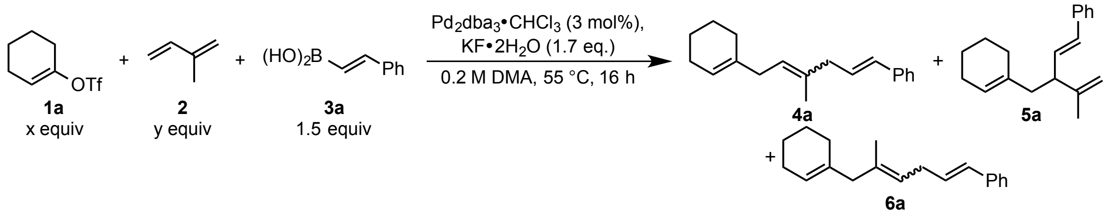

To an oven dried 5 mL vial were added 32 mg (0.34 mmol, 1.7 equiv) of KF•$2H_2O$, 44 mg (0.30 mmol, 1.5 equiv) of **3a**, and 6 mg (3.0 mol%) of Pd_2dba_3•$CHCl_3$. The vial was equipped with a stirbar and the threads were wrapped with Teflon tape, and then was flushed with N_2 before being sealed with a septum cap. To the solids were added a solution of 69 mg (0.3 mmol, 1.5 equiv) of **1a** in 1.0 mL of DMA containing an internal standard (2-methoxynaphthalene) and 20 μL (0.2 mmol, 1.0 equiv) of isoprene. The mixture was stirred for 16 h. After completion, a ~200 μL aliquot of the reaction mixture was removed via syringe and filtered through a silica plug, eluting with ethyl acetate. The mixture was analyzed by GC. Yields were calculated using a response factor (^{1}H NMR spectroscopy was used to measure the response factor to account for varying detector response).

B. Tabular Summary of Reaction Optimization A

Table S1. Optimization of Reaction

entry	solvent	conc. (M)	temp.	base	*x*	*y*	yield (%)	**(*E*)-4a** :	**(*Z*)-4a** :	**5a** :	**(*E*)-6a** :	**(*Z*)-6a**
1	DMA	0.2	55	KF•$2H_2O$	1.5	1.0	17	10	0.64	1.9	1.0	1.1
2	DMA	0.2	55	KF•$2H_2O$	1.0	1.0	21	10	0.64	1.8	1.0	1.1
3	DMA	0.2	55	KF•$2H_2O$	1.0	7.0	61	8.5	0.53	1.7	1.0	1.1
4	DMA	0.2	rt	KF•$2H_2O$	1.0	7.0	61	8.0	0.52	1.5	1.0	1.0
5	DMA	0.2	rt	Na_2CHO_3	1.0	7.0	79	7.6	0.72	1.1	1.0	0.97
6	DMF	0.2	rt	Na_2CO_3	1.0	7.0	81	7.0	0.80	0.73	1.0	0.93
7	DMF	0.25	rt	Na_2CO_3	1.0	7.0	80	7.3	0.85	0.64	1.0	0.89
8[a]	DMF	0.25	rt	Na_2CO_3	1.0	7.0	65	4.5	0.36	1.0	0.05	0.06

[a] Reaction carried out with 8 mol% **L4**.

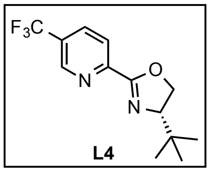

C. Optimized General Procedure A

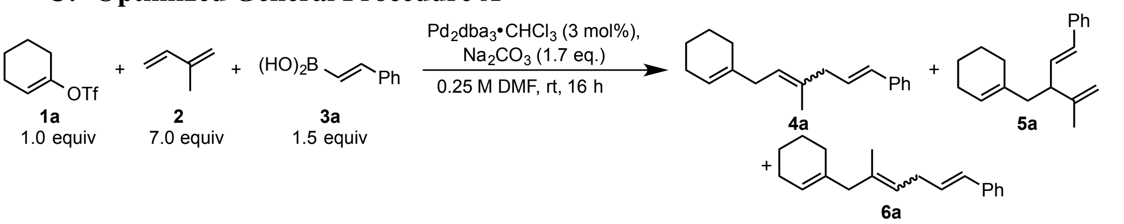

To an oven dried 10 mL round bottom flask were added 90 mg (0.85 mmol, 1.7 equiv) of Na_2CO_3, 111 mg (0.75 mmol, 1.5 equiv) of **3a**, and 16 mg (3.0 mol%) of Pd_2dba_3•$CHCl_3$. The flask was equipped with a PTFE-lined stirbar and a septum, and then was flushed with N_2. To the solids were added a solution of 115 mg (0.5 mmol, 1.0 equiv) of **1a** in 2.0 mL of DMF and 0.35

mL (3.5 mmol, 7.0 equiv) of isoprene. The mixture was stirred for 16 h. After completion, the mixture was diluted with MTBE and filtered through a Celite plug. The organics were washed with H_2O (3 x 10 mL) and brine (1 x 10 mL), then dried over magnesium sulfate and concentrated *in vacuo*. Crude products were purified by silica gel flash chromatography. Yields represent a mixture of stereo and regioisomers. HPLC methods and NMR analysis were used to isolate and verify the identity of product isomers. ^{1}H NMR spectroscopy was used to determine isomeric ratios of the product mixture.

D. General Procedure for Reaction Optimization B

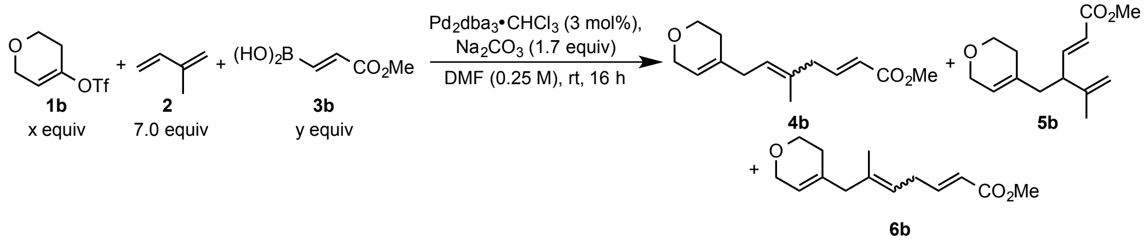

To an oven dried 5 mL vial were added 36 mg (0.34 mmol, 1.7 equiv) of Na_2CO_3, 39 mg (0.3 mmol, 1.5 equiv) of **3b**, and 6 mg (3.0 mol%) of Pd_2dba_3•$CHCl_3$. The vial was equipped with a stirbar and the threads were wrapped with Teflon tape, and then was flushed with N_2 before being sealed with a septum cap. To the solids were added a solution of 46 mg (0.2 mmol, 1.0 equiv) of **1b** in 0.8 mL of DMF and 0.14 mL (1.4 mmol, 7.0 equiv) of isoprene. The mixture was stirred for 16 h. After completion, the mixture was diluted with MTBE and the organics were washed with H_2O (3 x 2 mL) and brine (1 x 2 mL), then dried over magnesium sulfate and concentrated *in vacuo*. ^{1}H NMR spectroscopy was used to determine isomeric ratios and yields using an internal standard (2-methoxynaphthalene).

E. Tabular Summary of Reaction Optimization B

Table S2. Optimization of Reaction

entry	*x*	*y*	yield (%)	**(*E*)-4b**	:	**(*Z*)-4b**	:	**5b**	:	**(*E*)-6b**	:	**(*Z*)-6b**
1	1.0	1.5	17	8.8		3.4		9.0		1.0		1.1
2	1.0	1.2	28	6.6		2.8		3.8		1.0		1.3
3	1.0	1.0	34	6.8		2.8		3.4		1.0		1.0
4	1.2	1.0	50	5.5		0.74		2.1		1.0		0.95
5	1.5	1.0	57	5.3		1.1		1.8		1.0		0.96
6	2.5	1.0	55	6.4		1.7		1.3		1.0		1.8
7	3.0	1.0	73	12		2.4		4.1		1.0		1.1
8	3.5	1.0	69	12		2.7		3.7		1.0		1.5

F. Optimized General Procedure B

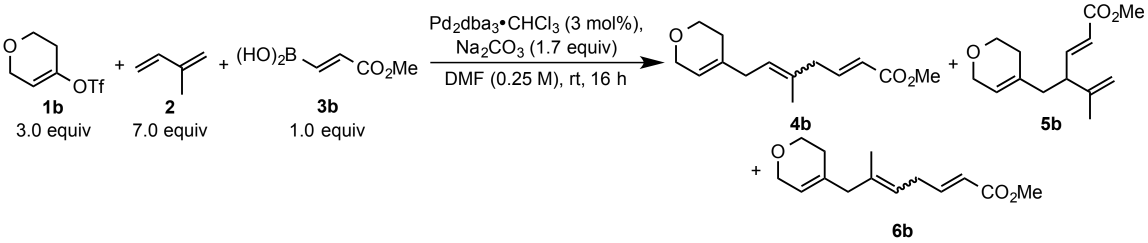

To an oven dried 10 mL round bottom flask were added 90 mg (0.85 mmol, 1.7 equiv) of Na_2CO_3, 65 mg (0.5 mmol, 1.0 equiv) of **3b**, and 16 mg (3.0 mol%) of Pd_2dba_3•$CHCl_3$. The flask

was equipped with a PTFE-lined stirbar and a septum, and then was flushed with N_2. To the solids were added a solution of 348 mg (1.5 mmol, 3.0 equiv) of **1b** in 2.0 mL of DMF and 0.35 mL (3.5 mmol, 7.0 equiv) of isoprene. The mixture was stirred for 16 h. After completion, the mixture was diluted with MTBE and filtered through a Celite plug. The organics were washed with H_2O (3 x 10 mL) and brine (1 x 10 mL), then dried over magnesium sulfate and concentrated *in vacuo*. Crude products were purified by silica gel flash chromatography as noted below. Yields represent a mixture of stereo and regioisomers. HPLC methods and NMR analysis were used to isolate and verify the identity of product isomers. ^{1}H NMR spectroscopy was used to determine isomeric ratios of the product mixture.

G. Scope and Limitations of the 1,4-Difunctionalization Reaction

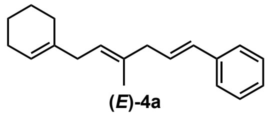

(*E*)-4a

((1*E*,4*E*)-6-(cyclohex-1-en-1-yl)-4-methylhexa-1,4-dien-1-yl)benzene (*E*)-4a.
General procedure A was followed using 90 mg of Na_2CO_3 (0.85 mmol), 111 mg of **3a** (0.75 mmol), 16 mg of Pd_2dba_3•$CHCl_3$ (3 mol%), 115 mg of **1a** (0.5 mmol), and 0.35 mL of isoprene (3.5 mmol) in 2.0 mL of DMF. Purification by flash chromatography (19:1 hexanes:benzene) led to the isolation of **4a**, **5a**, and **6a** as a colorless oil (112 mg, 89% as a 7.3:0.85:0.64:1.0:0.89 mixture of **(*E*)-4a**:**(*Z*)-4a**:**5a**:**(*E*)-6a**:**(*Z*)-6a** isomers respectively), isomeric ratios were determined by GC, R_f = 0.40 (19:1 hexanes:benzene). ^{1}H NMR ($CDCl_3$, 500 MHz): δ 7.35 (d, *J* = 7.5 Hz, 2H), 7.29 (t, *J* = 7.5 Hz, 2H), 7.20 (t, *J* = 7.5 Hz, 1H), 6.40 (d, *J* = 16 Hz, 1H), 6.20 (app. dt, *J* = 7.0, 16 Hz, 1H), 5.41 (m, 1H), 5.26 (t, *J* = 7.5 Hz, 1H), 2.89 (d, *J* = 7.0 Hz, 2H), 2.65 (d, *J* = 7.5 Hz, 2H), 1.98 (m, 2H), 1.92 (m, 2H), 1.62 (m, 5H), 1.55 (m, 2H); (The stereochemistry was confirmed by NOESY1D NMR spectroscopy); The following signals can be assigned to **(Z)-4a**: ^{1}H NMR ($CDCl_3$, 500 MHz): δ 6.14 (app. dt, *J* = 7.0, 16 Hz, 1H), 2.93 (d, *J* = 6.5 Hz, 2H), 1.75 (s, 3H); ^{13}C NMR ($CDCl_3$, 126 MHz): δ 138.0, 137.3, 134.8, 131.2, 129.3, 128.7, 127.1, 126.3, 123.9, 121.1, 43.5, 36.9, 28.8, 25.5, 23.2, 22.8, 16.4; FTIR (thin film): 3025, 2923, 2833, 1495, 1447, 962, 919, 888, 739, 691 cm^{-1}; HRMS *m/z* calculated for $C_{19}H_{24}Ag$ $[M+Ag]^+$: 359.0929, found 359.0947.

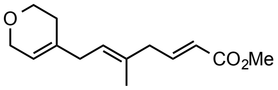

(*E*)-4b

Methyl-(2*E*,5*E*)-7-(3,6-dihydropyran-4-yl)-5-methylhepta-2,5-dienoate (*E*)-4b.
General procedure B was followed using 90 mg of Na_2CO_3 (0.85 mmol), 65 mg of **3b** (0.5 mmol), 16 mg of Pd_2dba_3•$CHCl_3$ (3 mol%), 348 mg of **1b** (1.5 mmol), and 0.35 mL of isoprene (3.5 mmol) in 2.0 mL of DMF. Purification by flash chromatography (3:1 hexanes:EtOAc) led to the isolation of **4b**, **5b**, and **6b** as a colorless oil (63 mg, 53% as a 11:1.2:5.1:1.0:1.0 mixture of **(*E*)-4b**:**(*Z*)-4b**:**5b**:**(*E*)-6b**:**(*Z*)-6b** isomers respectively), R_f = 0.43 (3:1 hexanes:EtOAc). ^{1}H NMR ($CDCl_3$, 500 MHz): δ 6.95 (app. dt, *J* = 7.0, 15.5 Hz, 1H), 5.83 (d, *J* = 15.5 Hz, 1H), 5.41 (m, 1H), 5.24 (t, *J* = 7.0 Hz, 1H), 4.11 (m, 2H), 3.78, (t, *J* = 5.5 Hz, 2H), 3.73 (s, 3H), 2.88 (d, *J* = 7.0 Hz, 2H), 2.70 (d, *J* = 7.0 Hz, 2H), 2.03 (m, 2H), 1.63 (s, 3H); (The stereochemistry was confirmed by NOESY1D NMR spectroscopy); The following signals can be assigned to **(Z)-4b**: ^{1}H NMR ($CDCl_3$, 500 MHz): δ 6.90 (app. dt, *J* = 6.8, 16 Hz, 1H), 5.33 (t, *J* = 7.7 Hz, 1H), 2.91 (d, *J* = 7.1 Hz, 2H), 2.66 (d, *J* = 7.8 Hz, 2H), 1.73 (s, 3H); ^{13}C NMR ($CDCl_3$, 126 MHz): δ 167.2, 147.5, 134.7, 133.7, 124.1, 122.2, 120.1, 65.8, 64.6, 51.7, 42.5, 35.8, 28.9, 16.5; FTIR (thin film): 2951, 1720, 1650, 1434, 1270, 1207, 1165, 1126, 1031, 980, 848 cm^{-1}; HRMS *m/z* calculated for $C_{14}H_{20}O_3Na$ $[M+Na]^+$: 259.1310, found 259.1316.

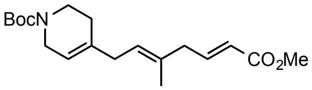

(*E*)-4c

***tert*-Butyl-4-((2*E*,5*E*)-7-methoxy-3-methyl-7-oxohepta-2,5-dien-1-yl)-3,6-dihydropyridine-1-carboxylate (*E*)-4c**.
General procedure B was followed using 90 mg of Na_2CO_3 (0.85 mmol), 65 mg of **3b** (0.5 mmol), 16 mg of Pd_2dba_3•$CHCl_3$ (3 mol%), 497 mg of **1c** (1.5 mmol), and 0.35 mL of isoprene (3.5 mmol) in 2.0 mL of DMF. Purification by flash chromatography (4:1 hexanes:EtOAc) led to the isolation of **4c**, **5c**, and **6c** as a colorless oil (142 mg, 85% as a 6.5:0.42:3.4:1.0:1.1 mixture of **(*E*)-4c**:**(*Z*)-4c**:**5c**:**(*E*)-6c**:**(*Z*)-6c** isomers respectively; 127 mg, 76% as a 7.3:0.48:3.3:1.0:1.1 mixture of **(*E*)-4c**:**(*Z*)-4c**:**5c**:**(*E*)-6c**:**(*Z*)-6c** isomers respectively), R_f = 0.37 (4:1 hexanes:EtOAc). ^{1}H NMR ($CDCl_3$, 500 MHz): δ 6.94 (app. dt, *J* = 7.0, 15.5 Hz, 1H), 5.84 (dt, *J* = 1.5, 16 Hz, 1H), 5.33 (m, 1H), 5.21 (t, *J* = 7.4 Hz, 1H), 3.85 (m, 2H), 3.73 (s, 3H), 3.48 (m, 2H), 2.88 (d, *J* = 7.0 Hz, 2H), 2.69 (d, *J* = 7.0 Hz, 2H), 2.02 (m, 2H), 1.62 (s, 3H), 1.46 (s, 9H); (The stereochemistry was confirmed by NOESY1D NMR spectroscopy); The following signals can be assigned to **(Z)-4c**: ^{1}H NMR ($CDCl_3$, 500 MHz): δ 6.89 (app. dt, *J* = 6.5, 15.5 Hz, 1H), 5.82 (dt, *J* = 1.5, 15.5 Hz, 1H), 2.90 (d, *J* = 7.0 Hz, 2H), 2.66 (d, *J* = 7.5 Hz, 2H), 1.72 (s, 3H); ^{13}C NMR ($CDCl_3$, 126 MHz): δ 167.2, 155.2, 147.4, 133.7, 128.7, 124.2, 122.2, 118.6, 79.7, 51.7, 43.7, 42.5, 40.0, 35.9, 28.7, 16.5; FTIR (thin film): 2977, 1693, 1415, 1367, 1244, 1209, 1164, 1142, 1062, 871, 769, 611 cm^{-1}; HRMS *m*/*z* calculated for $C_{19}H_{29}NO_4Na$ $[M+Na]^+$: 358.1994, found 358.2007.

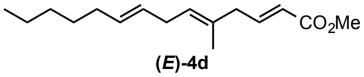

(*E*)-4d

Methyl-(2*E*,5*E*,8*E*)-5-methyltetradeca-2,5,8-trienoate (*E*)-4d.
General procedure B was followed using 90 mg of Na_2CO_3 (0.85 mmol), 65 mg of **3b** (0.5 mmol), 16 mg of Pd_2dba_3•$CHCl_3$ (3 mol%), 369 mg of **1d** (1.5 mmol), and 0.35 mL of isoprene (3.5 mmol) in 2.0 mL of DMF. Purification by flash chromatography (19:1 hexanes:EtOAc) led to the isolation of **4d**, **5d**, and **6d** as a colorless oil (77 mg, 62% as a 5.5:1.4:3.0:1.0:1.4 mixture of **(*E*)-4d**:**(*Z*)-4d**:**5d**:**(*E*)-6d**:**(*Z*)-6d** isomers respectively; 67 mg, 54% as a 6.4:1.4:3.3:1.0:1.4 mixture of **(*E*)-4d**:**(*Z*)-4d**:**5d**:**(*E*)-6d**:**(*Z*)-6d** isomers respectively), **(Z)-4d** and **(Z)-6d** isomeric ratios are reported as a inseparable mixture, R_f = 0.27 (19:1 hexanes:EtOAc). ^{1}H NMR ($CDCl_3$, 500 MHz): δ 6.95 (app. dt, *J* = 7.0, 15.5 Hz, 1H), 5.83 (dt, *J* = 1.5, 15.5 Hz, 1H), 5.42 (app. dt, *J* = 6.5, 15.5 Hz, 1H), 5.35 (app. dt, *J* = 6.0, 15.0 Hz, 1H), 5.22 (t, *J* = 7.0 Hz, 1H), 3.73 (s, 3H), 2.89 (d, *J* = 7.0 Hz, 2H), 2.70 (d, *J* = 7.0 Hz, 2H), 1.97 (q, *J* = 7.0 Hz, 2H), 1.62 (s, 3H), 1.30 (m, 6H), 0.88 (t, *J* = 7.0 Hz, 3H); (The stereochemistry was confirmed by NOESY1D NMR spectroscopy); The following signals can be assigned to **(Z)-4d**: ^{1}H NMR ($CDCl_3$, 500 MHz): δ 2.90 (d, *J* = 6.6 Hz, 2H), 2.67 (t, *J* = 6.6 Hz, 2H), 1.70 (s, 3H); ^{13}C NMR ($CDCl_3$, 126 MHz): δ 167.3, 147.7, 132.2, 131.2, 128.2, 125.8, 122.0, 51.6, 42.5, 32.7, 31.7, 31.5, 29.4, 22.8, 16.4, 14.3; FTIR (thin film): 2925, 2855, 1725, 1643, 1434, 1268, 1161, 1040, 968, 893, 725 cm^{-1}; HRMS *m*/*z* calculated for $C_{16}H_{26}O_2Na$ $[M+Na]^+$: 273.1831, found 273.1824.

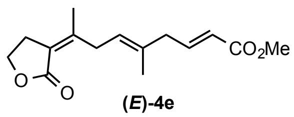

(*E*)-4e

Methyl-(2*E*,5*E*,8*Z*)-5-methyl-8-(2-oxodihydrofuran-3-ylidene)nona-2,5-dienoate (*E*)-4e.
General procedure B was followed using 90 mg of Na_2CO_3 (0.85 mmol), 65 mg of **3b** (0.5 mmol), 16 mg of Pd_2dba_3•$CHCl_3$ (3 mol%), 390 mg of **1e** (1.5 mmol), and 0.35 mL of isoprene (3.5 mmol) in 2.0 mL of DMF. Purification by flash chromatography (3:1 hexanes:EtOAc) led to the isolation of **4e**, **5e**, and **6e** as a colorless oil (110 mg, 83% as a 1.4:0.33:0.30:1.0:0.49 mixture of **(*E*)-4e**:**(*Z*)-4e**:**5e**:**(*E*)-6e**:**(*Z*)-6e** isomers respectively; 116 mg, 87% as a 1.6:0.39:0.48:1.0:0.52 mixture of **(*E*)-4e**:**(*Z*)-4e**:**5e**:**(*E*)-6e**:**(*Z*)-6e** isomers respectively), R_f = 0.32 (3:1 hexanes:EtOAc).

^{1}H NMR ($CDCl_3$, 400 MHz): δ 6.93 (app. dt, J = 6.8, 15.6 Hz, 1H), 5.82 (dt, J = 1.6, 15.6 Hz, 1H), 5.18 (t, J = 7.4 Hz, 1H), 4.30 (t, J = 7.6 Hz, 2H), 3.73 (s, 3H), 3.55 (d, J = 7.2 Hz, 2H), 2.88 (m, 4H), 1.84 (s, 3H), 1.71 (s, 3H); (The stereochemistry was confirmed by NOESY1D NMR spectroscopy); ^{13}C NMR ($CDCl_3$, 126 MHz): δ 170.4, 167.2, 152.6, 147.4, 134.2, 123.5, 122.2, 118.8, 64.4, 51.7, 42.5, 31.5, 28.0, 22.3, 16.8; FTIR (thin film): 2914, 1738, 1716, 1653, 1435, 1269, 1214, 1161, 1031, 987, 847, 754 cm^{-1}; HRMS m/z calculated for $C_{15}H_{20}O_4Na$ $[M+Na]^+$: 287.1259, found 287.1260.

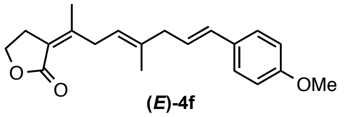

(*E*)-4f

(*Z*)-3-((4*E*,7*E*)-8-(4-methoxyphenyl)-5-methylocta-4,7-dien-2-ylidene)dihydrofuran-2-one (*E*)-4f.

General procedure A was followed using 90 mg of Na_2CO_3 (0.85 mmol), 134 mg of **3c** (0.75 mmol), 16 mg of Pd_2dba_3•$CHCl_3$ (3 mol%), 130 mg of **1e** (0.5 mmol), and 0.35 mL of isoprene (3.5 mmol) in 2.0 mL of DMF. Purification by flash chromatography (3:1 hexanes:EtOAc) led to the isolation of **4f**, **5f**, and **6f** as a colorless oil (126 mg, 81% as a 1.5:0.22:0.44:1.0:0.78 mixture of **(*E*)-4f**:**(*Z*)-4f**:**5f**:**(*E*)-6f**:**(*Z*)-6f** isomers respectively; 136 mg, 84% as a 1.5:0.26:0.47:1.0:0.74 mixture of **(*E*)-4f**:**(*Z*)-4f**:**5f**:**(*E*)-6f**:**(*Z*)-6f** isomers respectively), R_f = 0.30 (3:1 hexanes:EtOAc). ^{1}H NMR ($CDCl_3$, 500 MHz): δ 7.28 (d, J = 8.5 Hz, 2H), 6.83 (d, J = 8.5 Hz, 2H), 6.33 (d, J = 16 Hz, 1H), 6.03 (app. dt, J = 7.0, 16 Hz, 1H), 5.20 (t, J = 7.4 Hz, 1H), 4.29 (t, J = 7.5 Hz, 2H), 3.80 (s, 3H), 3.57 (d, J = 7.0 Hz, 2H), 2.87 (m, 4H), 1.85 (t, J = 2.0 Hz, 3H), 1.73 (s, 3H); (The stereochemistry was confirmed by NOESY1D NMR spectroscopy); The following signals can be assigned to **(*E*)-6f**: ^{1}H NMR ($CDCl_3$, 500 MHz): δ 5.28 (t, J = 7.0 Hz, 1H), 4.36 (t, J = 7.5 Hz, 2H), 1.83 (t, J = 1.5 Hz, 3H), 1.60 (s, 3H); ^{13}C NMR ($CDCl_3$, 126 MHz): δ 170.4, 158.9, 152.1, 134.1, 130.8. 129.4, 127.3, 127.1, 124.6, 120.0, 114.1, 64.3, 55.5, 42.0, 31.7, 28.2, 21.8, 16.0; FTIR (thin film): 2911, 1737, 1606, 1509, 1243, 1172, 1029, 965, 837, 750 cm^{-1}; HRMS m/z calculated for $C_{20}H_{24}O_3Na$ $[M+Na]^+$: 335.1623, found 335.1633.

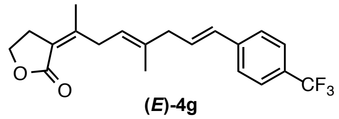

(*E*)-4g

(*Z*)-3-((4*E*,7*E*)-5-methyl-8-(4-(trifluoromethyl)phenyl)octa-4,7-dien-2-ylidene)dihydrofuran-2-one (*E*)-4g.

General procedure A was followed using 90 mg of Na_2CO_3 (0.85 mmol), 162 mg of **3d** (0.75 mmol), 16 mg of Pd_2dba_3•$CHCl_3$ (3 mol%), 130 mg of **1e** (0.5 mmol), and 0.35 mL of isoprene (3.5 mmol) in 2.0 mL of DMF. Purification by flash chromatography (3:1 hexanes:EtOAc) led to the isolation of **4g**, **5g**, and **6g** as a colorless oil (148 mg, 85% as a 1.2:0.24:0.49:1.0:0.55 mixture of **(*E*)-4g**:**(*Z*)-4g**:**5g**:**(*E*)-6g**:**(*Z*)-6g** isomers respectively; 136 mg, 78% as a 1.3:0.27:0.57:1.0:0.56 mixture of **(*E*)-4g**:**(*Z*)-4g**:**5g**:**(*E*)-6g**:**(*Z*)-6g** isomers respectively), R_f = 0.31 (3:1 hexanes:EtOAc). ^{1}H NMR ($CDCl_3$, 500 MHz): δ 7.54 (d, J = 8.2 Hz, 2H), 7.43 (d, J = 8.2 Hz, 2H), 6.42 (d, J = 16 Hz, 1H), 6.29 (app. dt, J = 7.0, 16 Hz, 1H), 5.21 (t, J = 7.5 Hz, 1H), 4.30 (t, J = 7.5 Hz, 2H), 3.57 (d, J = 7.5 Hz, 2H), 2.88 (m, 4H), 1.85 (t, J = 1.5 Hz, 3H), 1.74 (s, 3H); (The stereochemistry was confirmed by NOESY1D NMR spectroscopy); ^{13}C NMR ($CDCl_3$, 126 MHz): δ 170.4, 152.9, 141.3, 135.9, 131.7, 130.2, 129.2, 128.9, 126.4, 125.6 (q, $J_{C\text{-}F}$ = 3.8 Hz), 124.5 (q, $J_{C\text{-}F}$ = 272.2 Hz), 122.4, 118.7, 64.4, 43.4, 31.6, 28.0, 22.3, 16.8; FTIR (thin film): 2918, 1739, 1322, 1161, 1109, 1065, 968, 838, 755 cm^{-1}; HRMS m/z calculated for $C_{20}H_{21}F_3O_2Na$ $[M+Na]^+$: 373.1391, found 373.1395.

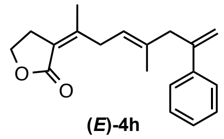

(*E*)-4h

(*Z*)-3-((*E*)-5-methyl-7-phenylocta-4,7-dien-2-ylidene)dihydrofuran-2-one (*E*)-4h.
General procedure A was followed using 90 mg of Na_2CO_3 (0.85 mmol), 111 mg of **3e** (0.75 mmol), 16 mg of Pd_2dba_3•$CHCl_3$ (3 mol%), 130 mg of **1e** (0.5 mmol), and 0.35 mL of isoprene (3.5 mmol) in 2.0 mL of DMF. Purification by flash chromatography (3:1 hexanes:EtOAc) led to the isolation of **4h**, **5h**, and **6h** as a colorless oil (81 mg, 57% as a 1.4:0.20:0.22:1.0:0.42 mixture of **(*E*)-4h**:**(*Z*)-4h**:**5h**:**(*E*)-6h**:**(*Z*)-6h** isomers respectively; 78 mg, 55% as a 1.6:0.20:0.11:1.0:0.42 mixture of **(*E*)-4h**:**(*Z*)-4h**:**5h**:**(*E*)-6h**:**(*Z*)-6h** isomers respectively), R_f = 0.40 (3:1 hexanes:EtOAc). ^1H NMR ($CDCl_3$, 400 MHz): δ 7.38 (m, 2H), 7.27 (m, 3H), 5.37 (s, 1H), 5.17 (m, 1H), 5.07 (s, 1H), 4.27 (t, *J* = 7.6 Hz, 2H), 3.51 (d, *J* = 7.6 Hz, 2H), 3.19 (s, 2H), 2.81 (t, *J* = 7.6 Hz, 2H), 1.66 (s, 3H), 1.62 (s, 3H); (The stereochemistry was confirmed by NOESY1D NMR spectroscopy); The following signals can be assigned to **(*E*)-6h**: ^1H NMR ($CDCl_3$, 400 MHz): δ 5.32 (s, 1H), 5.27 (t, *J* = 7.5 Hz, 1H), 4.32 (m, 2H), 3.54 (s, 2H), 3.21 (d, *J* = 7.5 Hz, 2H), 2.87 (t, *J* = 7.6 Hz, 2H), 1.71 (s, 3H), 1.60 (s, 3H); ^{13}C NMR ($CDCl_3$, 126 MHz): δ 170.4, 153.4, 146.3, 141.4, 135.4, 128.3, 127.4, 126.5, 123.5, 118.3, 114.4, 64.4, 45.9, 31.4, 28.0, 21.9, 16.4; FTIR (thin film): 2912, 1737, 1655, 1443, 1373, 1269, 1187, 1029, 968, 896, 778, 755, 704 cm^{-1}; HRMS *m/z* calculated for $C_{19}H_{22}O_2Na$ $[M+Na]^+$: 305.1526, found 305.1517.

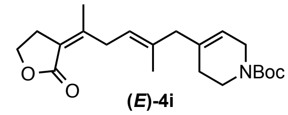

(*E*)-4i

***tert*-butyl-4-((2*E*,5*Z*)-2-methyl-5-(2-oxodihydrofuran-3-ylidene)hex-2-en-1-yl)-3,6-dihydropyridine-1-carboxylate (*E*)-4i**.
General procedure A was followed using 90 mg of Na_2CO_3 (0.85 mmol), 170 mg of **3f** (0.75 mmol), 16 mg of Pd_2dba_3•$CHCl_3$ (3 mol%), 130 mg of **1e** (0.5 mmol), and 0.35 mL of isoprene (3.5 mmol) in 2.0 mL of DMF. Purification by flash chromatography (3:1 hexanes:EtOAc) led to the isolation of **4i**, **5i**, and **6i** as a colorless oil (148 mg, 81% as a 1.5:0.09:0.14:1.0:0.60 mixture of **(*E*)-4i**:**(*Z*)-4i**:**5i**:**(*E*)-6i**:**(*Z*)-6i** isomers respectively; 162 mg, 90% as a 1.5:0.21:0.13:1.0:0.61 mixture of **(*E*)-4i**:**(*Z*)-4i**:**5i**:**(*E*)-6i**:**(*Z*)-6i** isomers respectively), R_f = 0.32 (3:1 hexanes:EtOAc). ^1H NMR ($CDCl_3$, 500 MHz): δ 5.37 (m, 1H), 5.15 (t, *J* = 7.0 Hz, 1H), 4.30 (t, *J* = 7.5 Hz, 2H), 3.86 (m, 2H), 3.55 (d, *J* = 7.0 Hz, 2H), 3.45 (m, 2H), 2.88 (t, *J* = 7.8 Hz, 2H), 2.67 (s, 2H), 1.93 (m, 2H), 1.84 (t, *J* = 2.0 Hz, 3H), 1.61 (s, 3H), 1.46 (s, 9H); (The stereochemistry was confirmed by NOESY1D NMR spectroscopy); The following signals can be assigned to **(*E*)-6i**: ^1H NMR ($CDCl_3$, 500 MHz): δ 5.19 (t, *J* = 6.5 Hz, 1H), 2.91 (t, *J* = 8.0 Hz, 2H), 2.70 (d, *J* = 7.5 Hz, 2H), 1.80 (t, *J* = 1.5 Hz, 3H); ^{13}C NMR ($CDCl_3$, 126 MHz): δ 170.5, 155.3, 153.1, 135.2, 130.0, 123.1, 119.6, 118.5, 79.7, 64.4, 48.1, 43.8, 40.0, 31.6, 28.7, 28.0, 22.2, 16.1; FTIR (thin film): 2913, 1742, 1691, 1415, 1364, 1238, 1166, 1036, 966, 864, 755 cm^{-1}; HRMS *m/z* calculated for $C_{21}H_{31}NO_4Na$ $[M+Na]^+$: 384.2151, found 384.2148.

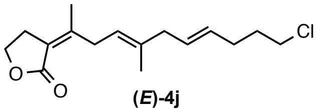

(*E*)-4j

(*Z*)-3-((4*E*,7*E*)-11-chloro-5-methylundeca-4,7-dien-2-ylidene)dihydrofuran-2-one (*E*)-4j.
General procedure A was followed using 90 mg of Na_2CO_3 (0.85 mmol), 111 mg of **3g** (0.75 mmol), 16 mg of Pd_2dba_3•$CHCl_3$ (3 mol%), 130 mg of **1e** (0.5 mmol), and 0.35 mL of isoprene (3.5 mmol) in 2.0 mL of DMF. Purification by flash chromatography (3:1 hexanes:EtOAc) led to the isolation of **4j**, **5j**, and **6j** as a colorless oil (64 mg, 46% as a 1.1:0.33:0.38:1.0:0.87 mixture of **(*E*)-4j**:**(*Z*)-4j**:**5j**:**(*E*)-6j**:**(*Z*)-6j** isomers respectively; 76 mg, 54% as a 1.0:0.33:0.30:1.0:0.81 mixture of **(*E*)-4j**:**(*Z*)-4j**:**5j**:**(*E*)-6j**:**(*Z*)-6j** isomers respectively), R_f = 0.40 (3:1 hexanes:EtOAc).

^{1}H NMR ($CDCl_3$, 500 MHz): δ 5.41 (app. dt, J = 6.5, 16 Hz, 2H), 5.12 (t, J = 7.2 Hz, 1H), 4.29 (t, J = 7.5 Hz, 2H), 3.53 (m, 4H), 2.87 (t, J = 7.0 Hz, 2H), 2.67 (d, J = 6.5 Hz, 2H), 2.16 (q, J = 7.0 Hz, 2H), 1.82 (m, 5H), 1.67 (s, 3H); (The stereochemistry was confirmed by NOESY1D NMR spectroscopy); The following signals can be assigned to **(*E*)-6j**: ^{1}H NMR ($CDCl_3$, 500 MHz): δ 5.19 (t, J = 6.0 Hz, 1H), 2.92 (t, J = 7.7 Hz, 2H), 2.71 (d, J = 6.8 Hz, 2H), 1.80 (m, 3H), 1.54 (s, 3H); ^{13}C NMR ($CDCl_3$, 126 MHz): δ 170.4, 153.3, 136.8, 130.0, 121.3, 121.1, 118.4, 64.4, 44.6, 43.1, 32.4, 31.5, 29.8, 28.0, 22.2, 16.6; FTIR (thin film): 2913, 1738, 1657, 1441, 1373, 1162, 1035, 967, 753, 646 cm^{-1}; HRMS *m/z* calculated for $C_{16}H_{23}ClO_2Na$ $[M+Na]^+$: 305.1284, found 305.1286.

H. General Procedure for 1,4-Difunctionalization Ligand Screens

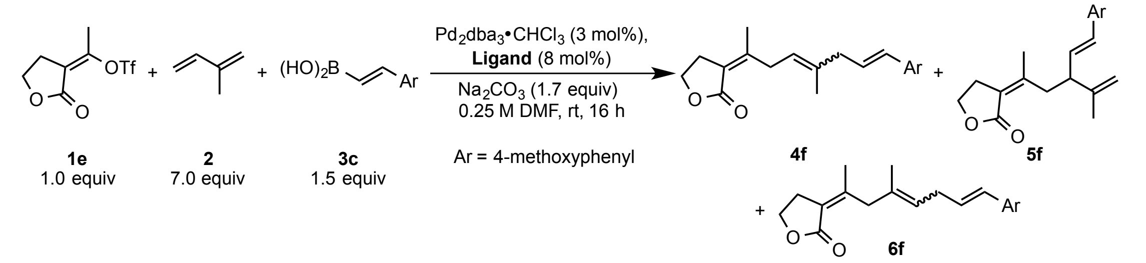

To an oven dried 5 mL vial were added 36 mg (0.34 mmol, 1.7 equiv) of Na_2CO_3, 52 mg (0.2 mmol, 1.0 equiv) of **1e,** 53 mg (0.3 mmol, 1.5 equiv) of **3c**, 2 mg (8 mol%) **L1,** and 6 mg (3.0 mol%) of Pd_2dba_3•$CHCl_3$. The vial was equipped with a stirbar and the threads were wrapped with Teflon tape, and then was flushed with N_2 before being sealed with a septum cap. To the solids were added 0.8 mL of DMF and 0.14 mL (1.4 mmol, 7.0 equiv) of isoprene. The mixture was stirred for 16 h. After completion, the mixture was diluted with MTBE and the organics were washed with H_2O (3 x 2 mL) and brine (1 x 2 mL), then dried over magnesium sulfate and concentrated *in vacuo*. ^{1}H NMR spectroscopy was used to determine isomeric ratios and yields using an internal standard (2-methoxynaphthalene). Alkene insertion selectivity is defined as the ratio of **4f** + **5f** to **(*E*)-6f**.

I. Tabular Summary of 1,4-Difunctionalization Ligand Screen

Table S3.

Ligand	yield	**(*E*)-4f**	:	**5f**	:	**(*E*)-6f**	Suzuki
**none**	82%	1.5		0.45		1.0	16%
**L1**	77%	1.3		0.36		1.0	15%
**L2**	83%	1.6		0.43		1.0	19%
**L3**	25%	2.1		1.0		1.0	30%
**L4**	62%	7.7		2.4		1.0	13%
**L5**	35%	3.3		2.6		1.0	36%
**L6**	67%	1.3		0.67		1.0	19%
**L7**	75%	1.3		0.77		1.0	16%
**L8**	47%	1.7		1.9		1.0	35%

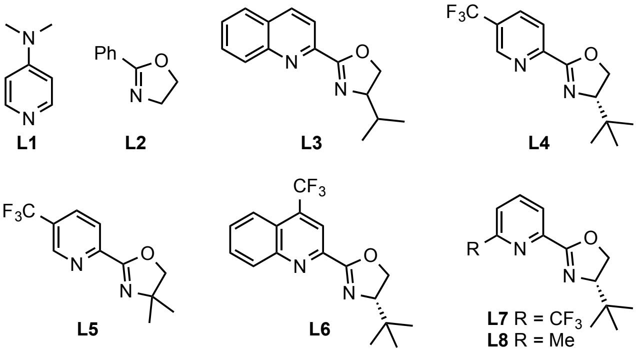

J. Training Set Data for Pyrox Ligand Library

Table S4.

entry	R	X	pred. $\Delta\Delta G^{\ddagger}$ (kcal/mol)	meas. $\Delta\Delta G^{\ddagger}$ (kcal/mol)	meas. alkene insertion selectivty
1	H	4-H	0.74	0.79	3.81
2	H	4-Cl	0.72	0.66	3.06
3	H	5-CF_3	0.70	0.76	3.58
4	Me	5-CF_3	0.99	0.94	4.91
5	Ph	5-CF_3	1.09	1.13	6.72
6	Bn	5-CF_3	1.28	1.31	9.19
7	*i*-Pr	4-H	1.23	1.05	5.91
8	*i*-Pr	4-Cl	1.21	1.27	8.59
9	*i*-Pr	5-CF_3	1.19	1.17	7.19
10	*t*-Bu	4-H	1.48	1.46	11.8
11	*t*-Bu	4-Cl	1.46	1.68	17.0
12	*t*-Bu	5-CF_3	1.44	1.59	14.6
13	*t*-Bu	4-Me	1.49	1.44	11.3
14	*t*-Bu	5-CN	1.42	1.36	9.98
15	*t*-Bu	5-NO_2	1.41	1.25	8.29

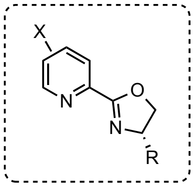

K. General Procedure of Reaction Optimization C

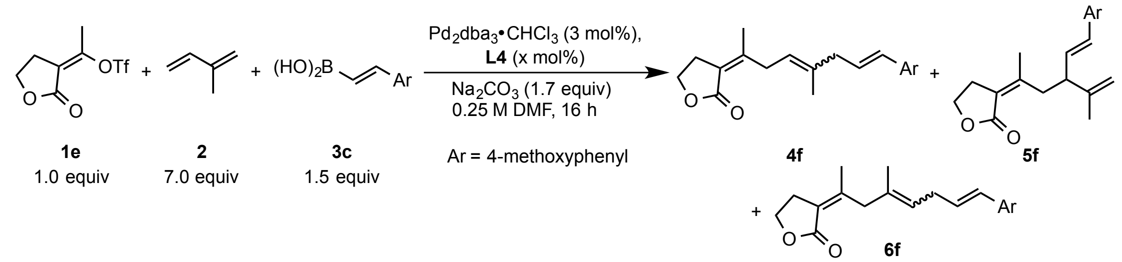

To an oven dried 5 mL vial were added 36 mg (0.34 mmol, 1.7 equiv) of Na_2CO_3, 52 mg (0.2 mmol, 1.0 equiv) of **1e**, 53 mg (0.3 mmol, 1.5 equiv) of **3c**, 4 mg (8 mol%) **L4**, and 6 mg (3.0 mol%) of Pd_2dba_3•$CHCl_3$. The vial was equipped with a stirbar and the threads were wrapped with Teflon tape, and then was flushed with N_2 before being sealed with a septum cap. To the solids were added 0.8 mL of DMF and 0.14 mL (1.4 mmol, 7.0 equiv) of isoprene. The mixture was stirred for 16 h. After completion, the mixture was diluted with MTBE and the organics were washed with H_2O (3 x 2 mL) and brine (1 x 2 mL), then dried over magnesium sulfate and concentrated *in vacuo*. ^{1}H NMR spectroscopy was used to determine isomeric ratios and yields using an internal standard (2-methoxynaphthalene).

L. Tabular Summary of Reaction Optimization C

Table S5.

x (mol%)	temp.	yield (%)	**(*E*)-4f**	:	**5f**	:	**(*E*)-6f**	Suzuki (%)
8	rt	52	8.0		4.0		1.0	16
7	rt	59	7.7		3.6		1.0	15
7	45 °C	66	6.8		3.1		1.0	11

M. Optimized General Procedure C

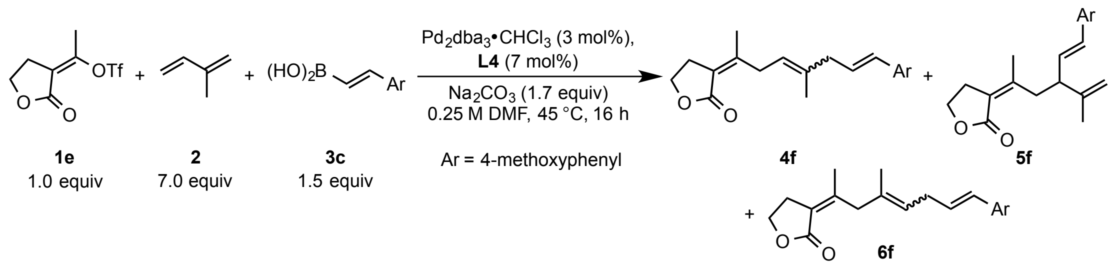

To an oven dried 10 mL round bottom flask were added 90 mg (0.85 mmol, 1.7 equiv) of Na_2CO_3, 130 mg (0.5 mmol, 1.0 equiv) of **1e**, 134 mg (0.75 mmol, 1.5 equiv) of **3c**, 10 mg (7.0 mol%) of **L4**, and 16 mg (3.0 mol%) of Pd_2dba_3•$CHCl_3$. The flask was equipped with a PTFE-lined stirbar and a septum, and then was flushed with N_2. To the solids were added a solution of in 2.0 mL of DMF and 0.35 mL (3.5 mmol, 7.0 equiv) of isoprene. The mixture was stirred for 16 h at 45 °C. After completion, the mixture was diluted with MTBE and filtered through a Celite plug. The organics were washed with H_2O (3 x 10 mL) and brine (1 x 10 mL), then dried over magnesium sulfate and concentrated *in vacuo*. Crude products were purified by silica gel flash chromatography. Yields represent a mixture of stereo and regioisomers. HPLC methods and NMR analysis were used to isolate and verify the identity of product isomers. ^{1}H NMR spectroscopy was used to determine isomeric ratios of the product mixture.

N. Scope and Limitations of the 1,4-Difunctionalization Reaction with Ligand

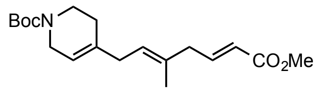

(*E*)-4c

***tert*-Butyl-4-((2*E*,5*E*)-7-methoxy-3-methyl-7-oxohepta-2,5-dien-1-yl)-3,6-dihydropyridine-1-carboxylate (*E*)-4c**.

General procedure C was followed using 90 mg of Na_2CO_3 (0.85 mmol), 65 mg of **3b** (0.5 mmol), 10 mg of **L4** (7.0 mol%), 16 mg of Pd_2dba_3•$CHCl_3$ (3 mol%), 497 mg of **1c** (1.5 mmol), and 0.35 mL of isoprene (3.5 mmol) in 2.0 mL of DMF. Purification by flash chromatography (4:1 hexanes:EtOAc) led to the isolation of **4c**, **5c**, and **6c** as a colorless oil (84 mg, 50% as a 9.2:1.3:5.9:1.0:0.79 mixture of **(*E*)-4c**:**(Z)-4c**:**5c**:**(*E*)-6c**:**(Z)-6c** isomers respectively; 95 mg, 57% as a 8.6:1.3:5.2:1.0:1.0 mixture of **(*E*)-4c**:**(Z)-4c**:**5c**:**(*E*)-6c**:**(Z)-6c** isomers respectively).

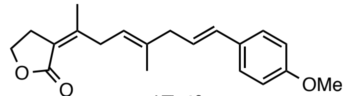

(*E*)-4f

(*Z*)-3-((4*E*,7*E*)-8-(4-methoxyphenyl)-5-methylocta-4,7-dien-2-ylidene)dihydrofuran-2-one (*E*)-4f.

General procedure A was followed using 90 mg of Na_2CO_3 (0.85 mmol), 134 mg of **3c** (0.75 mmol), 16 mg of Pd_2dba_3•$CHCl_3$ (3 mol%), 130 mg of **1e** (0.5 mmol), and 0.35 mL of isoprene (3.5 mmol) in 2.0 mL of DMF. Purification by flash chromatography (3:1 hexanes:EtOAc) led to the isolation of **4f**, **5f**, and **6f** as a colorless oil (120 mg, 77% as a 7.2:1.0:2.7:1.0:0.75 mixture of **(*E*)-4f**:**(*Z*)-4f**:**5f**:**(*E*)-6f**:**(*Z*)-6f** isomers respectively).

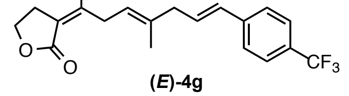

(*E*)-4g

(*Z*)-3-((4*E*,7*E*)-5-methyl-8-(4-(trifluoromethyl)phenyl)octa-4,7-dien-2-ylidene)-dihydrofuran-2-one (*E*)-4g.

General procedure A was followed using 90 mg of Na_2CO_3 (0.85 mmol), 162 mg of **3d** (0.75 mmol), 16 mg of Pd_2dba_3•$CHCl_3$ (3 mol%), 130 mg of **1e** (0.5 mmol), and 0.35 mL of isoprene

(3.5 mmol) in 2.0 mL of DMF. Purification by flash chromatography (3:1 hexanes:EtOAc) led to the isolation of **4g**, **5g**, and **6g** as a colorless oil (142 mg, 81% as a 6.6:0.97:4.1:1.0:0.60 mixture of **(*E*)-4g**:**(Z)-4g**:**5g**:**(*E*)-6g**:**(Z)-6g** isomers respectively).

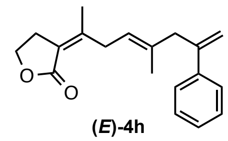

(*E*)-4h

(*Z*)-3-((*E*)-5-methyl-7-phenylocta-4,7-dien-2-ylidene)dihydrofuran-2(3*H*)-one (*E*)-4h.
General procedure A was followed using 90 mg of Na_2CO_3 (0.85 mmol), 111 mg of **3e** (0.75 mmol), 16 mg of Pd_2dba_3•$CHCl_3$ (3 mol%), 130 mg of **1e** (0.5 mmol), and 0.35 mL of isoprene (3.5 mmol) in 2.0 mL of DMF. Purification by flash chromatography (3:1 hexanes:EtOAc) led to the isolation of **4h**, **5h**, and **6h** as a colorless oil (62 mg, 44% as a 4.2:0.92:1.1:1.0:0.43 mixture of **(*E*)-4h**:**(*Z*)-4h**:**5h**:**(*E*)-6h**:**(Z)-6h** isomers respectively; 69 mg, 49% as a 4.3:0.87:0.97:1.0:0.36 mixture of **(*E*)-4h**:**(*Z*)-4h**:**5h**:**(*E*)-6h**:**(Z)-6h** isomers respectively).

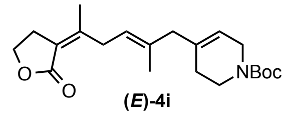

(*E*)-4i

***tert*-butyl-4-((2*E*,5*Z*)-2-methyl-5-(2-oxodihydrofuran-3-ylidene)hex-2-en-1-yl)-3,6-dihydropyridine-1-carboxylate (*E*)-4i**.
General procedure A was followed using 90 mg of Na_2CO_3 (0.85 mmol), 170 mg of **3f** (0.75 mmol), 16 mg of Pd_2dba_3•$CHCl_3$ (3 mol%), 130 mg of **1e** (0.5 mmol), and 0.35 mL of isoprene (3.5 mmol) in 2.0 mL of DMF. Purification by flash chromatography (3:1 hexanes:EtOAc) led to the isolation of **4i**, **5i**, and **6i** as a colorless oil (126 mg, 70% as a 9.0:1.1:3.3:1.0:0.67 mixture of **(*E*)-4i**:**(Z)-4i**:**5i**:**(*E*)-6i**:**(Z)-6i** isomers respectively).

IV. References

1. Zalesskiy, S. S.; Ananikov, V. P. *Organometallics* 2012, **31**, 2302.
2. Tessier, P. E.; Nguyen, N.; Clay, M. D.; Fallis, A. G. *Org. Lett.* 2005, **7**, 767.
3. Patel, M. V.; Kolasa, T.; Mortell, K.; Matulenko, M. A.; Hakeem, A. A.; Rohde, J. J.; Nelson, S. L.; Cowart, M. D.; Nakane, M.; Miller, L. N.; Uchic, M. E.; Terranova, M. A.; El-Kouhen, O. F.; Donnelly-Roberts, D. L.; Namovic, M. T.; Hollingsworth, P. R.; Chang, R.; Martino, B. R.; Wetter, J. M.; Marsh, K. C.; Martin, R.; Darbyshire, J. F.; Gintant, G.; Hsieh, G. C.; Moreland, R. B.; Sullivan, J. P.; Brioni, J. D.; Stewart, A. O. *J. Med. Chem.* 2006, **49**, 7450.
4. McCammant, M. S.; Liao, L.; Sigman, M. S. *J. Am. Chem. Soc.* 2013, **135**, 4167.
5. Matsuzawa, S.; Horiguchi, Y.; Nakamura, E.; Kuwajima, I. *Tetrahedron* 1989, **45**, 349.
6. Mi, Y.; Schreiber, J. V.; Corey, E. J. *J. Am. Chem. Soc.* 2002, **124**, 11290.
7. Pouwer, R. H.; Schill, H.; Williams, C. M.; Bernhardt, P. V. *Eur. J. Org. Chem.* 2007, **2007**, 4699.
8. Gravel, M.; Touré, B. B.; Hall, D. G. *Org. Prep. Proced. Int.* 2004, **36**, 573.
9. Castro, A. C.; Evans, C. A.; Grenier, L.; Grogan, M. J. Preparation of piperideneboronic acid derivatives for use as fatty acid amide hydrolase inhibitors. WO2010118159, 2010.
10. Suero, M. G.; Bayle, E. D.; Collins, B. S. L.; Gaunt, M. J. *J. Am. Chem. Soc.* 2013, **135**, 5332.
11. Werner, E. W.; Mei, T.-S.; Burckle, A. J.; Sigman, M. S. *Science* 2012, **338**, 1455.

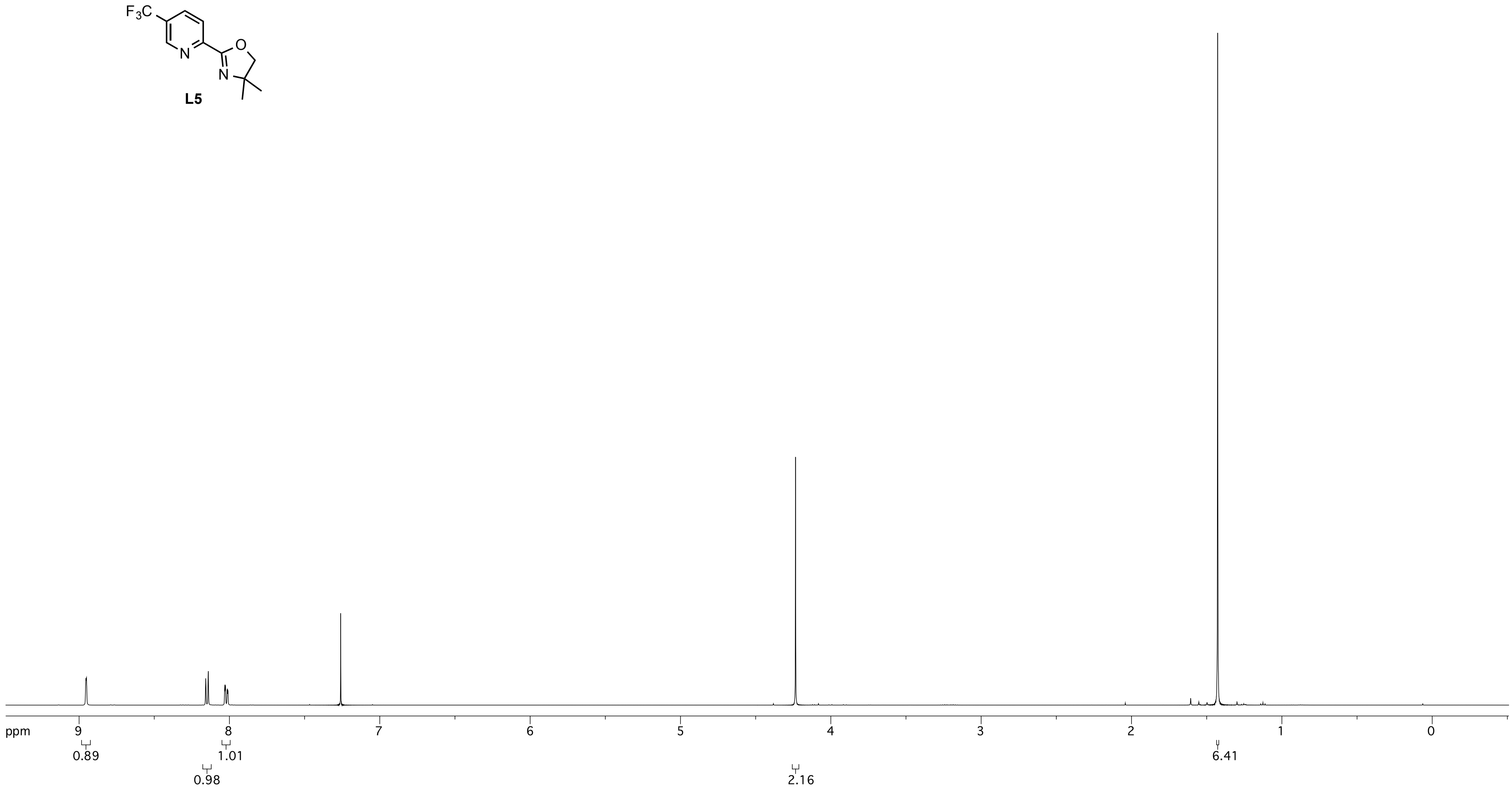

F3C
N
O
N
L5
ppm
9
8
7
6
5
4
3
2
1
0
0.89
1.01
0.98
2.16
6.41

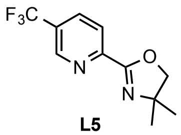

L5

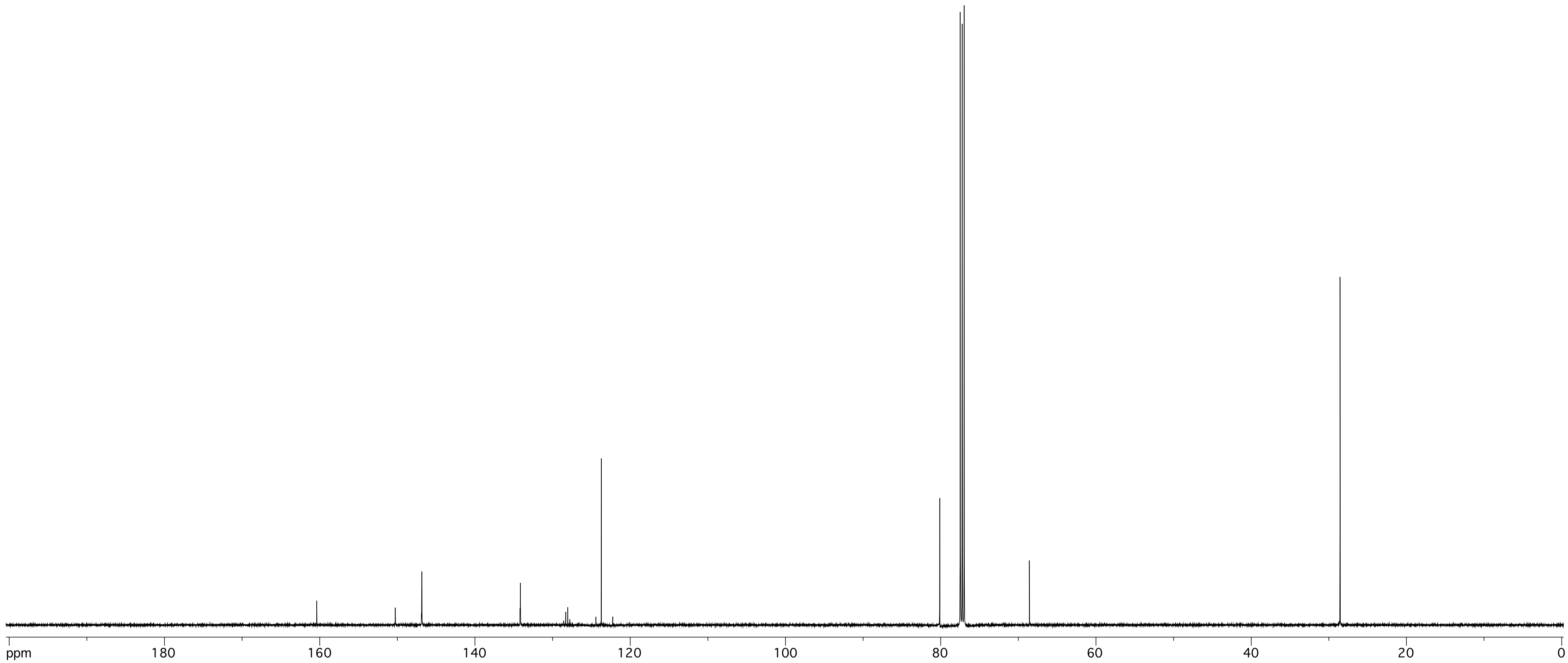

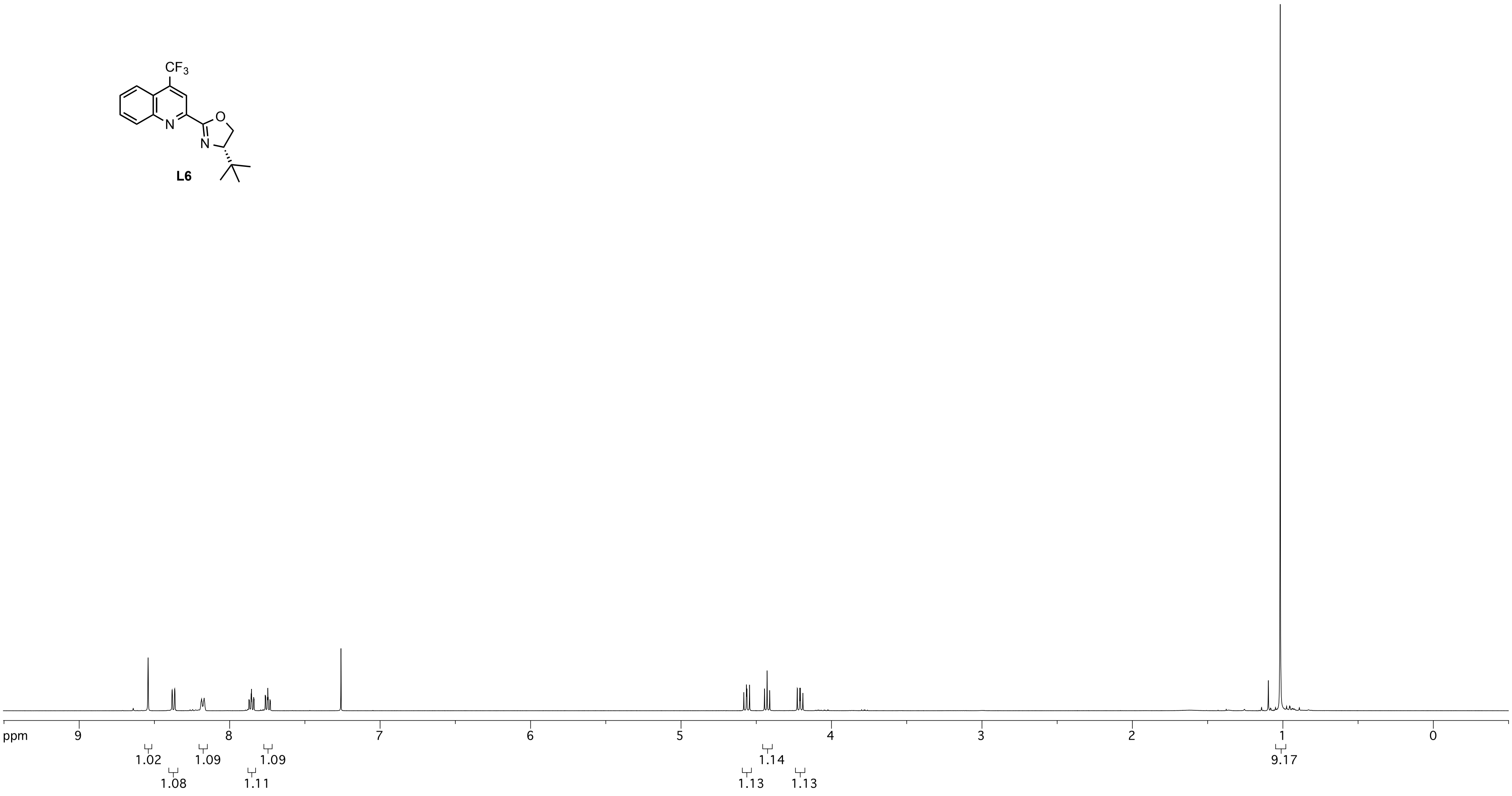
CF3
N
O
N
L6
ppm
9
8
7
6
5
4
3
2
1
0
1.02
1.08
1.09
1.11
1.09
1.13
1.14
1.13
9.17

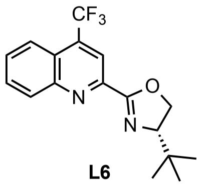

L6

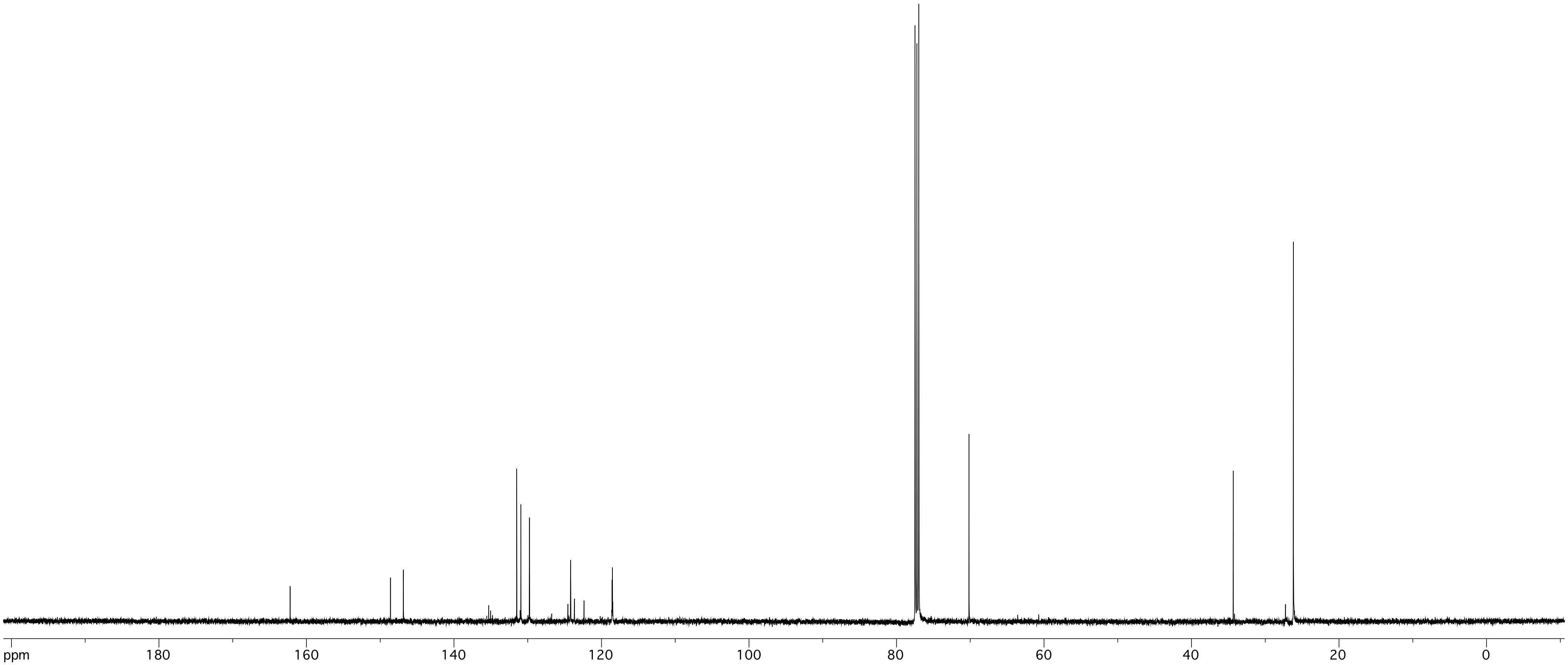

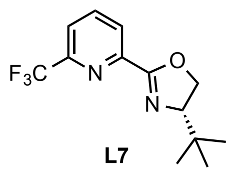

L7

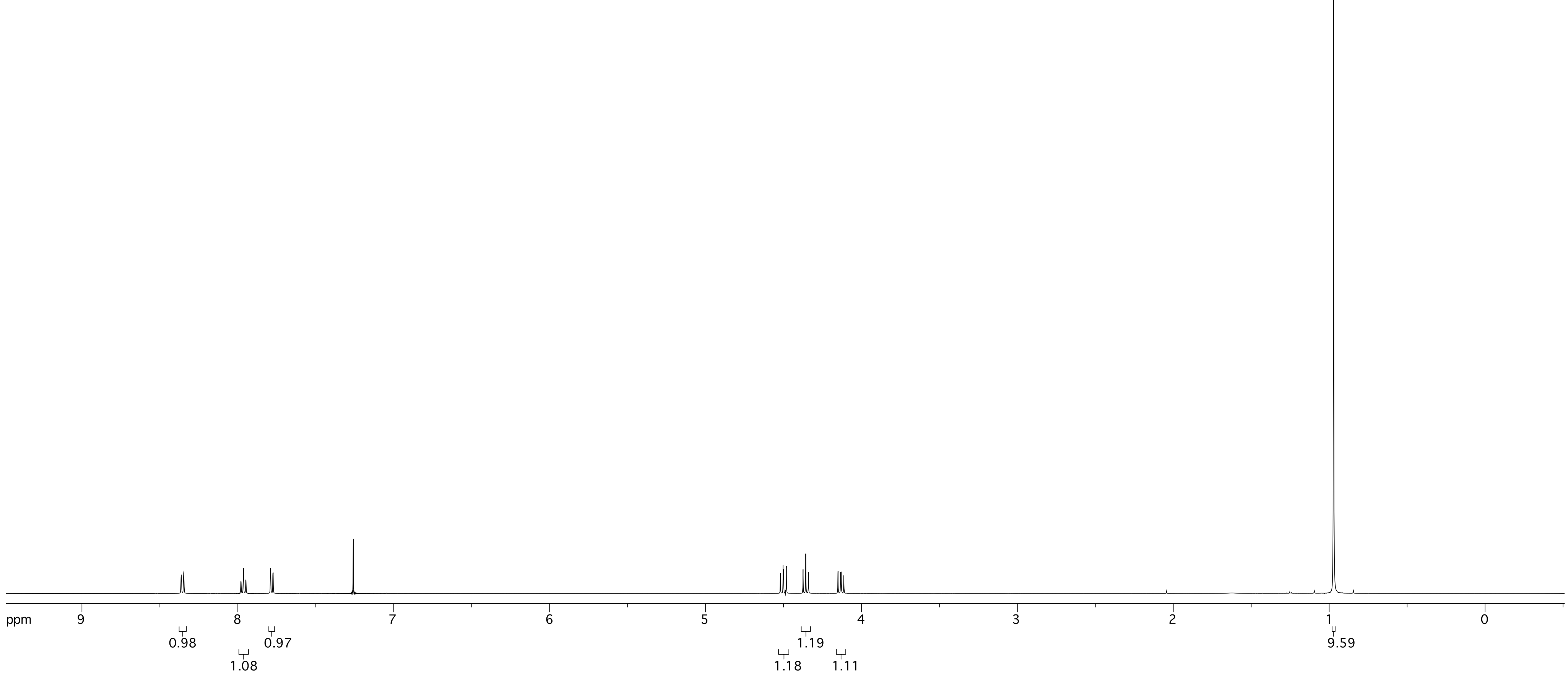

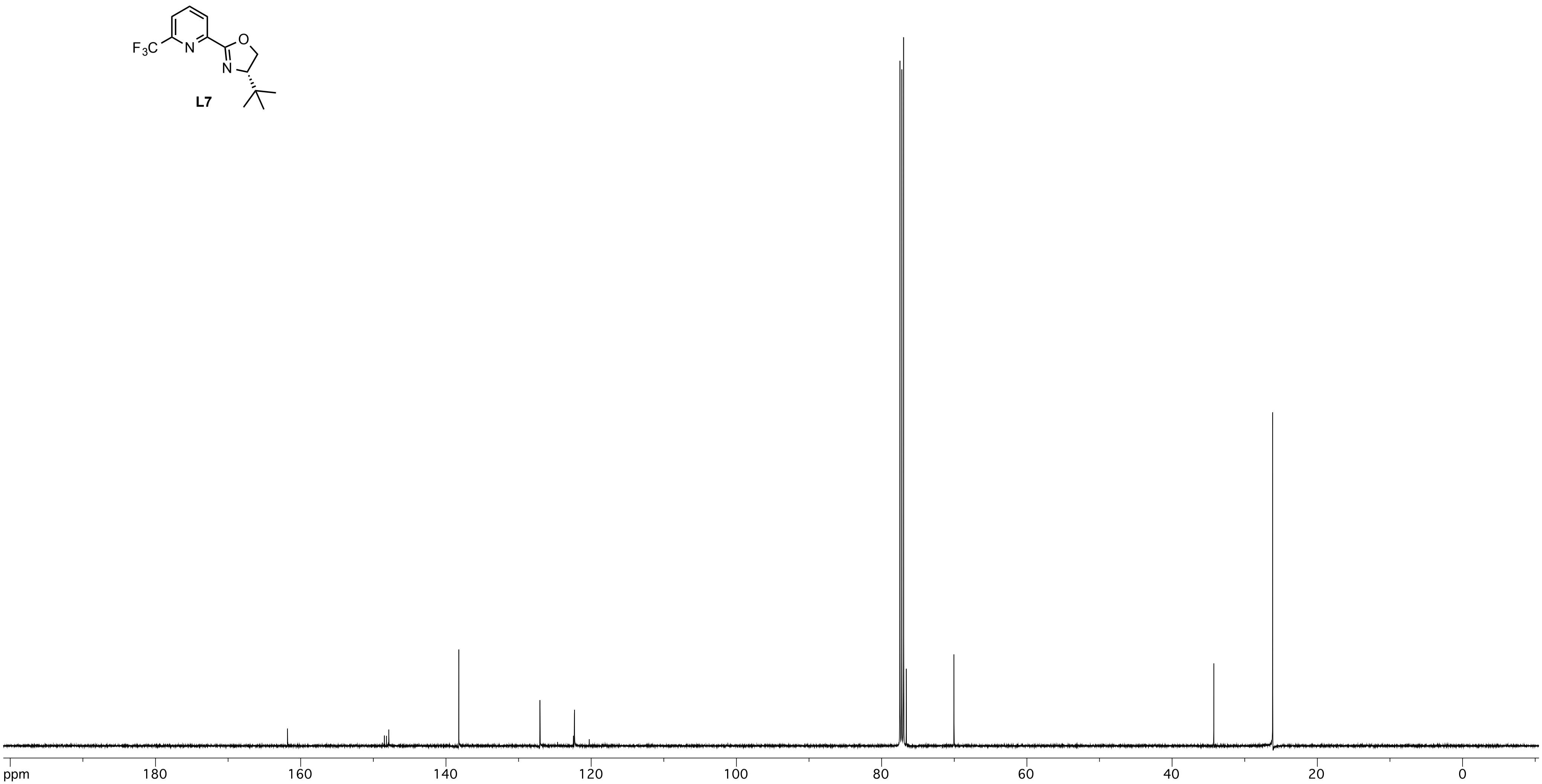

L7

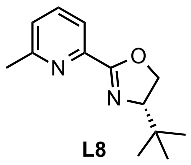

L8

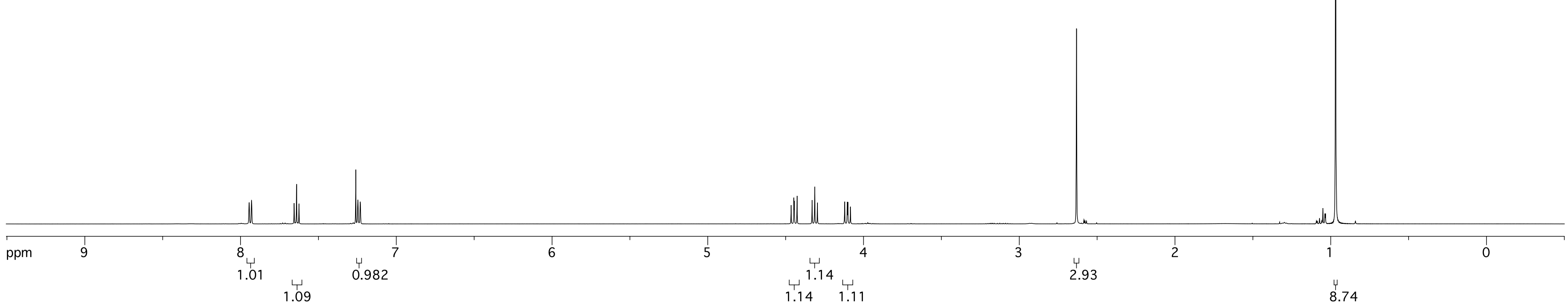

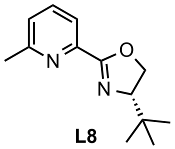

L8

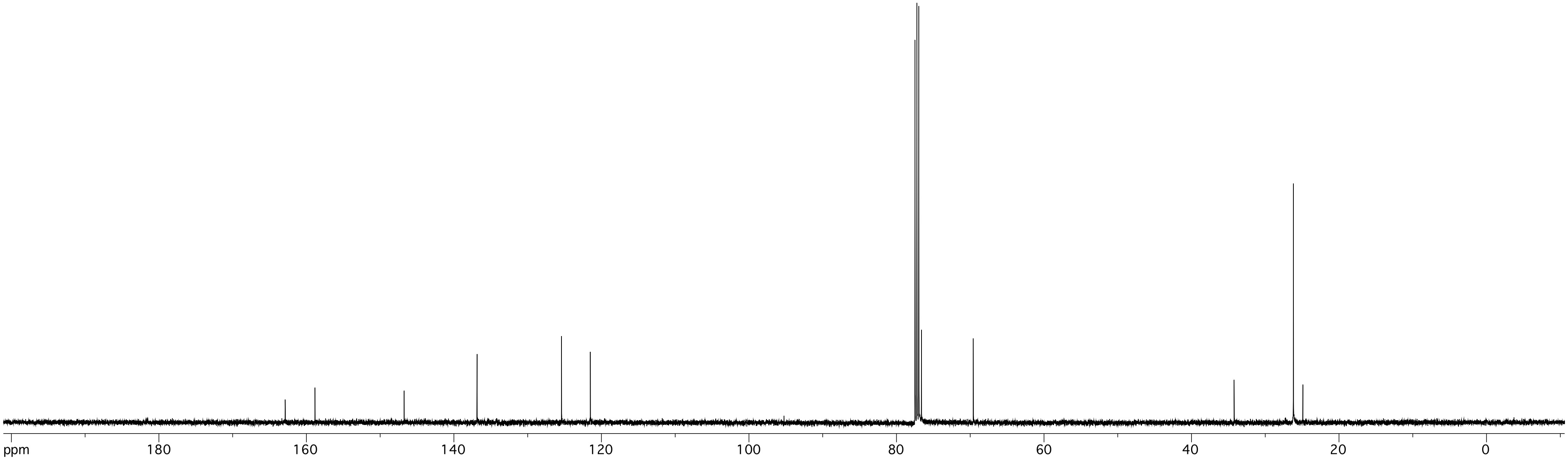

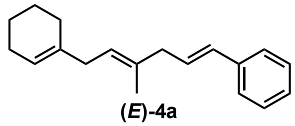
(*E*)-4a

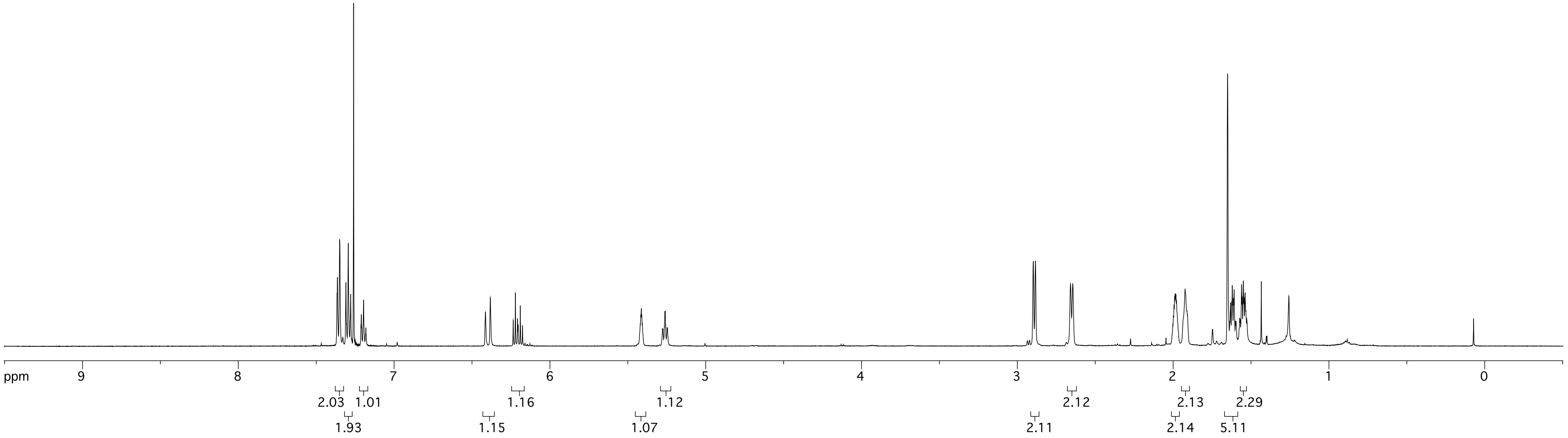

ppm
9
8
7
6
5
4
3
2
1
0
2.03
1.01
1.93
1.16
1.15
1.12
1.07
2.12
2.11
2.13
2.29
2.14
5.11

NOESY1D

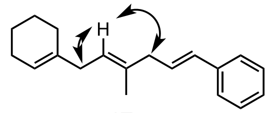

(*E*)-4a

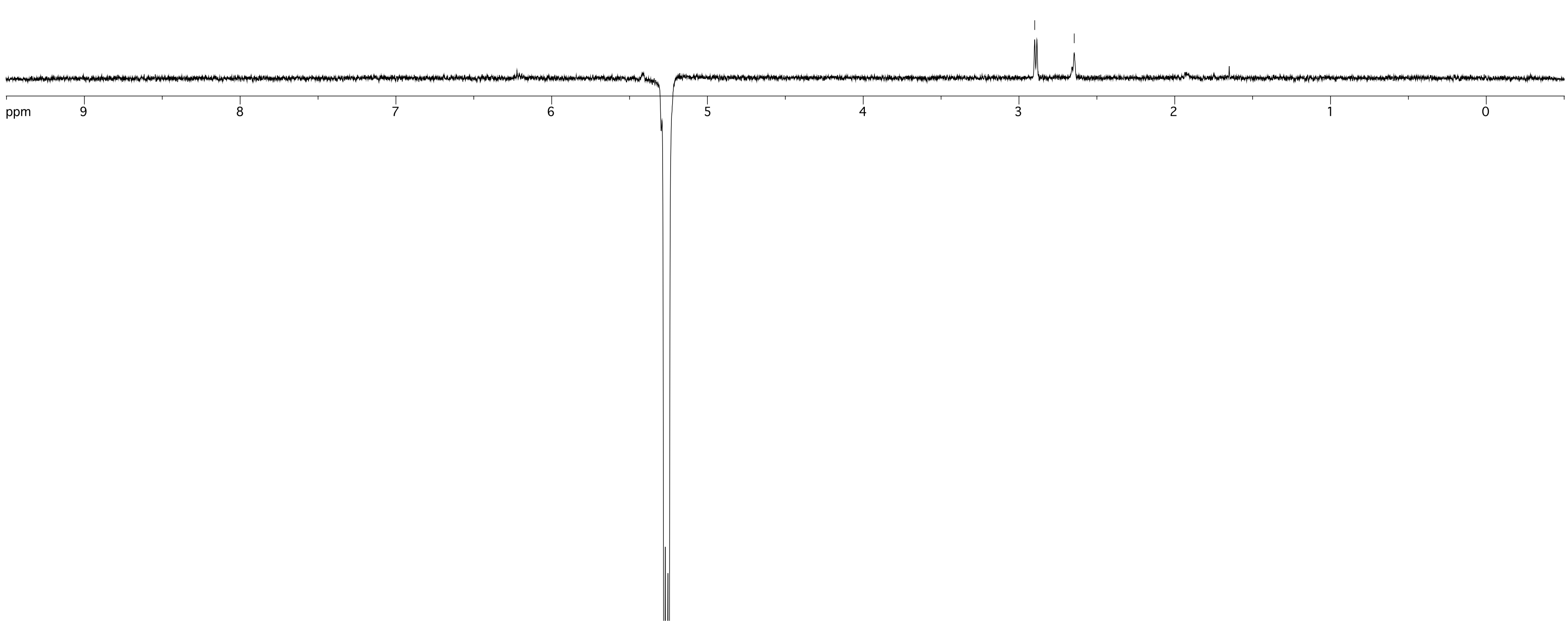

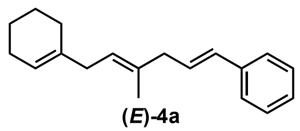

(*E*)-4a

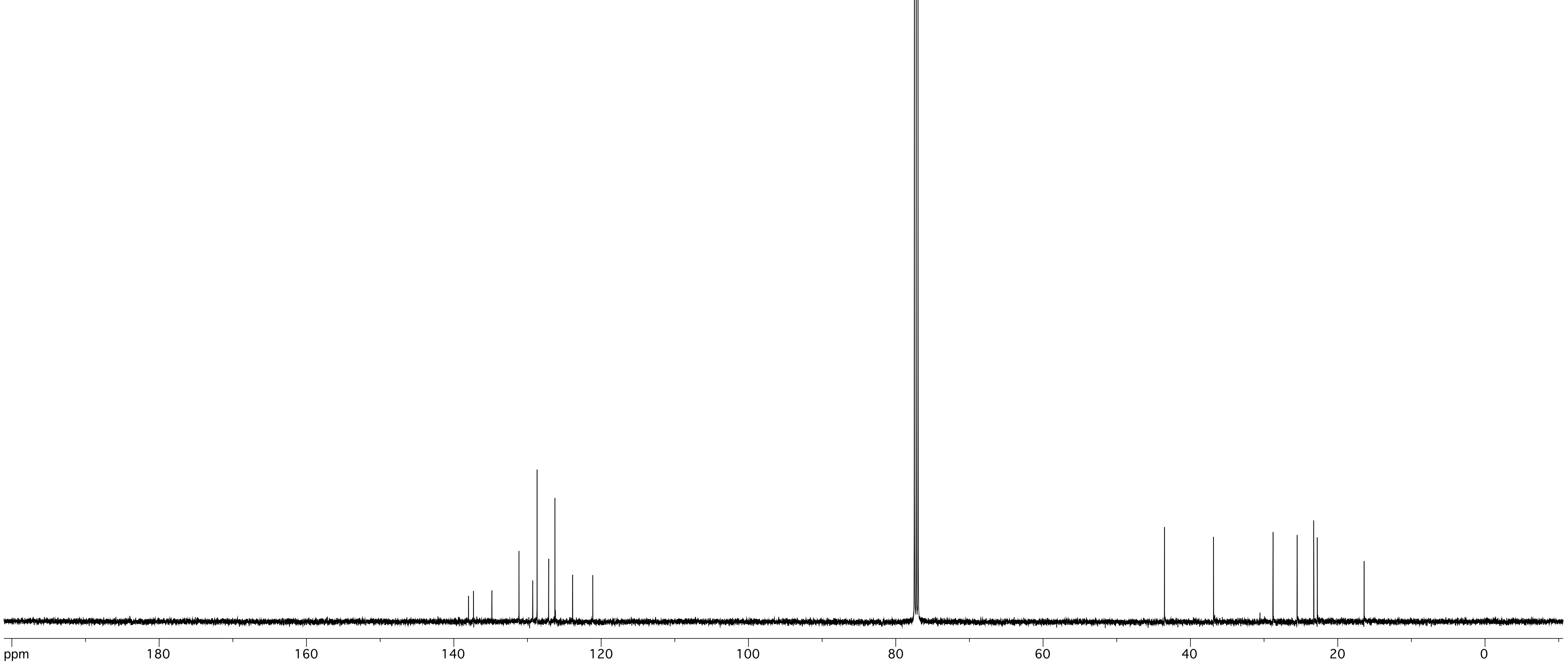

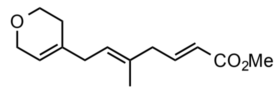
O
CO2Me

(*E*)-4b

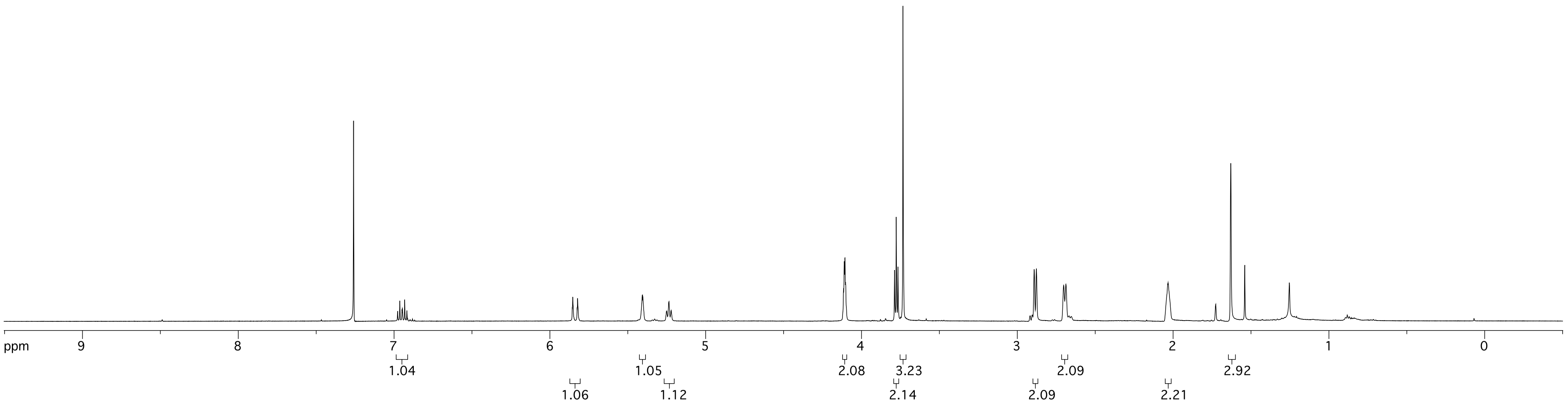
ppm
9
8
7
6
5
4
3
2
1
0
1.04
1.06
1.05
1.12
2.08
3.23
2.14
2.09
2.09
2.21
2.92

NOESY1D

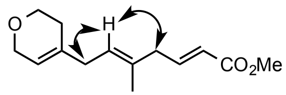

(*E*)-4b

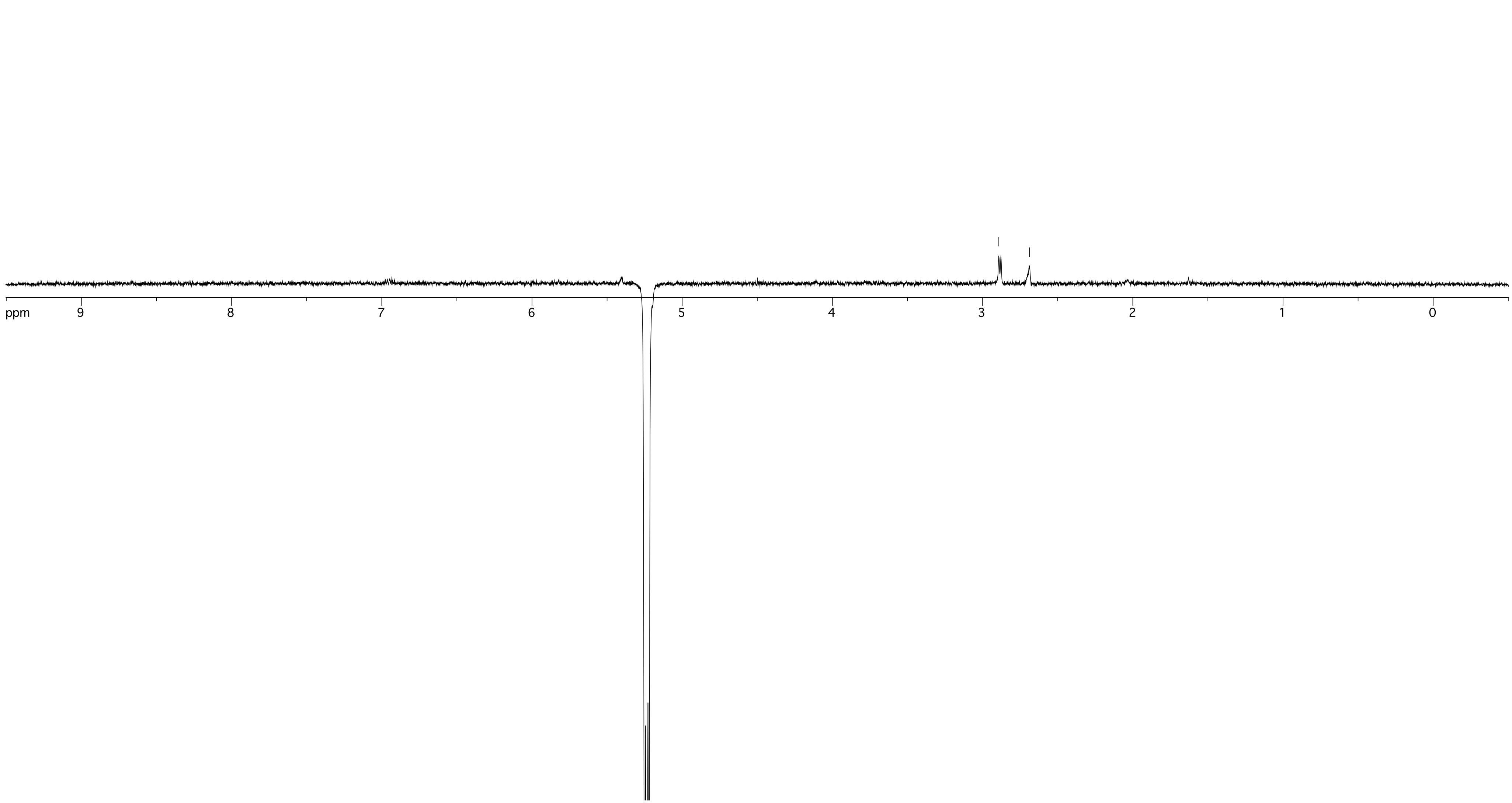

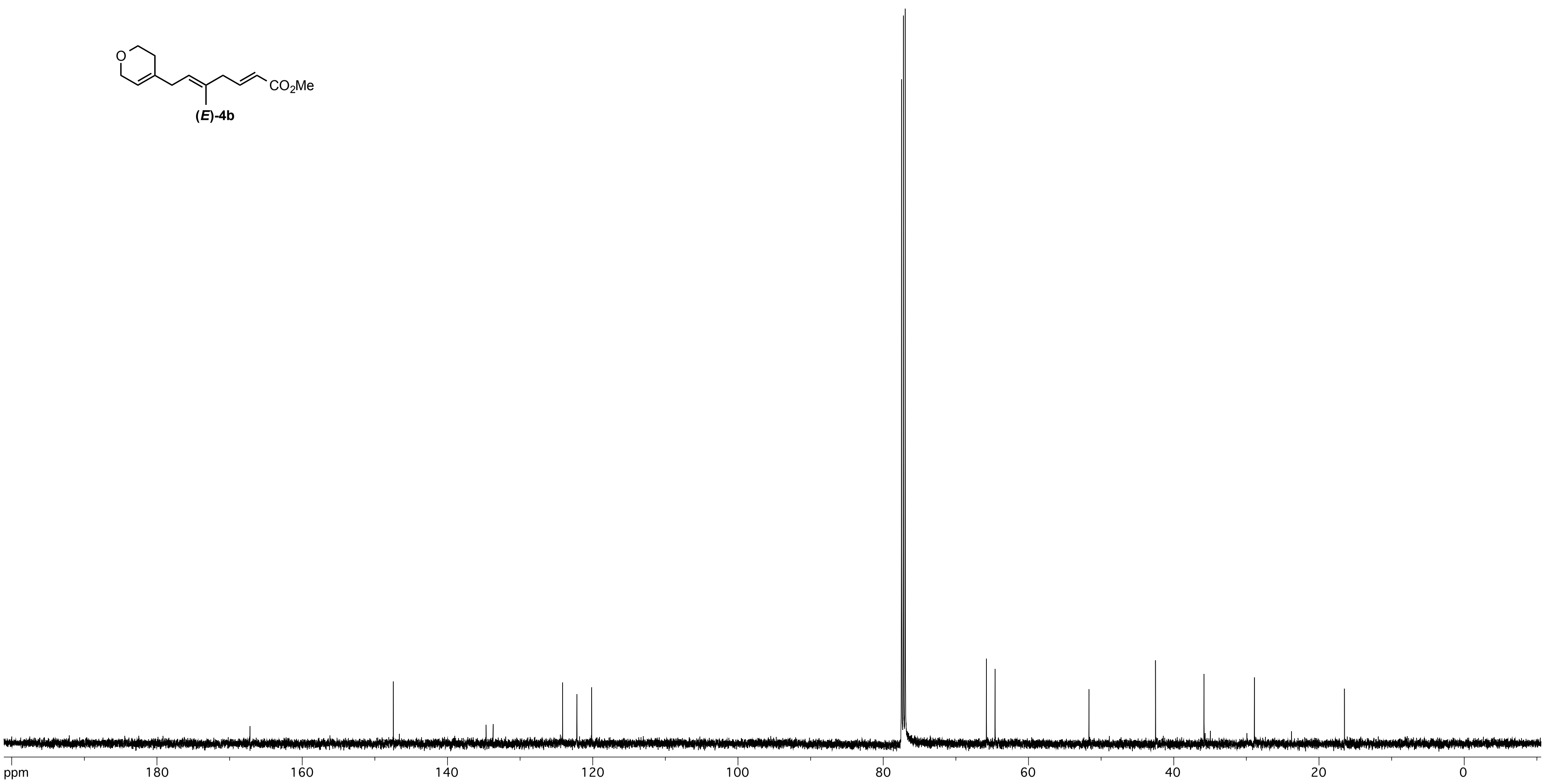

O
CO2Me
(E)-4b
ppm
180
160
140
120
100
80
60
40
20
0

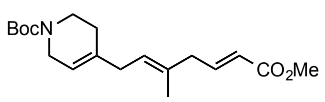
BocN
CO2Me

(*E*)-4c

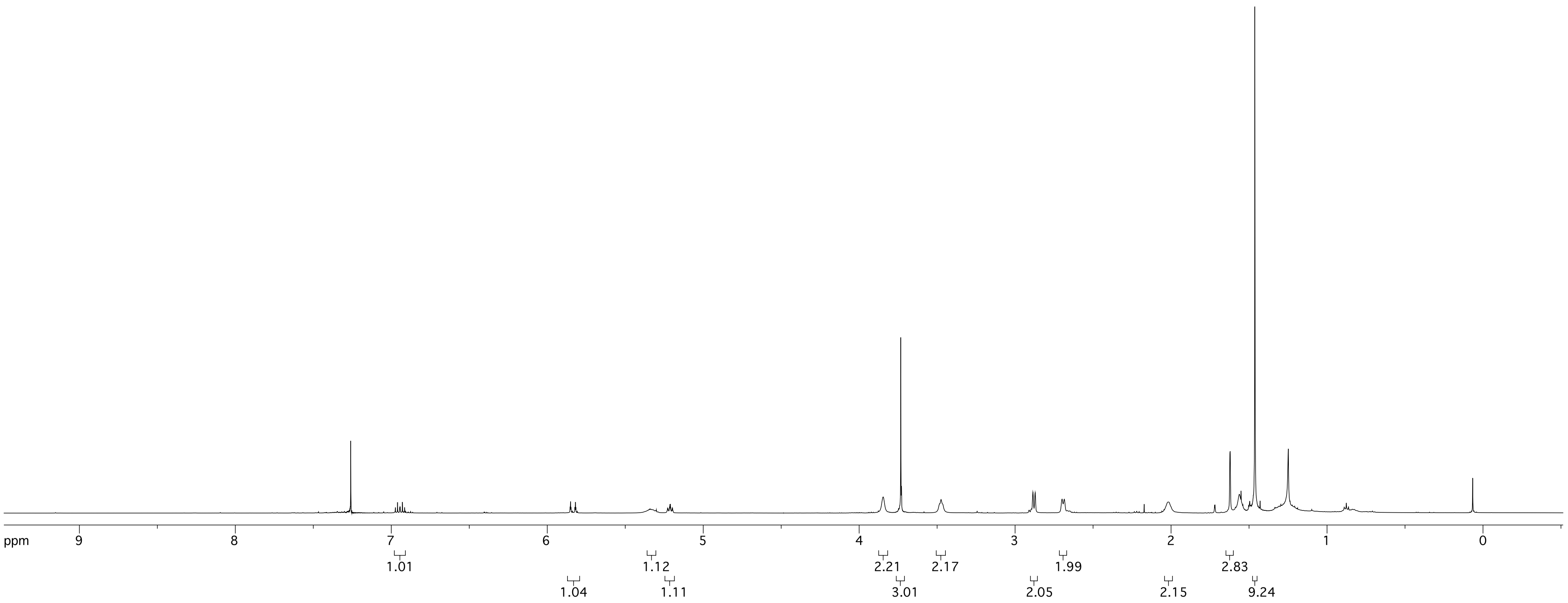
ppm
9
8
7
6
5
4
3
2
1
0
1.01
1.04
1.12
1.11
2.21
3.01
2.17
2.05
1.99
2.15
2.83
9.24

NOESY1D

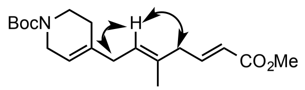

(*E*)-4c

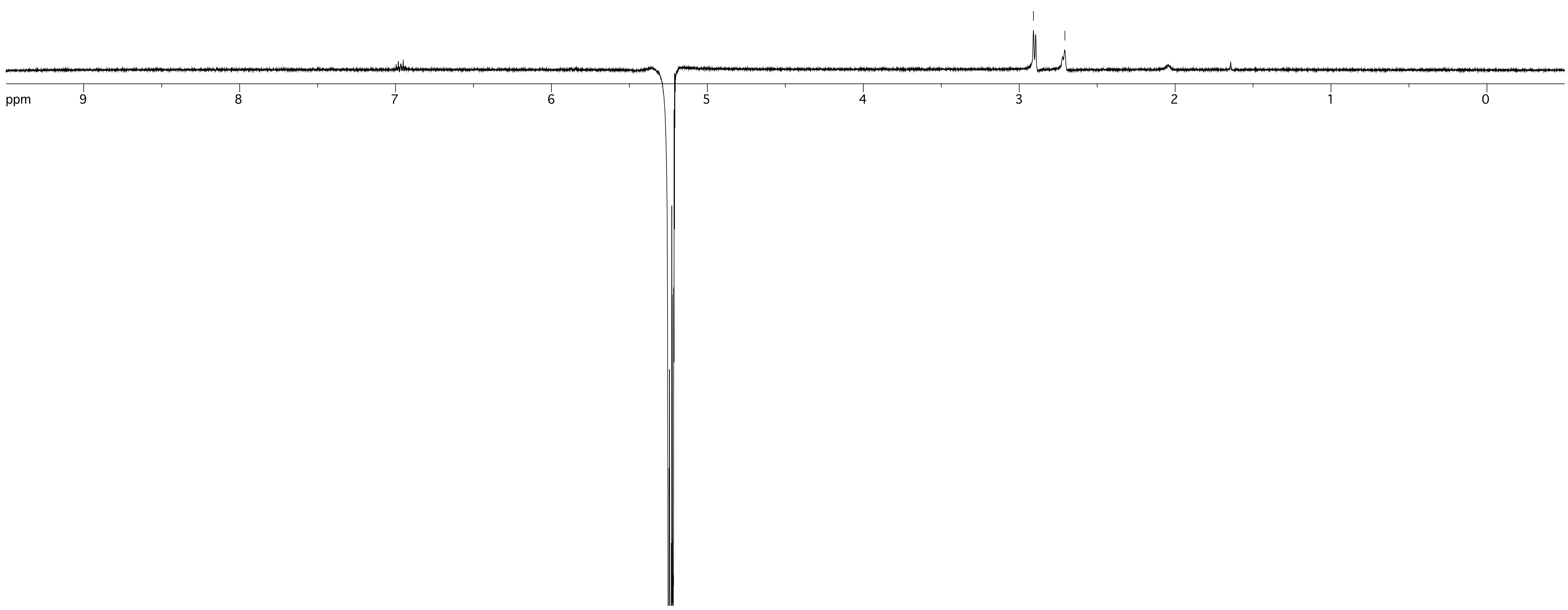

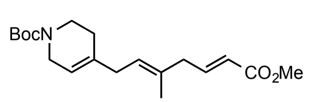

(*E*)-4c

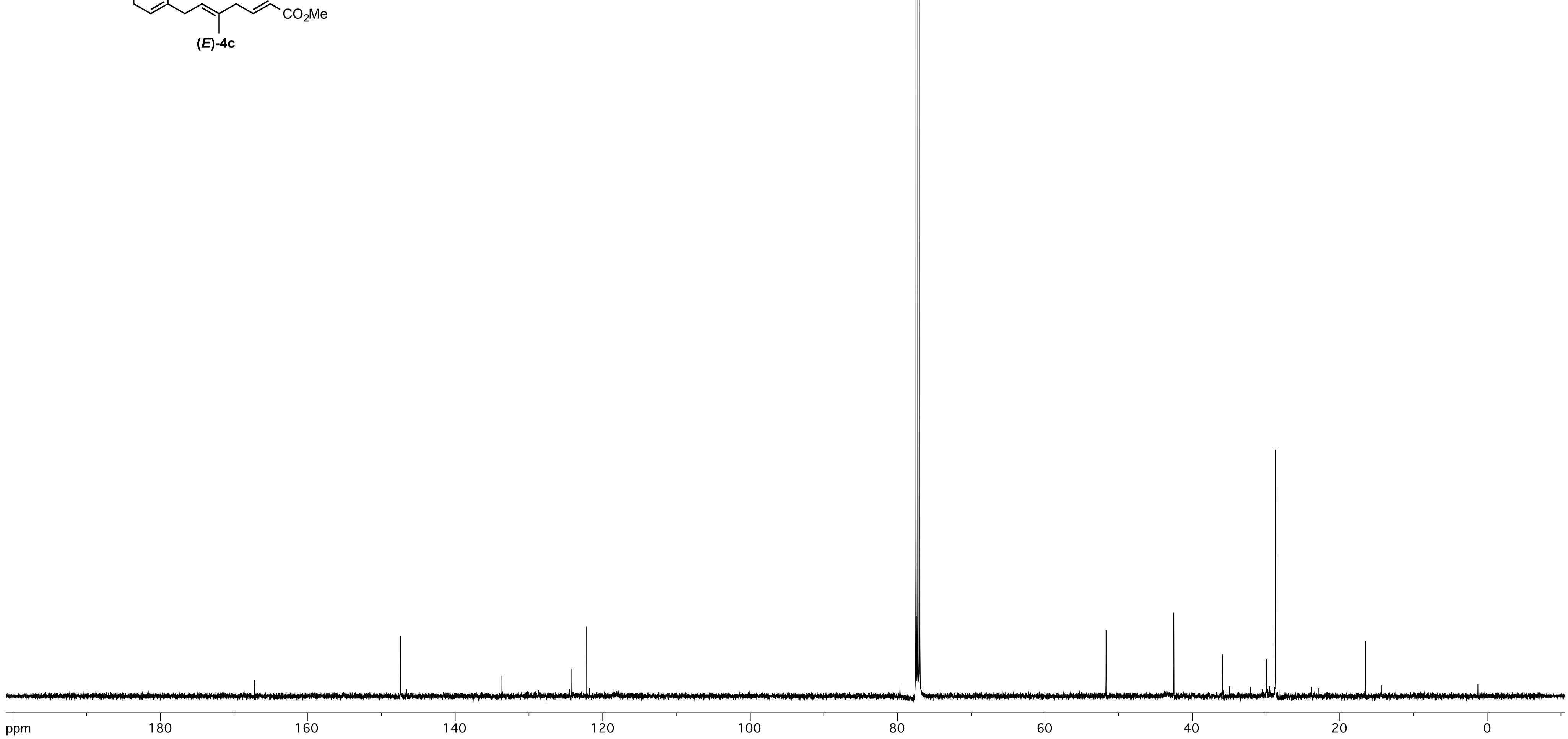

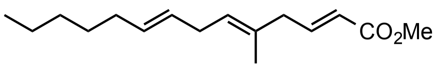
CO2Me

(*E*)-4d

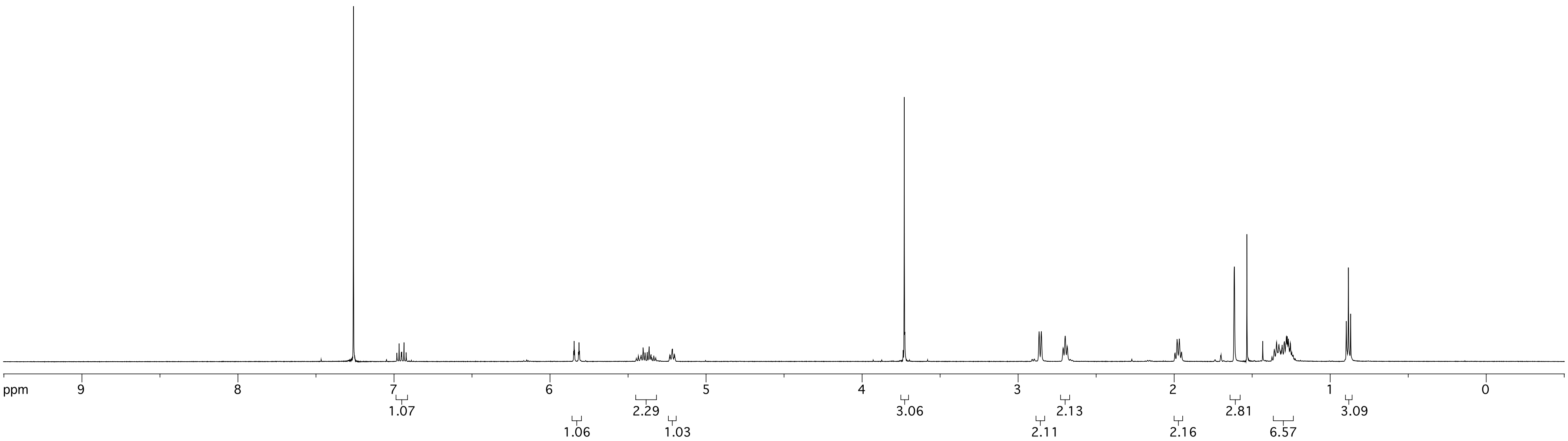
ppm
9
8
7
6
5
4
3
2
1
0
1.07
1.06
2.29
1.03
3.06
2.11
2.13
2.16
2.81
6.57
3.09

NOESY1D

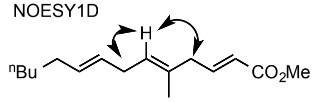

(*E*)-4d

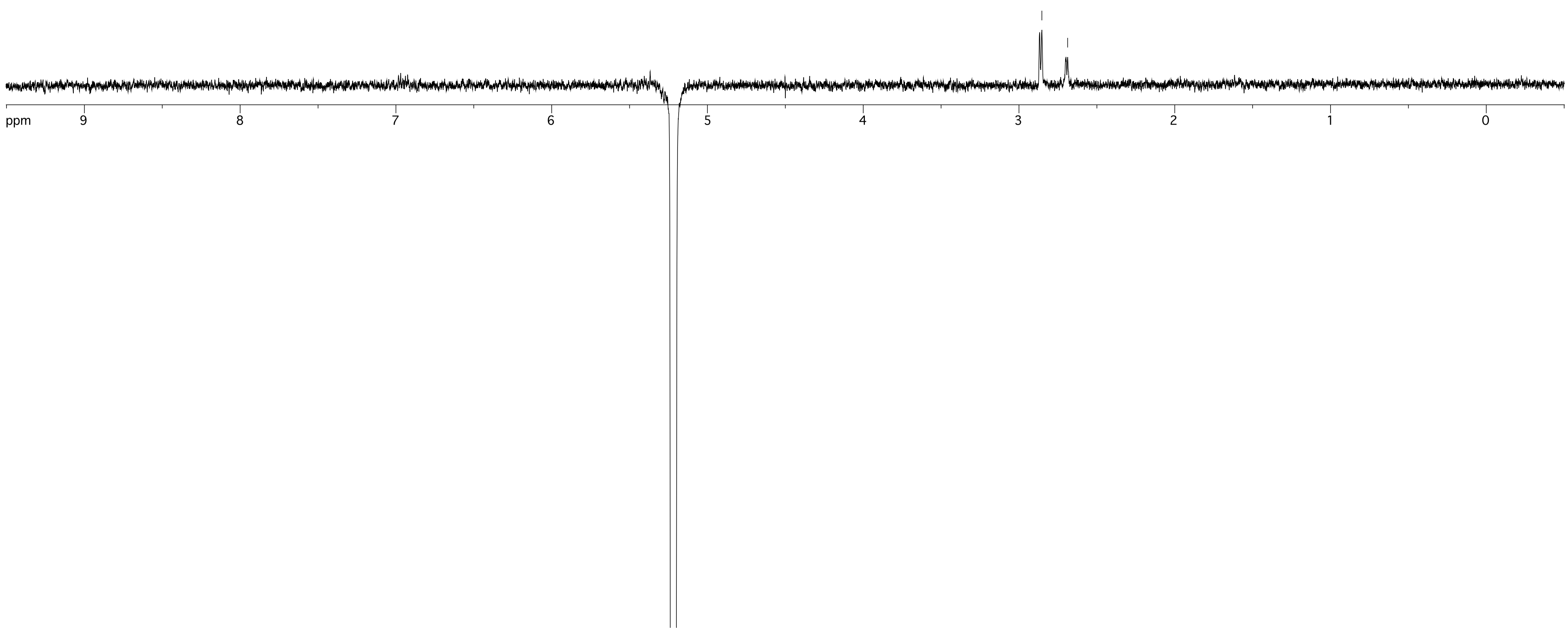

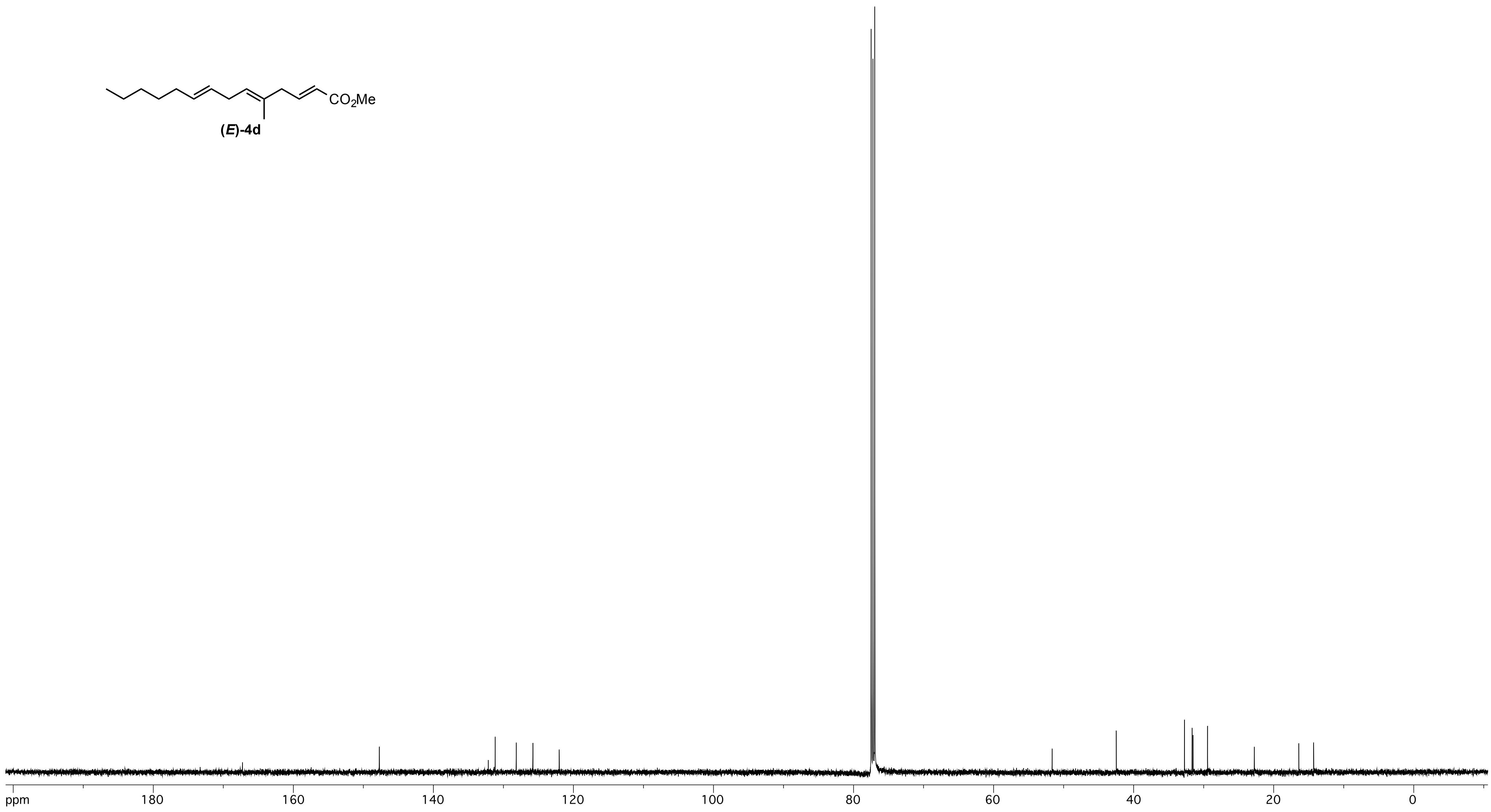
CO2Me
(E)-4d
ppm
180
160
140
120
100
80
60
40
20
0

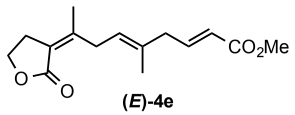

(*E*)-4e

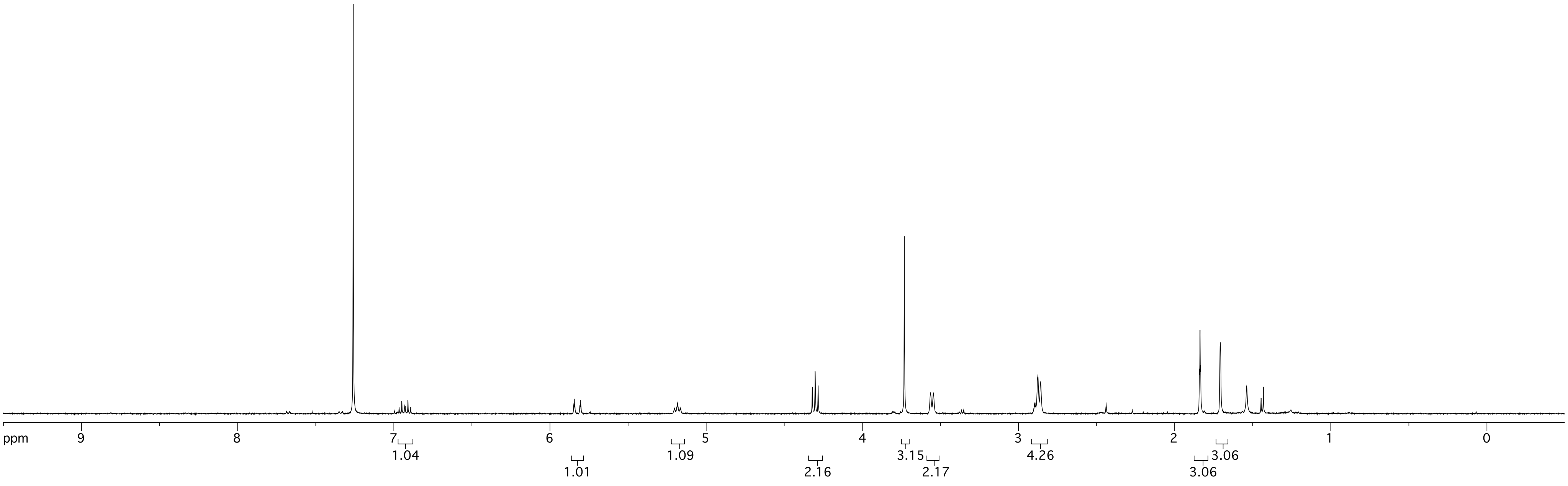

NOESY1D

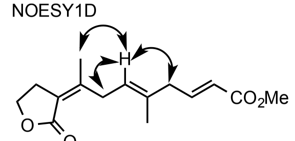

(*E*)-4e

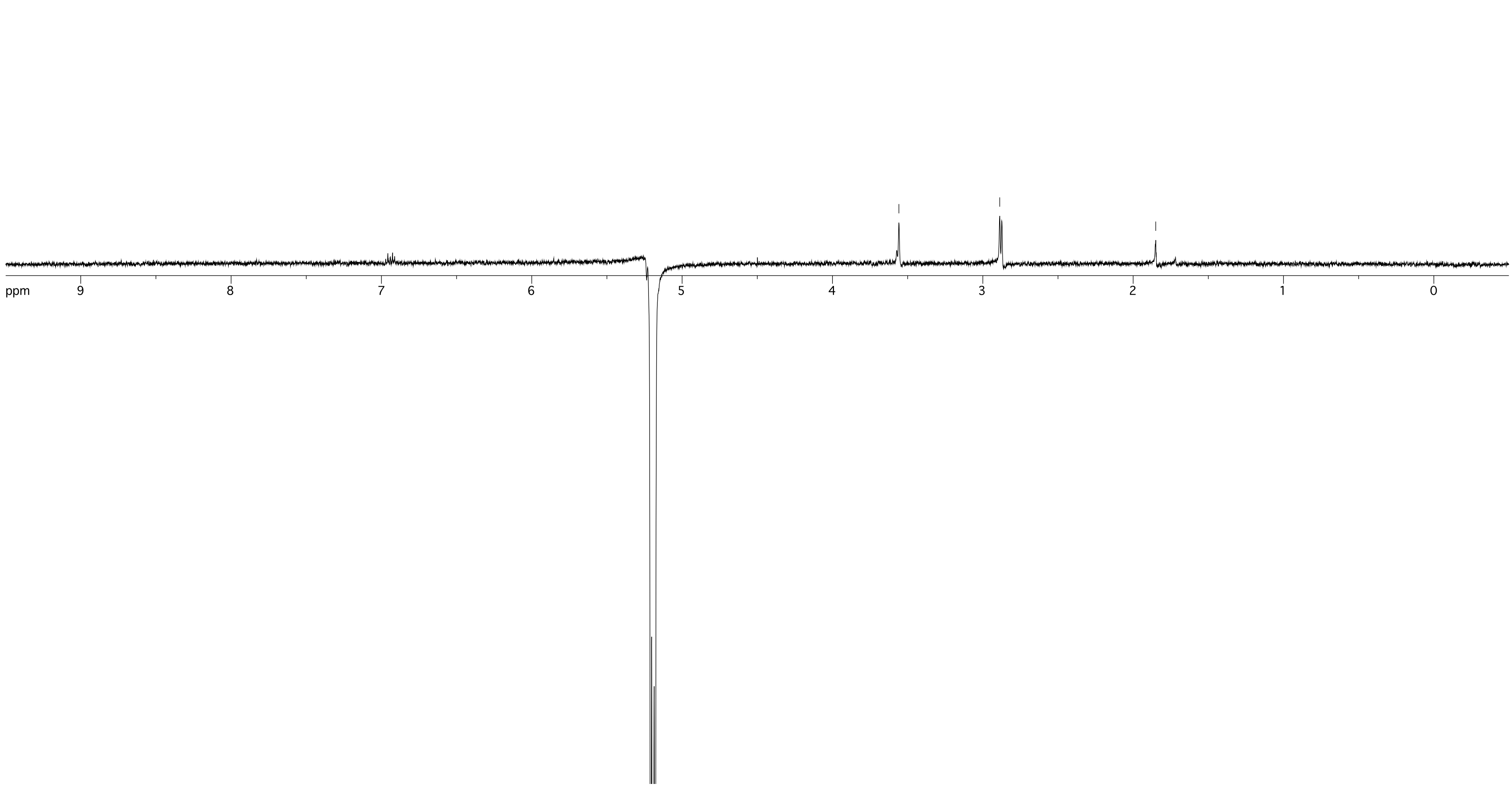

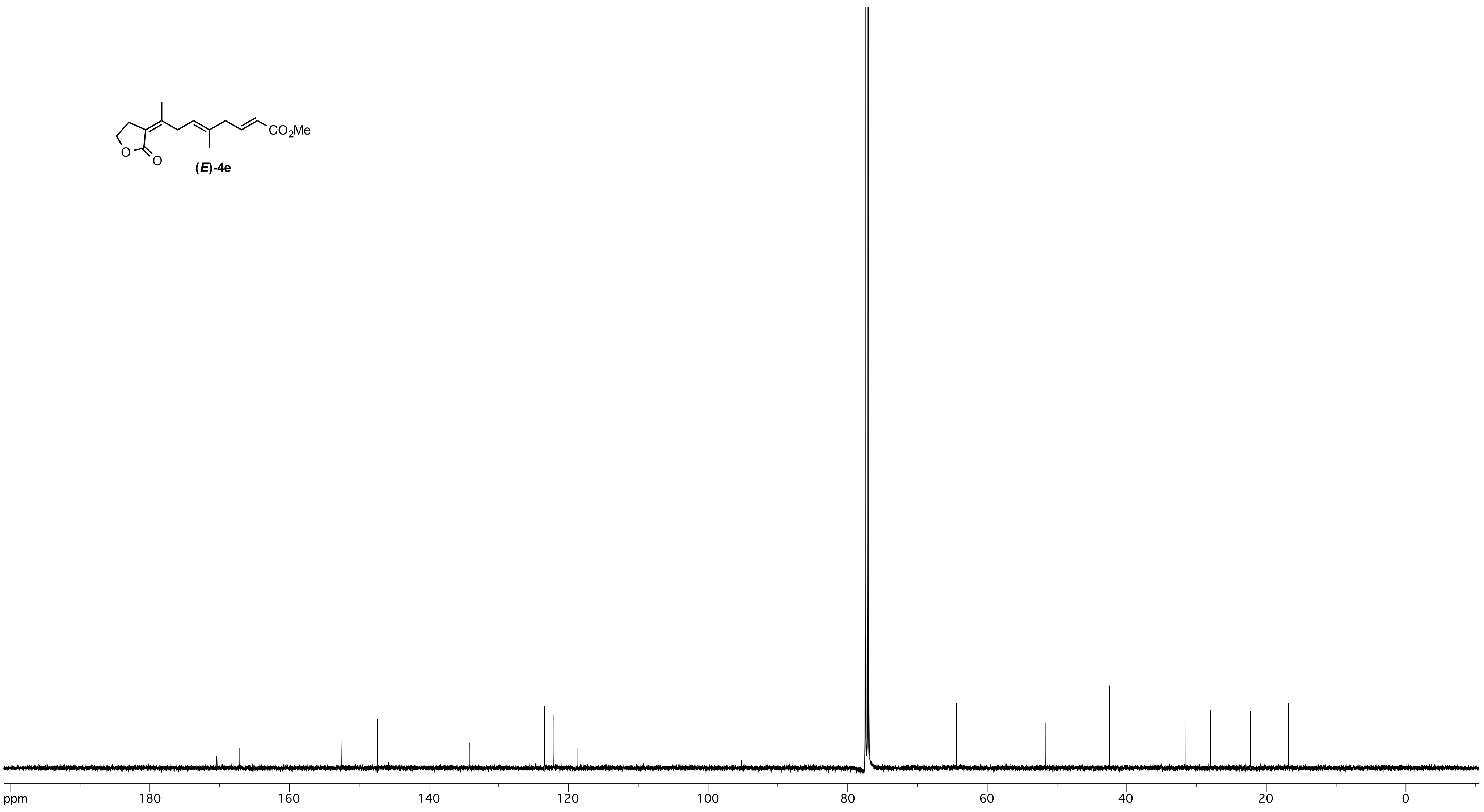

CO2Me
O
O
(E)-4e
ppm
180
160
140
120
100
80
60
40
20
0

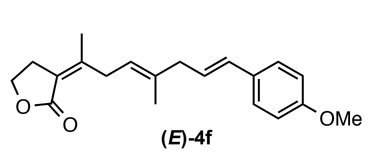
O
O
OMe

(*E*)-4f

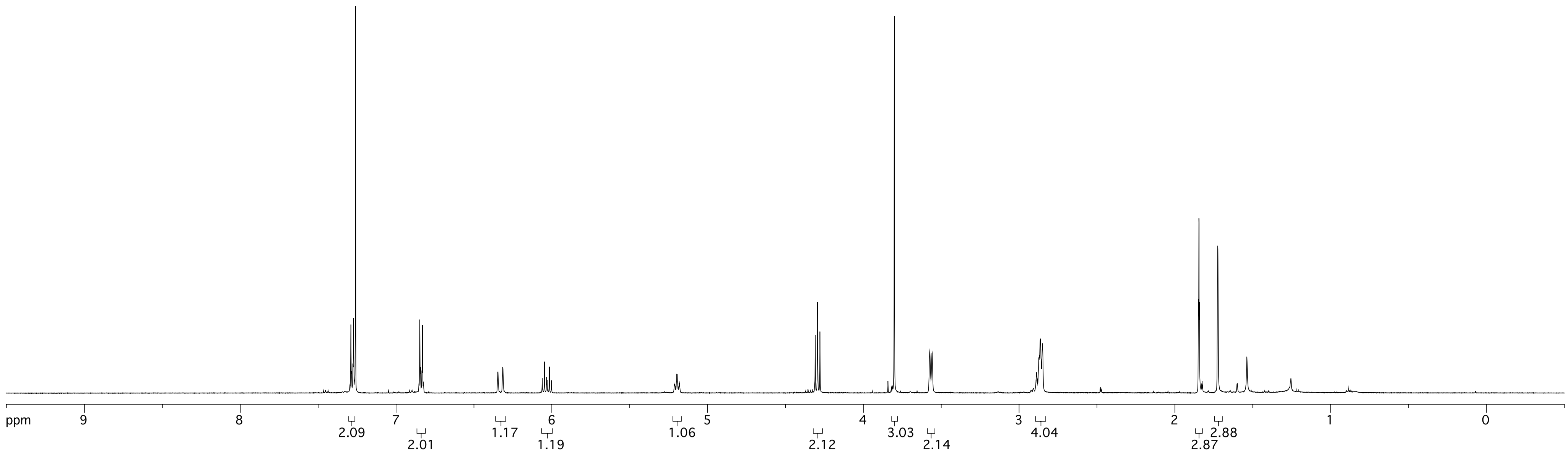
ppm
9
8
7
6
5
4
3
2
1
0
2.09
2.01
1.17
1.19
1.06
2.12
3.03
2.14
4.04
2.87
2.88

NOESY1D

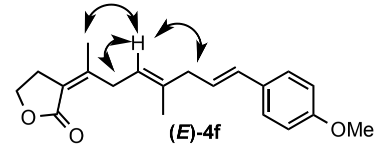

(*E*)-4f

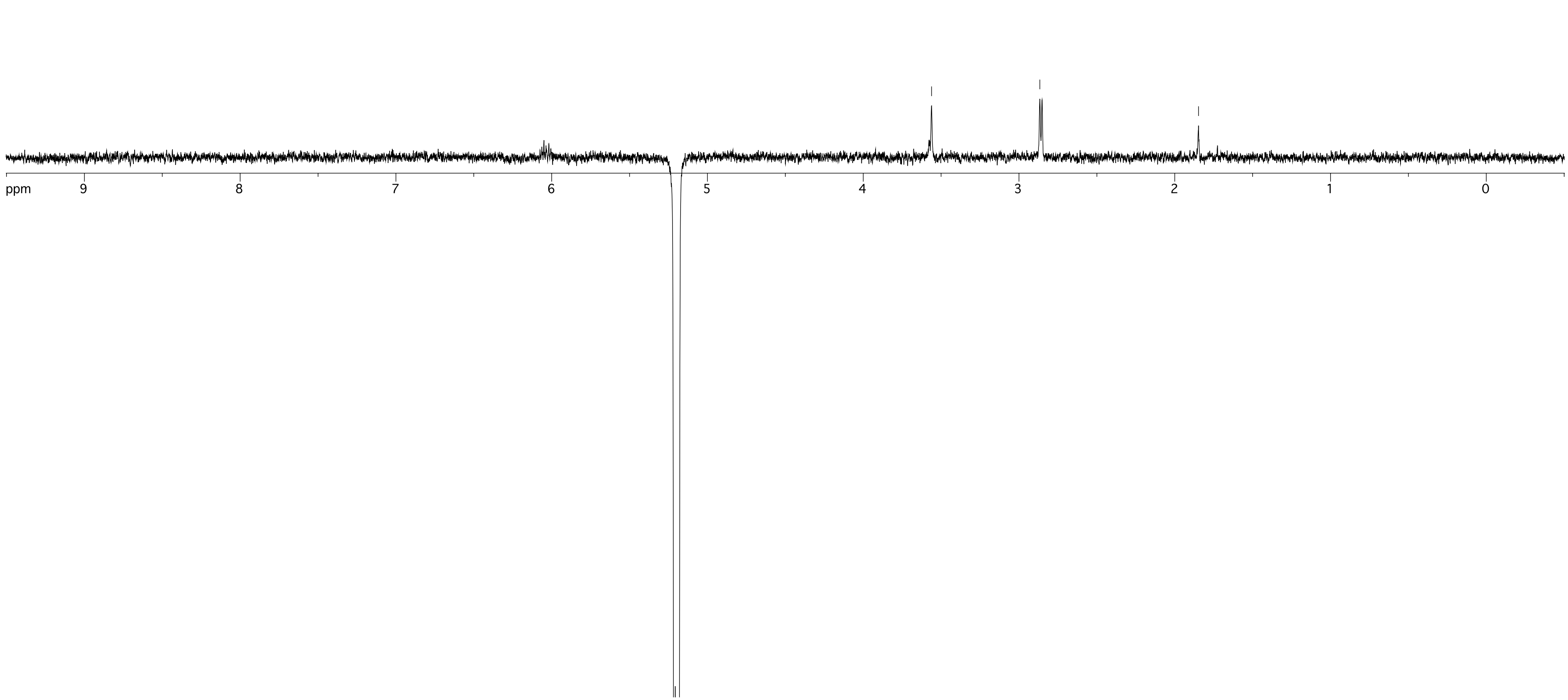

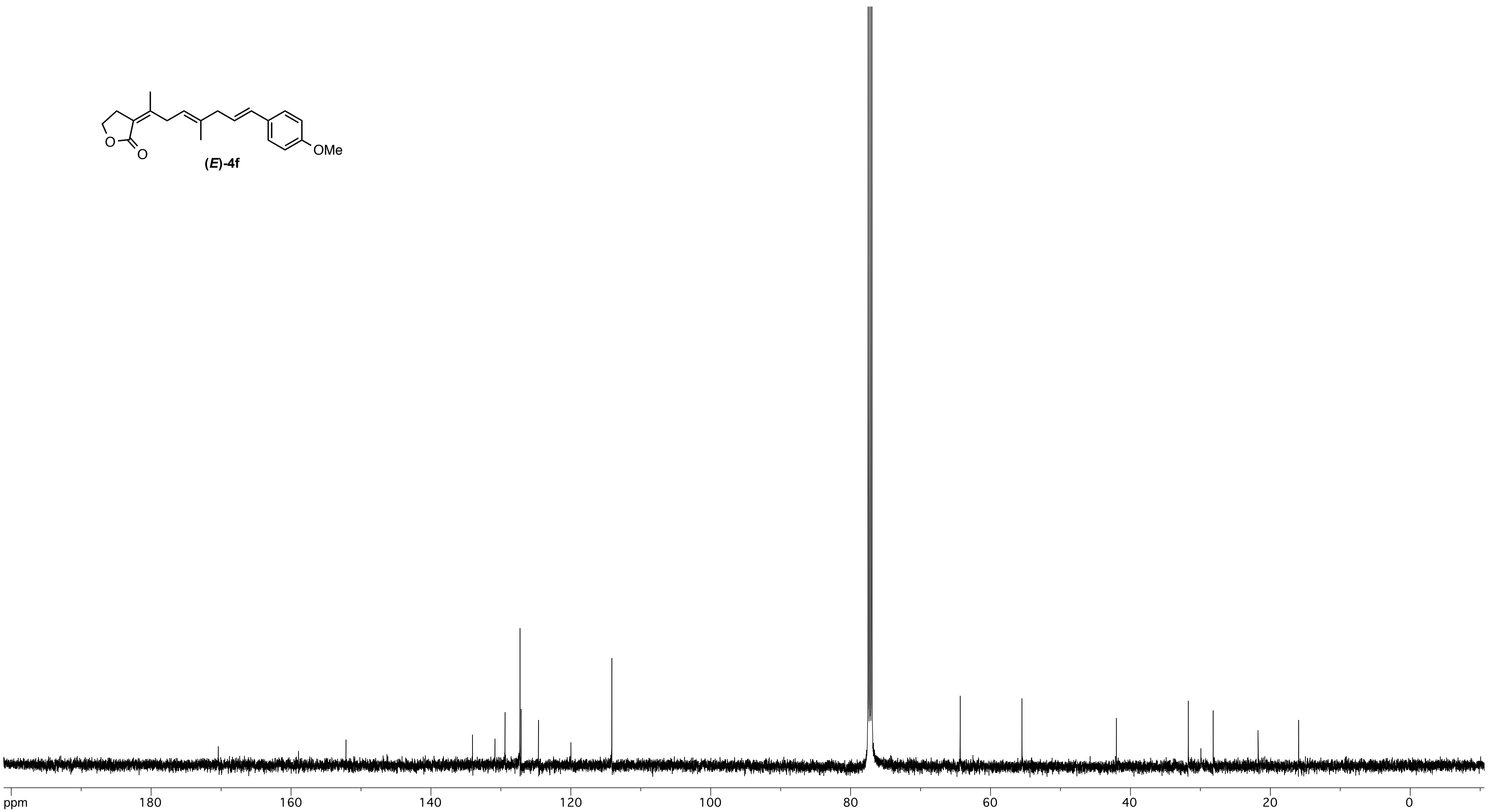

(*E*)-4f

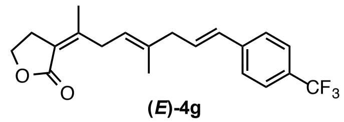

(*E*)-4g

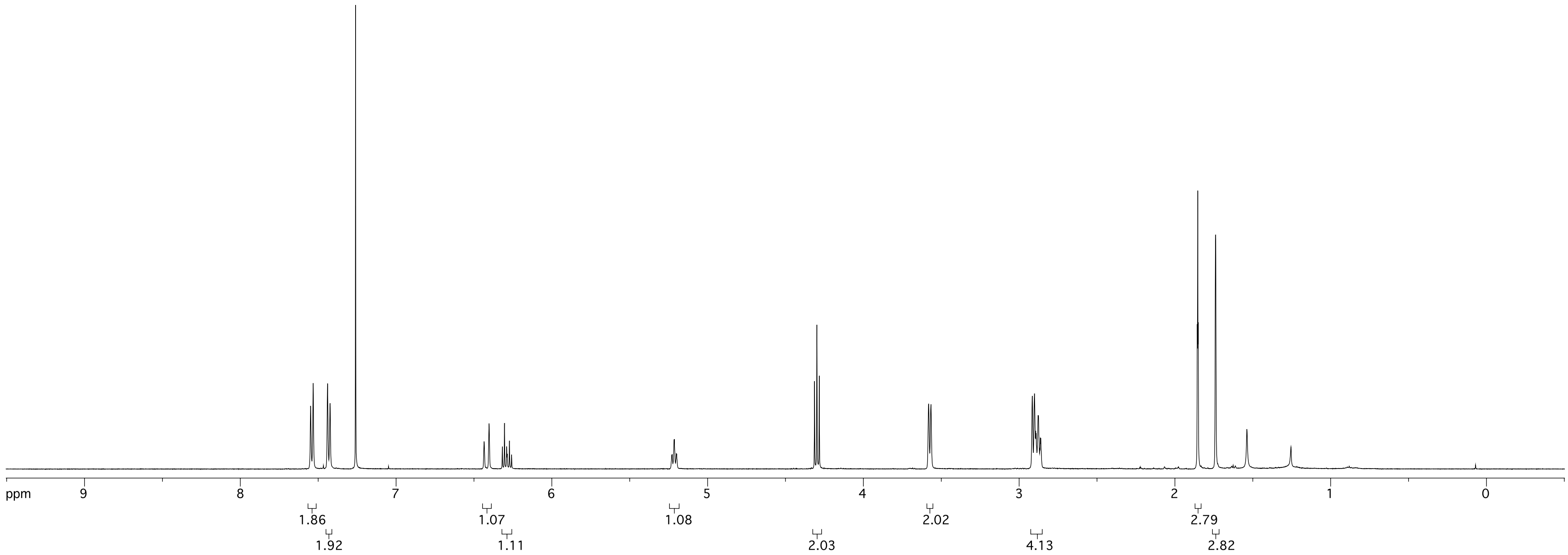

NOESY1D

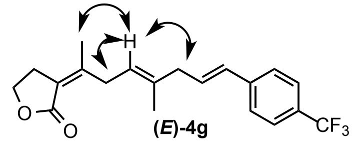

(*E*)-4g

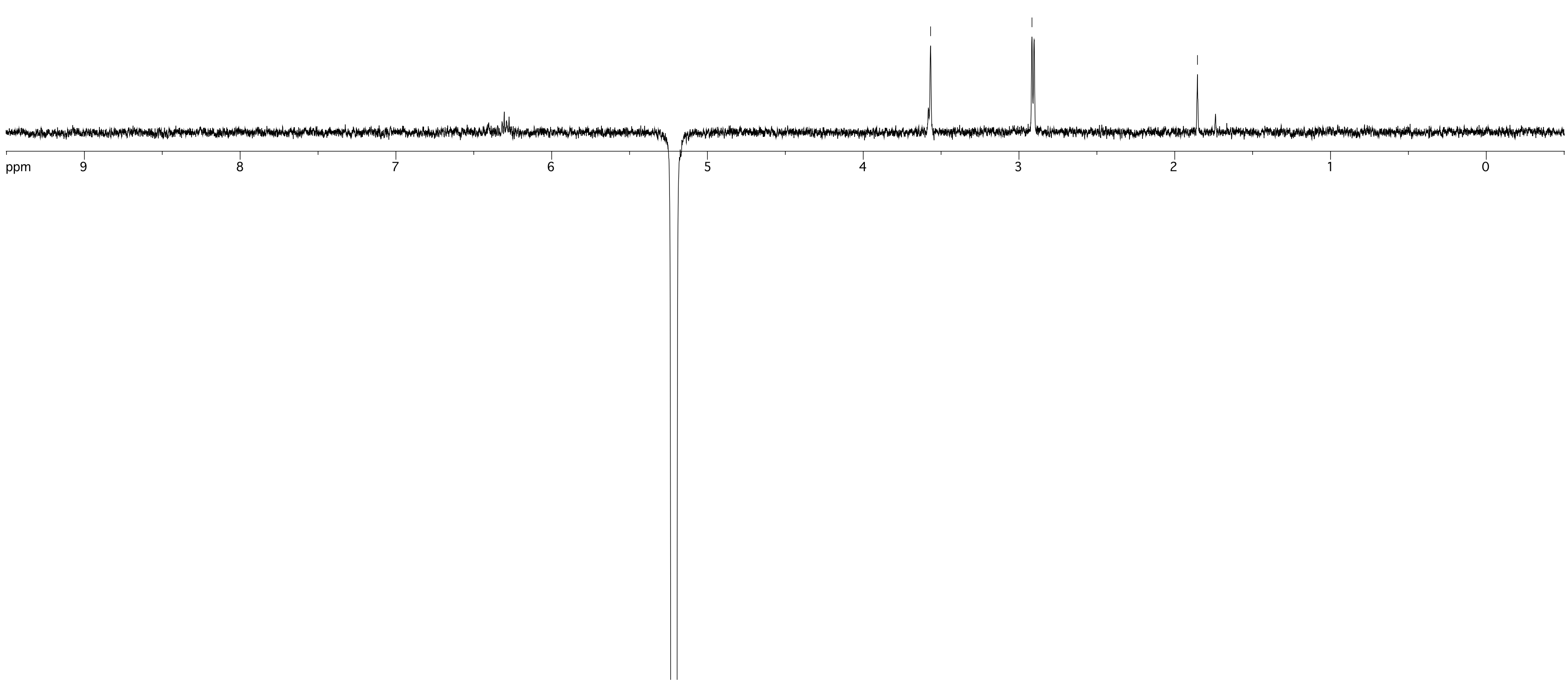

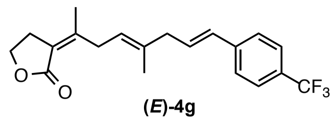

(*E*)-4g

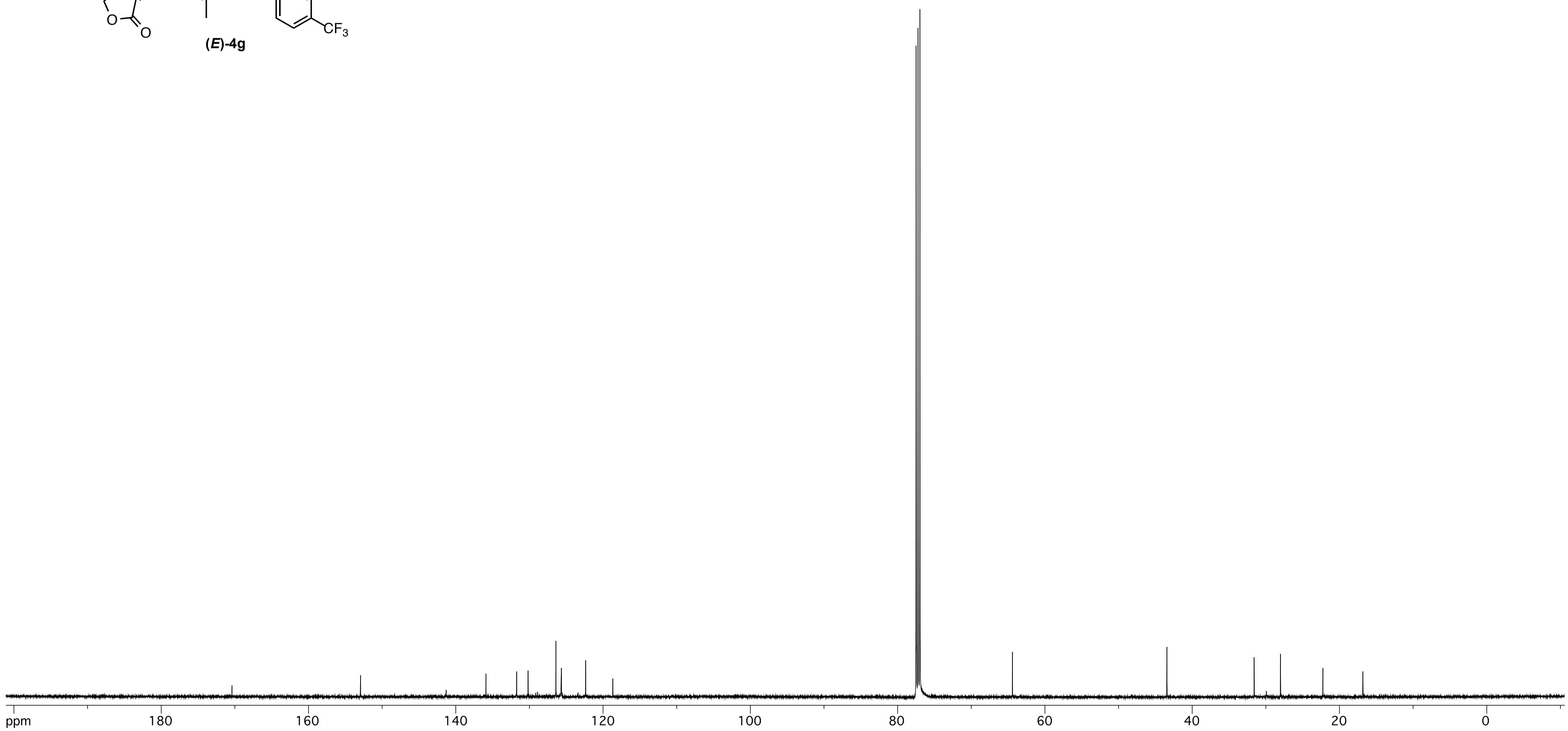

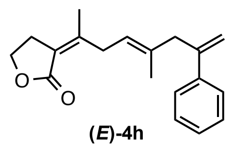

(*E*)-4h

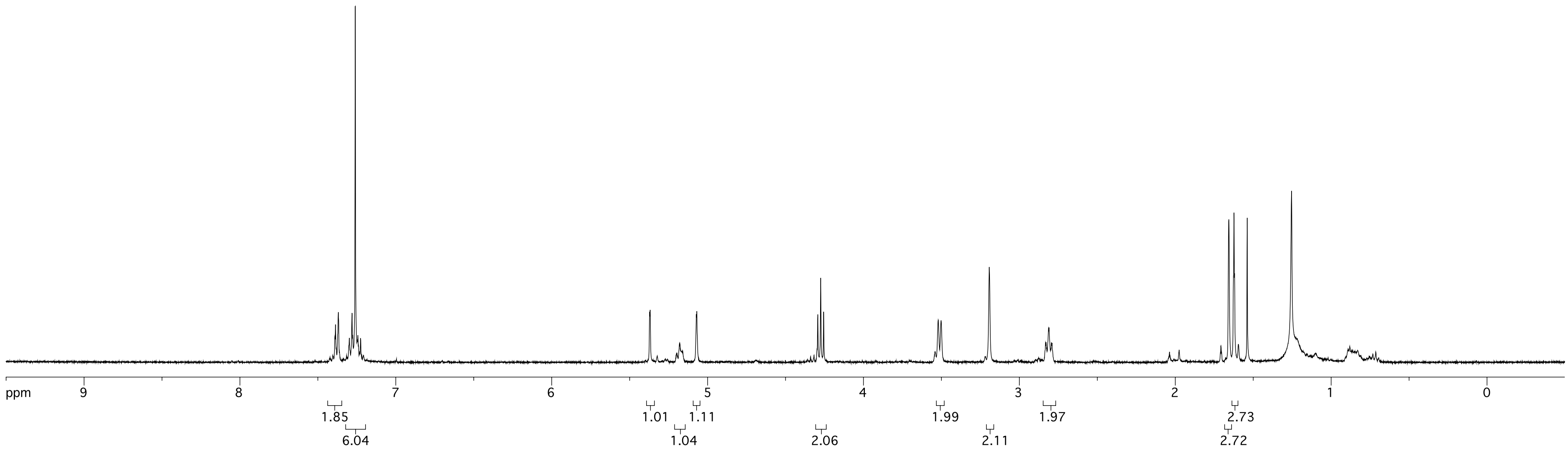

NOESY1D

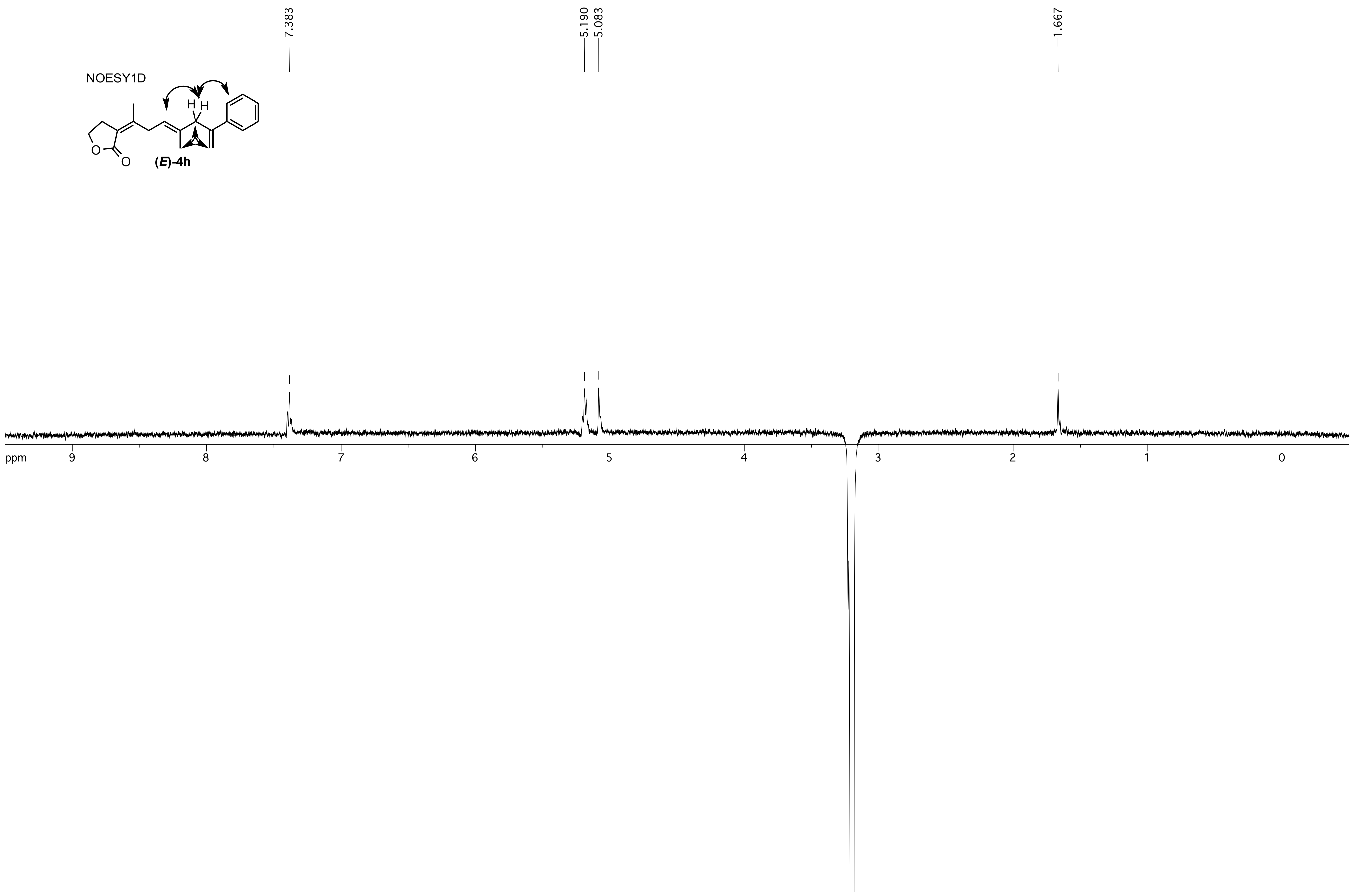

(*E*)-4h

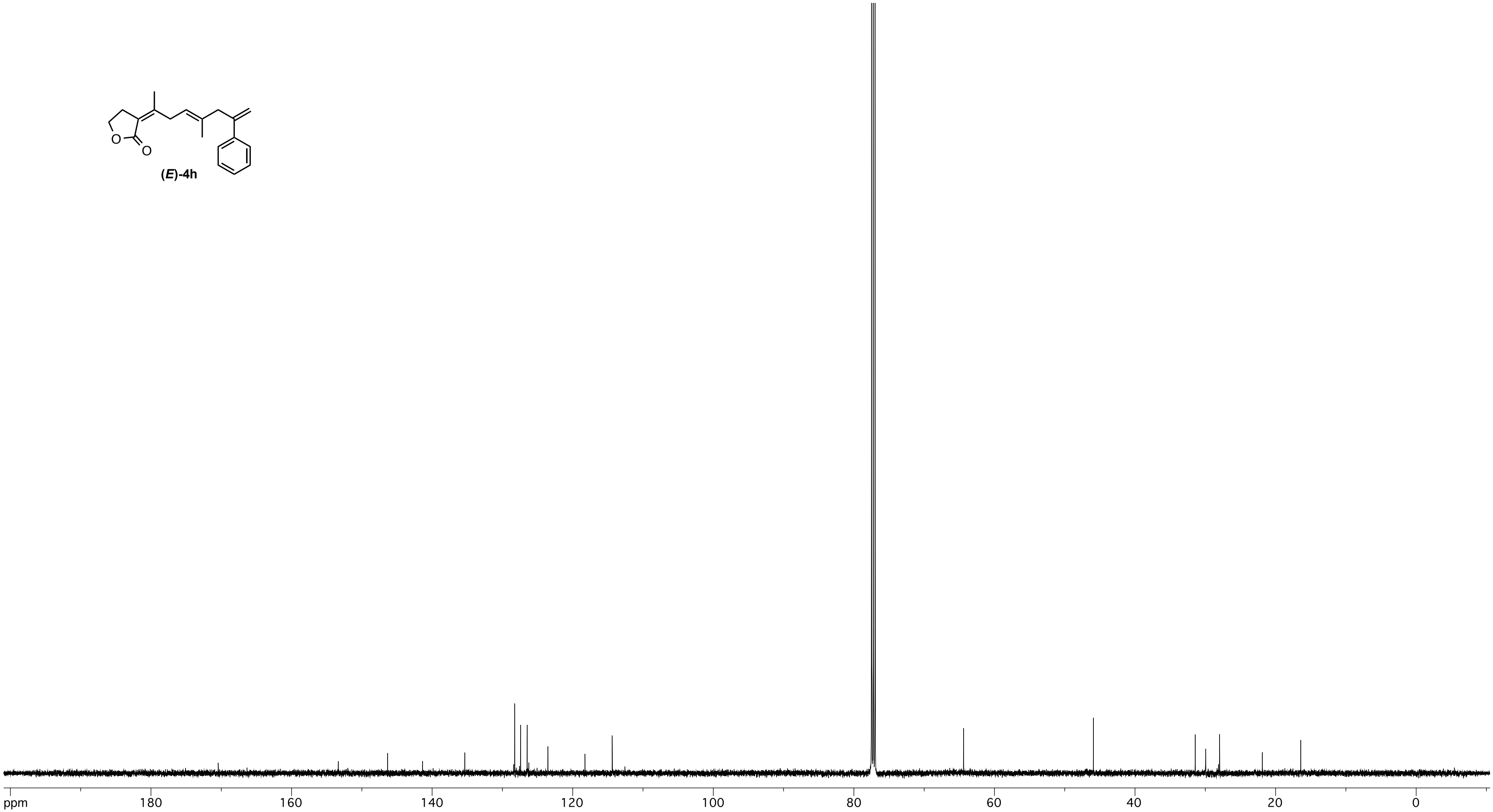
(*E*)-4h
ppm
180
160
140
120
100
80
60
40
20
0

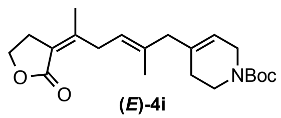
O
O
NBoc

(*E*)-4i

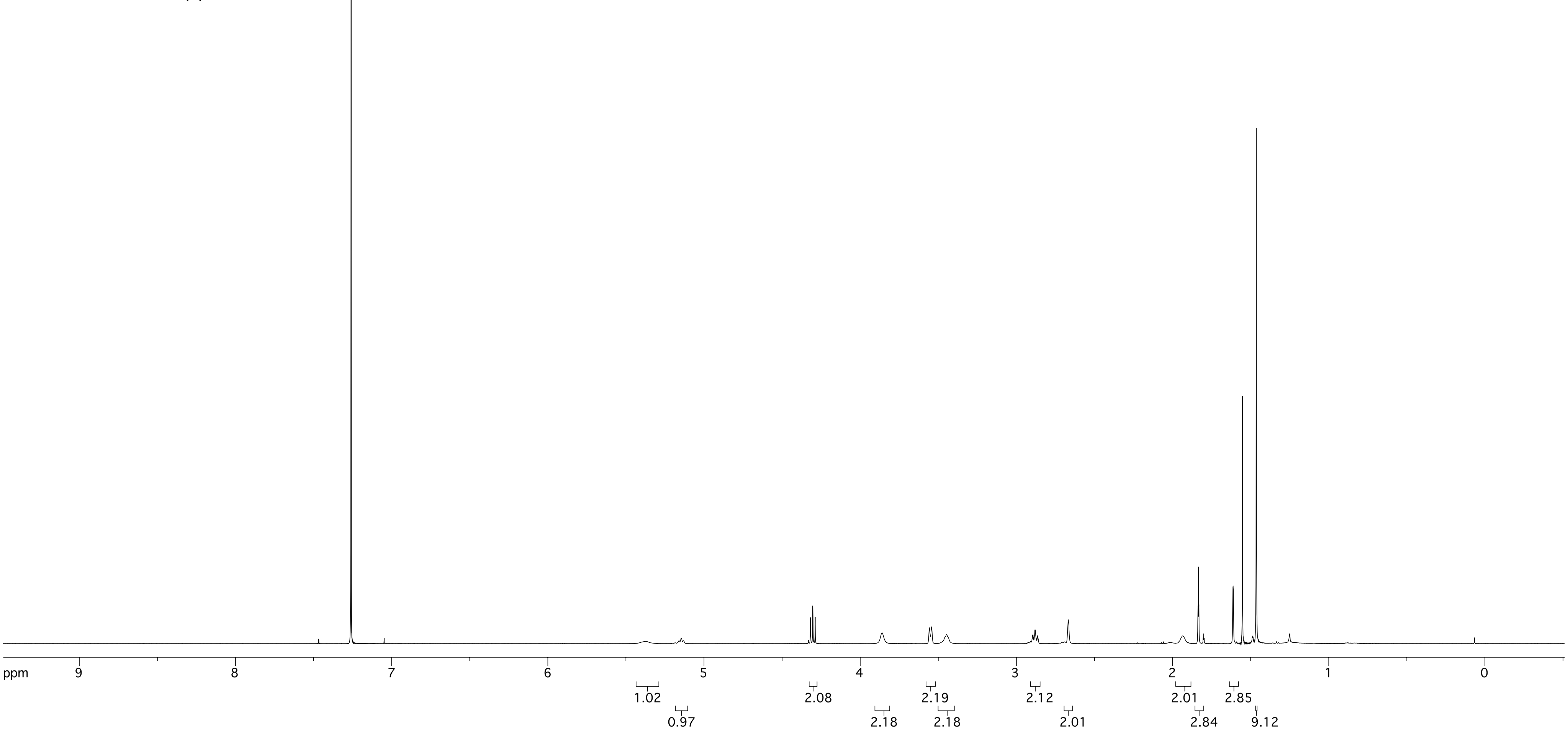
ppm
9
8
7
6
5
4
3
2
1
0
1.02
0.97
2.08
2.18
2.19
2.18
2.12
2.01
2.01
2.84
2.85
9.12

NOESY1D

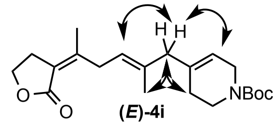

(*E*)-4i

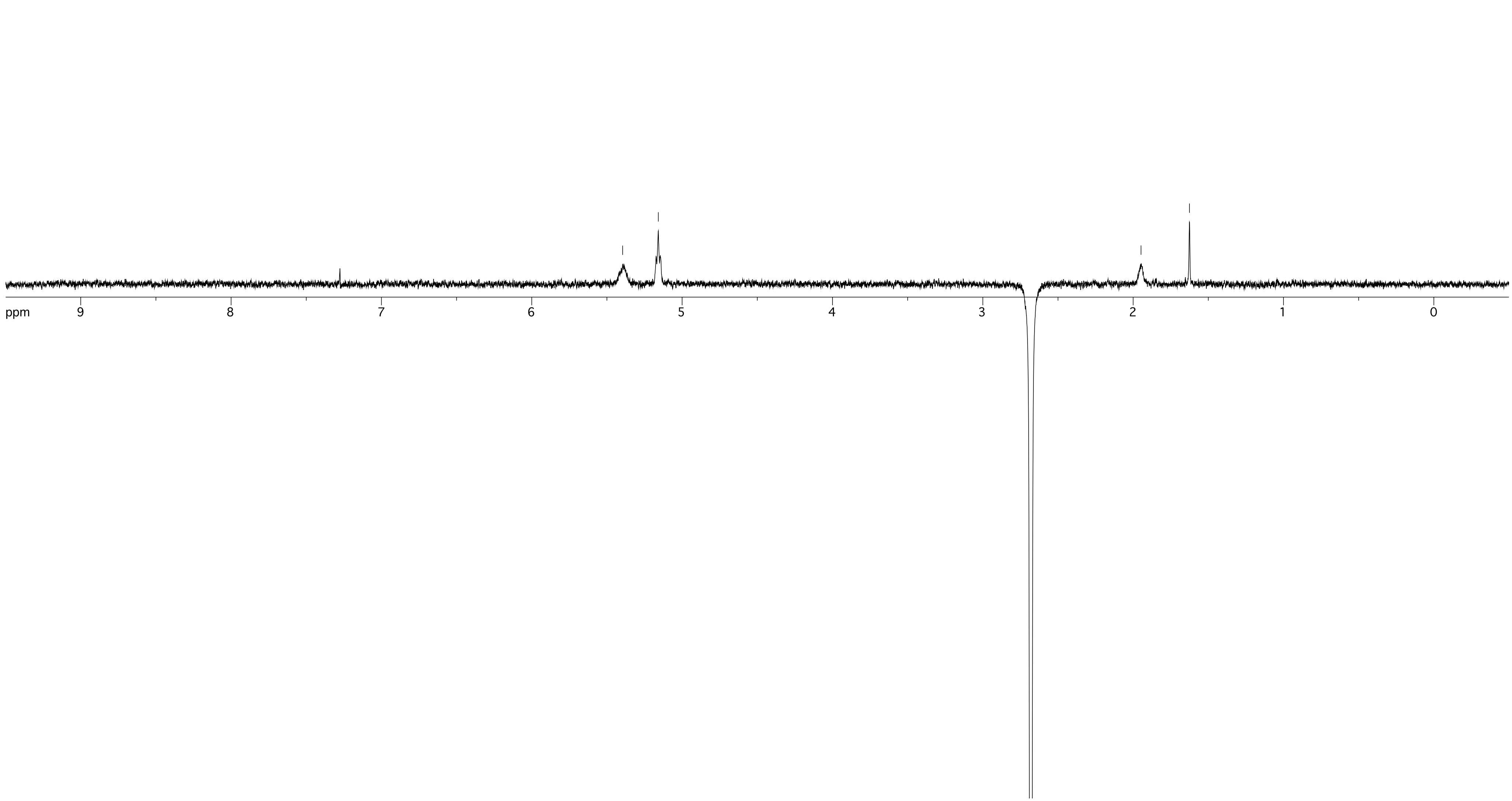

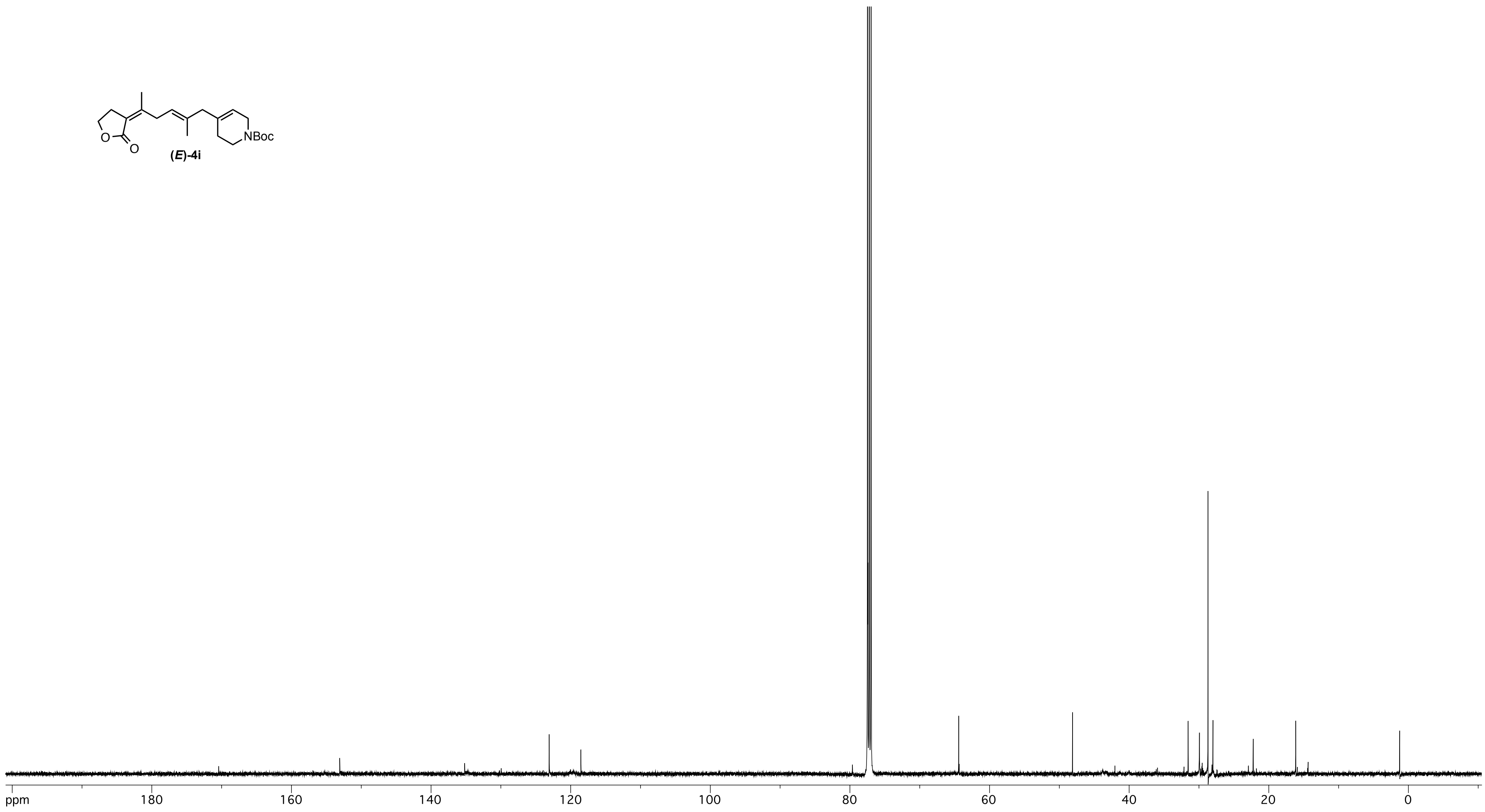
NBoc
O
O
(E)-4i
ppm
180
160
140
120
100
80
60
40
20
0

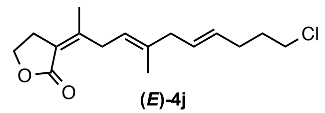

(*E*)-4j

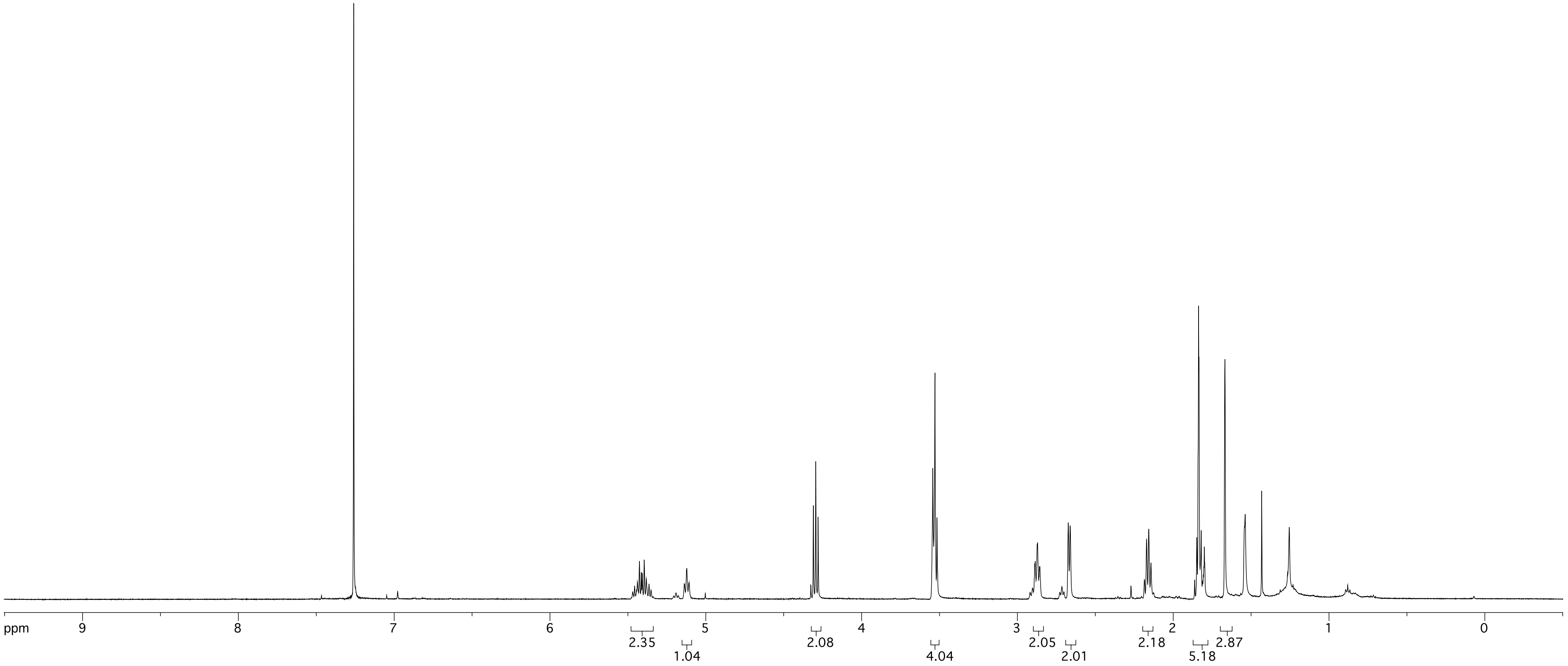

NOESY1D

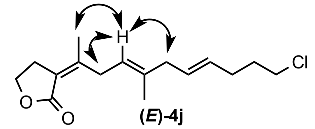

(*E*)-4j

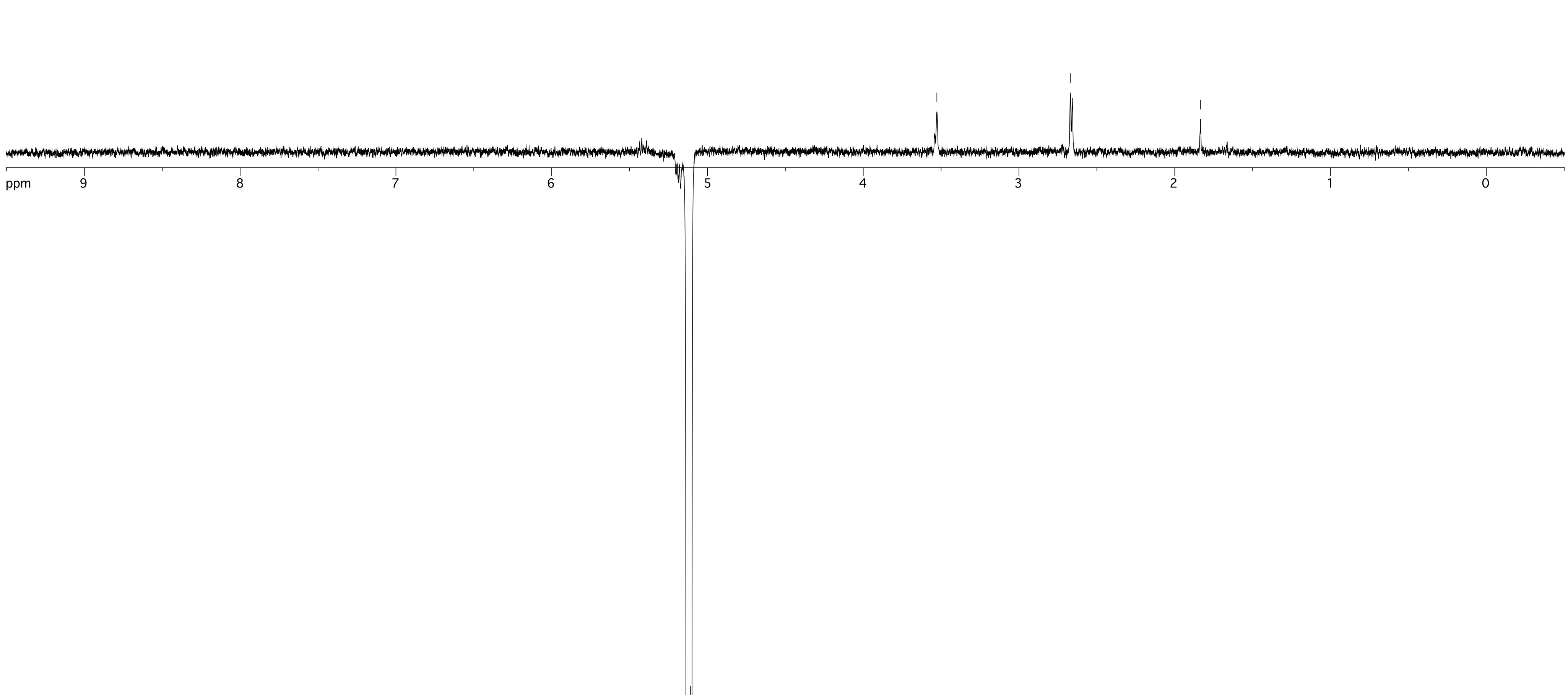

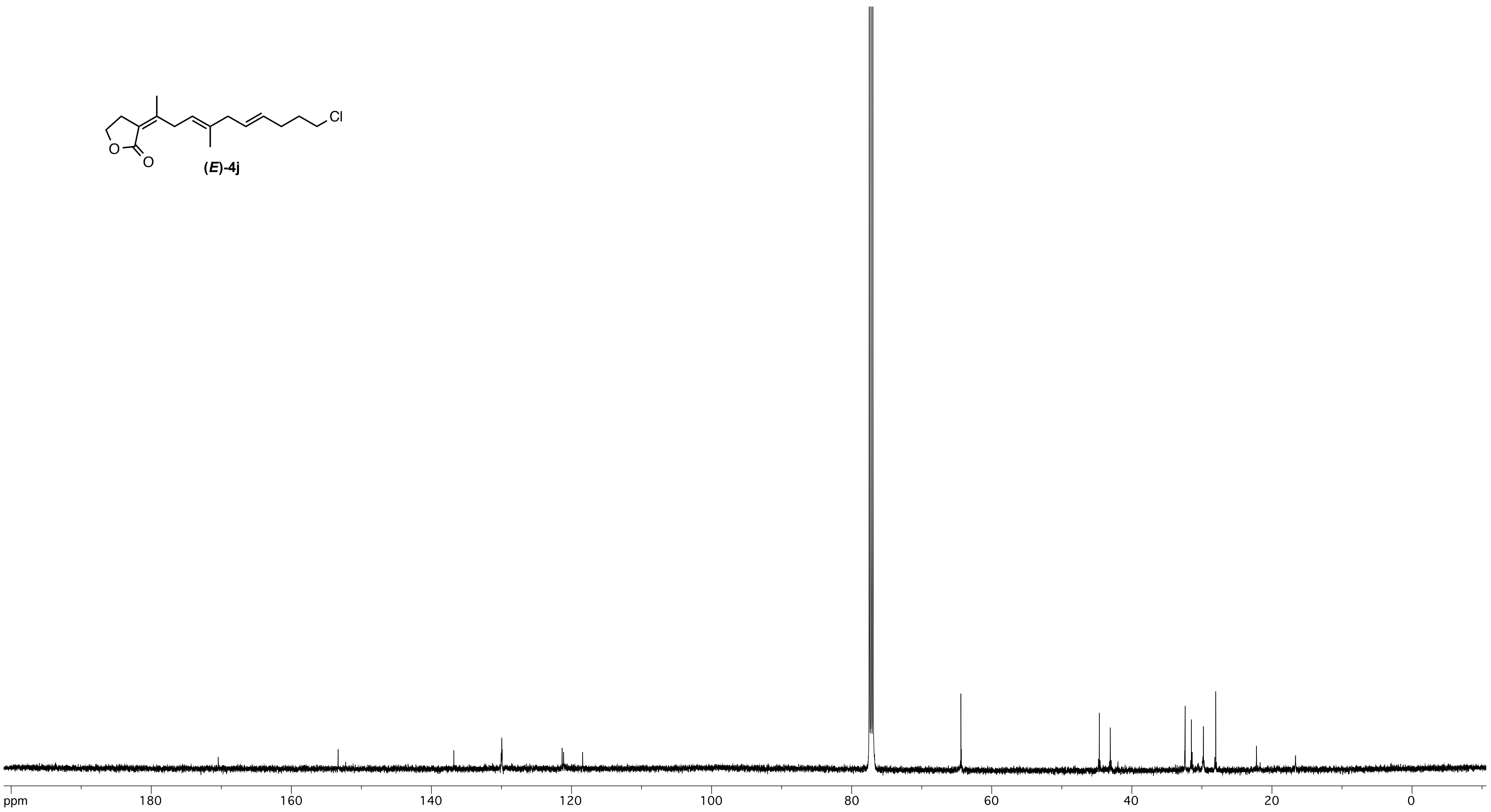
Cl
O
O
(E)-4j
ppm
180
160
140
120
100
80
60
40
20
0
